# *Bacterial* and *Archaeal* Structural Diversity in Several Biodeterioration Patterns on the Limestone Walls of the Old Cathedral of Coimbra

**DOI:** 10.3390/microorganisms9040709

**Published:** 2021-03-30

**Authors:** Catarina Coelho, Nuno Mesquita, Inês Costa, Fabiana Soares, João Trovão, Helena Freitas, António Portugal, Igor Tiago

**Affiliations:** Centre for Functional Ecology, Department of Life Sciences, University of Coimbra, 3000-456 Coimbra, Portugal; catarinafc89@gmail.com (C.C.); inunomesquita@gmail.com (N.M.); inesoliveiracosta@gmail.com (I.C.); fabiana.alm.soares@gmail.com (F.S.); jtrovaosb@gmail.com (J.T.); hfreitas@uc.pt (H.F.); aportuga@bot.uc.pt (A.P.)

**Keywords:** limestone, NGS analysis, biodeterioration, old cathedral of Coimbra, bacteria and archaea diversity

## Abstract

The “University of Coimbra-Alta and Sofia” area was awarded the UNESCO World Heritage Site distinction in 2013. The Old Cathedral of Coimbra, a 12th-century limestone monument located in this area, has been significantly impacted during the last 800 years by physical, chemical, and biological processes. This led to the significant deterioration of some of its structures and carvings, with loss of aesthetical, cultural, and historical values. For this work, deteriorated spots of the walls of three semi-open chapels from the cloister of the Cathedral were sampled to ascertain their bacterial and archaeal structural diversity. Based on Next-Generation Sequencing (NGS) result analysis, we report the presence of microbial populations that are well adapted to an ecosystem with harsh conditions and that can establish a diverse biofilm in most cases. While it was possible to determine dominant phylogenetic groups in *Archaea* and *Bacteria* domains, there was no clear connection between specific core microbiomes and the different deterioration patterns analyzed. The distribution of these archaeal and bacterial communities within the analyzed biodeterioration spots suggests they are more influenced by abiotic factors (i.e., water availability, salinity, etc.), although they influence (and are influenced by) the algal and fungal population composition in this ecosystem. This work provides valuable information that can assist in establishing future guidelines for the preservation and conservation of this kind of historic stone monuments.

## 1. Introduction

During the last decades, several endolithic and epilithic microbial communities have been reported in limestone monuments worldwide, which display diverse biodeterioration patterns depending on the different environmental conditions [1,2,3,4]. The most relevant factors affecting this colonization are physical (humidity, temperature, sunlight exposure, substrate porosity) and chemical (environmental pollution, substrate composition) [5,6,7]. Different supports with different physicochemical properties can condition the establishment of specific communities. Moreover, the synergistic relationship with other colonizing organisms, as well as with the environmental conditions and urban pollution, can also impact the microbial community composition, and consequently influence their contribution to stone biodeterioration [5,6,7,8,9].

Limestones are susceptible to host microbial growth in temperate climates, given their high porosity and permeability, and consequent capacity to retain environmental moisture [10]. The historical complex ‘University of Coimbra–Alta and Sofia’ (Portugal) UNESCO World heritage site includes several limestone monuments affected by various biodeterioration phenomena. A good example is the Old Cathedral of Coimbra (“Sé Velha de Coimbra”) (40°12′32″ N, 8°25′38″ W), a 12th Century Romanesque church, and one of the most iconic and frequently visited monuments in the city. The church was built using mostly yellow dolomitic limestone (i.e., carbonate rock principally composed of calcium magnesium carbonate) from nearby quarries [9,11], and its single-floored cloister contains five semi-open chapels (in the north gallery, chapel of the altarpiece “Natal do Senhor”; in the east gallery, chapels of “São Miguel”, “Santa Cecília”, and “Santa Maria”; and in the south gallery, the chapel of “São Nicolau/Santa Catarina”), deeply affected by biodeterioration phenomena [12,13].

Over the last 800 years, a significant impact was exerted on this historic monument, by physical, chemical, and biological processes. These processes have led to the development of various deterioration patterns in some of its structures and carvings, with associated losses of aesthetical, cultural, and historical value. Nonetheless, in the last decades, several restoration interventions have been performed in this monument, in an effort to minimize their impact. Currently, the principal visible signs of deterioration observed in this monument are related to stone weathering and salt efflorescence but also the biological proliferation in the form of green biofilms and dark discolorations. These have severely affected the limestone walls, both structurally and aesthetically, either by the mere presence of these organisms and their pigments, or by pitting, flaking, and opening of fissures.

An additional problem, which is likely contributing to the development of these organisms, is water infiltration running through the walls in some of the chapels (“Santa Maria” and “São Nicolau/Santa Catarina”), with an almost permanent runoff. Moisture facilitates biofilm development and allows the mobilization of salts to the stone surface, leading to localized salt efflorescence formation (mainly gypsum). It is known that the deterioration of inorganic materials in open environments is usually a result of the activity of a few species of microorganisms [7,14,15,16], and to understand how the combined degradation phenomena take place, it is important to identify the established microbial communities, how they interact with each other and with the stone support. The diversity and characteristics of the initial colonizers (usually, photoautotroph and chemoautotroph organisms) [17], which are responsible for the biofilm formations, are probably more dependent on the existing physicochemical conditions than the organisms that later use the biofilms to thrive, since various niches are made available by the microenvironments that biofilms themselves provide.

As reported by Soares and colleagues [13], the development of photosynthetic organisms in these sites can lead to water retention and increased carbon availability in biofilms. This fact provides the support for other microorganisms to follow, such as fungi, bacteria, and archaea. The diversity of photosynthetic and fungal organisms in this site was already determined [12,13], and their role in the biodeterioration of the limestone walls was discussed. Although bacteria and archaea are known to also contribute to stone biodeterioration through acidolysis, the removal of cations, and the promotion of mineralization development [7,18], their diversity in these sites remains pending a deep characterization.

The present work focused on the sampling of three chapels (displaying severe signs of biodeterioration) from the semi-open environment of the cloister of the Old Cathedral of Coimbra, with different spatial orientations and sunlight exposure. It aimed to assess the structural microbial diversity in degraded limestone walls using Next-Generation Sequencing (NGS) analysis to study the potential interactions between the different microbial populations and their compositions, and to determine possible “microbial population” vs. “specific biodeterioration pattern” correlations. This work will provide further knowledge and thus a better understanding of the microbial communities responsible for the biodeterioration of limestone walls, contributing to the development of appropriate prevention and restoration treatments in the future.

## 2. Materials and Methods

### 2.1. Site Description and Sample Collection

The cloister of the Old Cathedral of Coimbra comprises five chapels, from which three were sampled in this study—Chapels of “São Miguel”, “Santa Maria”, and “São Nicolau”/”Santa Catarina”. Since the chapel of the altarpiece “Natal do Senhor” did not display signs of significant biodeterioration, and the Chapel of “Santa Cecília” is permanently closed to the public, they were not a part of this study.

A total of ten samples were collected from areas showing visible signs of biodeterioration in November of 2016. The origin of each sample as well as a short description of the biodeterioration phenomena encountered is displayed in Table 1 (color photographs of each sampled area are available in Supplementary Material Figure S1). Samples were scraped off the center of the biofilms (±3 cm^2^) into sterile tubes, at about 2 m above ground level, and they were transported on ice to the laboratory, where they were immediately processed and cryopreserved at −80 °C until further analysis. All sampling procedures were performed with the permission of the “Direcção Regional de Cultura do Centro” (the local government authority) and with the supervision of technicians from the Cathedral.

### 2.2. DNA Extraction, Sequencing, and Phylogenetic Analysis

Genomic DNA was extracted using a MoBio DNeasy Powerlyzer DNA isolation Kit (Qiagen, The Netherlands), following the manufacturer’s instructions. DNA concentration and quality were determined using Nanodrop (Thermo Scientific) and by a 1% agarose gel electrophoresis, respectively. Genomic DNA was stored at −20 °C until further analysis. Ten genomic DNA samples were sequenced through the Illumina MiSeq V2 platform (RTL Genomics, Texas, USA). Bacterial diversity was determined by the amplification of hypervariable regions V3–V4 of the bacterial 16S rRNA gene using forward primer 5′-CCTACGGGNGGCWGCAG-3′ and the reverse primer 5′-GACTACHVGGGTATCTAATCC-3′ [19], and archaeal diversity was determined using forward primer 5′-CCCTAYGGGGYGCASCAG-3′ and the reverse primer 5′-GGACTACVSGGGTATCTAAT-3′ [20]. The raw data were analyzed using mothur v.1.42.1 software package [21]. Briefly, sequences were subjected to conservative quality control measures, namely initial quality trimming and assembly of contig reads sequences. All sequence reads with low quality and ambiguous bases or chimeras were removed from the datasets. The obtained sequences were aligned and clustered into Operational Taxonomic Units (OTUs) with 97% similarity. All high-quality sequences were taxonomically classified through ARB-Silva taxonomic database v138 [22,23] to be used in the mothur software. The bacterial and archaeal gene Illumina sequencing data are deposited in the NCBI BioProject library (Accessions: SRR13558867-SRR13558849). The bacterial and archaeal taxonomic classification and abundance tables are presented as (supplementary materials Tables S2–S9).

### 2.3. Statistical Analysis

Three alpha-bacterial diversity indexes (Inverse Simpson, Shannon, and Shannon-Evenness), richness index (Chao estimator), and the coverage values were generated with mothur software. 

To ascertain the possible presence of microbial population-specific biodeterioration pattern correlations, two principal component analyses (PCA) were performed with the relative abundance of the OTU obtained at 97% similarity value (species level) and biodeterioration patterns. For this purpose, biodeterioration pattern correspondence was the same used by Trovão and colleagues [12], since the sample pool was the same in both works. The principal component analysis (PCA) and corresponding dendrograms were performed using the Canoco v4.5 package [24]. In these analyses, the different samples were plotted according to their OTU composition and distribution (samples with more similar microbiomes are plotted closer to one another); then, the biodeterioration patterns were also plotted, taking into account the characteristics of each sample, to assess a potential relationship between the species composition in each sample, and the biodeterioration phenomena observed.

## 3. Results

### 3.1. Illumina Sequencing Data Analysis

The ten genomic DNA samples were subjected to 16S rRNA sequence analysis using the Illumina MiSeq V2 platform. All samples provided good results for domain *Bacteria*; however, despite several attempts, sample SV8 failed to produce any amplification results for domain *Archaea*, whereas only nine sites were considered for this domain. All coverage values were at a minimum of 99.4% (Table 2 and Table 3), providing evidence that the diversity obtained was representative. After quality control, a total of 277,371 high-quality sequences for *Bacteria* and 93,394 for *Archaea* were selected for further analysis. These sequences were assigned from phylum to genus taxonomic ranks according to the SILVA taxonomic database, using mothur v.1.42.1 software [21].

For domain *Bacteria*, the high coverage values and the closeness between the Chao index and the obtained OTUs (Table 2) showed that most bacterial sequences were successfully retrieved in this process, where the highest number of OTUs was obtained in SV1 (819 OTUs), and the lowest was obtained in SV5 (76 OTUs). In what concerns alpha diversity indexes, the highest Inverse Simpson values were obtained in SV2 and the lowest were obtained in SV5. In terms of distribution, the Shannon–Evenness values were lowest in SV5, and higher for SV2 and SV4. These results suggest that bacterial populations of SV2 and SV4 were more equally distributed than in the remaining samples. Overall, 5050 bacterial OTUs were assigned to 29 phyla, 88 classes, and 388 families, while only <1% of sequences were not assigned to any known phylum (Supplementary Materials: Tables S1, S3, S5, S7, and Figure S2).

In domain *Archaea*, the high coverage values (99.8–100%), as well as the closeness between the Chao index and the obtained OTUs (Table 3), also showed that most of the archaeal sequences were successfully retrieved in this process. The highest number of OTUs was obtained in SV5 (37 OTUs), and the lowest was obtained in SV9 (5 OTUs). The Inverse Simpson values were relatively similar for all samples. The lowest Inverse Simpson value was obtained in SV9 and the highest was obtained in SV4. In terms of distribution, SV9 showed the lowest Shannon–Evenness values while the highest was SV4. Overall, 69 archaeal OTUs were assigned to three phyla or six classes, and only <0.05% of sequences were not assigned to any classified phylum (Supplementary Materials: Tables S2, S4, S6, S8, and Figure S3).

### 3.2. Phylogenetic Analysis

#### 3.2.1. Domain Bacteria

**Figure 1:**
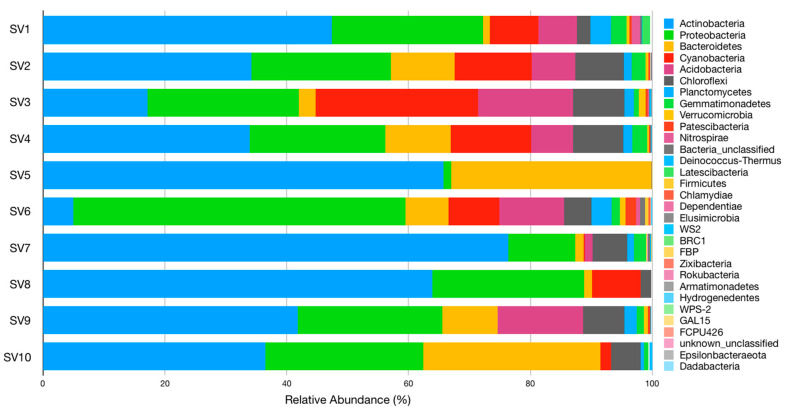
Relative abundance and distribution of bacterial phyla in each sample.

From the twenty-nine phyla identified, six were highly abundant, comprising at least 89% of the sequences in each sample. These were *Actinobacteria*, *Proteobacteria*, *Bacteroidetes*, and *Cyanobacteria* (all were found in all samples), *Acidobacteria* (all but SV5 and SV10), and *Chloroflexi* (all but SV5). Phyla *Gemmatimonadetes* and *Planctomycetes* were also identified in all samples except SV5; however, their distribution (relative abundance) was quite different between samples (Figure 1).

Phylum *Actinobacteria* encompassed major populations belonging to the families *Rubrobacteriaceae* and *Pseudonocardiaceae*. Family *Rubrobacteriaceae*, whose members are usually radio tolerant and very resistant to desiccation [25], was the most frequent family in SV2 (12.3%), SV4 (13.5%), SV5 (65.3%), SV8 (34.4%), SV9 (24.8%), and SV10 (26.5%), but it was not dominant in SV3 (3.8%) nor SV7 (11.3%). This family was represented by a single genus, *Rubrobacter*, which was the most prominent bacterial genus when considering all sites, and it was found (with relative abundance >3%) in eight out of the 10 samples: it was predominant in SV2 (12.3%), SV4 (13.5%), SV5 (65.3%), SV8 (34.4%), SV9 (24.8%), and SV10 (26.5%), and it was not predominant in SV7 (11.3%) and SV3 (3.8%). Family *Pseudonocardiaceae* was most abundant in SV1 (27.97%, 99% of which were of genus Crossiella) and SV7 (46.72%, 99% of which were of genus *Crossiella*), and in all remaining samples with relative abundance > 2.7% except for samples SV5 and SV6 (< 0.5%). Genus *Crossiella* was clearly the main representative of this family, while genera *Actinomycetospora* and *Actinokineospora* and *Pseudonocardia* were present, but with lower relative abundance.

Apart from the two above-mentioned families, *Intrasporangiaceae* was major in SV8 (10.9%), mainly represented by genus *Ornithinimicrobium* (8.4%) and other unclassified sequences (1.4%), while clone order 0319-7L14 (family and genus) was predominant in SV1 (11.8%).

One exception to the dominance of phylum *Actinobacteria* was verified in SV3–where phylum *Cyanobacteria* was dominant (26.6%), with most sequences belonging to an uncultured family. Sequences of an unclassified family from class *Oxyphotobacteria* were identified in samples SV1 (7.9%), SV2 (8.2%), SV4 (7.8%), and SV8 (4.2%). Furthermore, an unknown family belonging to order *Nostocales* (class *Cyanophyceae*) was found in samples SV2 (3.3%), SV3 (1.0%), SV4 (4.3%), and SV10 (1.7%).

The other exception to the dominance of phylum *Actinobacteria* was in SV6, where *Proteobacteria* were dominant (54.5%), from which ≈52% belonged to class *Gammaproteobacteria* (mostly family *Burkholderiaceae*) and ≈48% belonged to class *Alphaproteobacteria* (mostly family *Sphingomonadaceae*). Moreover, the relative abundance of phylum *Proteobacteria* was higher than 20% in seven of the ten samples and was the second most abundant overall (≈22%) as displayed in Figure 1 and supplementary materials (Tables S1, S3, S5, S7, and Figure S2).

Members of family *Burkholderiaceae* were detected in all samples except SV5 and SV10 and were more frequent in samples SV6 (17.3%) and SV9 (2.4%), although they were mainly represented by unclassified sequences and belonged to uncultured populations. 

Representatives of the family *Sphingomonadaceae* were detected in all samples (except SV5), but many represented unclassified sequences. However, genus *Qipengyuania*, from which the only known species is *Qipengyuania sediminis* [26], was detected in SV2 (1.2%), SV3 (1.2%), SV4 (0.9%), and SV9 (4.7%). In addition, genus *Sphingomonas* was detected in SV3 (3.5%), SV4 (0.9%), SV8 (6.4%), and SV10 (1.3%), genus *Altererythrobacter* was detected in SV6 (3.2%) and genus *Novosphingobium* was detected exclusively in SV10 (11.4%).

Sequences belonging to family *Rhodobacteraceae* were detected in samples SV2 (2.0%), SV3 (5.8%), SV4 (1.9%), SV6 (1.5%), and SV9 (4.1%), but they represented mostly unclassified or uncultured populations, with the exception being genus *Rubellimicrobium*, the dominant genus in sample SV9 (3.8%).

Phylum *Bacteroidetes* was the third most abundant. It was mainly represented by family *Rhodothermaceae*, which was detected in SV9 (2.0%, of mainly unclassified sequences), and SV10 (17.3%, fully represented by genus *Rubrivirga*), but also in other samples, but with low relative abundance (<0.5%). Family *Flavobacteriaceae* was essentially found as a single unidentified genus in SV5 (4.2%); family *Balneolaceae* was represented essentially by sequences belonging to an unclassified genus found in SV5 (28.5%), SV2 (6.8%), and SV4 (6.8%); family *Cyclobacteriaceae* is one of the most prominent families in SV10 (8.3%), and it was mainly represented by genus *Tunicatimonas* (7.9%); and family *Chitinophagaceae* was only identified in samples SV3 (1.3%) and SV9 (5.8%), and it was mostly represented by genus *Flavisolibacter*.

Family *Blastocatellaceae* was identified in samples SV1 (4.5%, mostly represented by unclassified and uncultured populations), SV2 (5.1%, mostly represented by genus *Blastocatella*), SV3 (12.6%, mostly represented by genus *Blastocatella* and by unclassified taxa), SV4 (4.8%, mostly represented by genus *Blastocatella*), SV6 (6.2%, mostly represented unclassified and uncultured populations), and SV9 (9.3%, mostly represented by genus *Blastocatella*).

Other phyla were found in most samples but with lower relative abundance values. Phylum *Chloroflexi* encompassed thirty distinct families, all with relative abundances values <1%, and it was detected in all samples, except SV5. Within phylum *Gemmatimonadetes*, families *Gemmatimonadaceae* and *Longimicrobiaceae* were the most dominant. 

Phylum *Planctomycetes* was present in all samples except SV5, which was represented by several families, all below 1% relative abundances, except for families *Tepidisphaeraceae* (1.1% in SV9) and *Gemmataceae* (1.1% in SV6).

In general, there was a low dominance of specific families in most samples: 10 to 20 families usually added up to ≈80% of the organisms in each sample (Supplementary Materials: Tables S1, S3, S5, S7, and Figure S2). The exception was sample SV5, which was dominated by three families: *Rubrobacteraceae* (65.3%), *Balneolaceae* (28.5%), and *Flavobacteriaceae* (4.2%) summing up to 98% of all organisms, and with each remaining family representing less than 0.5%.

#### 3.2.2. Domain Archaea 

**Figure 2:**
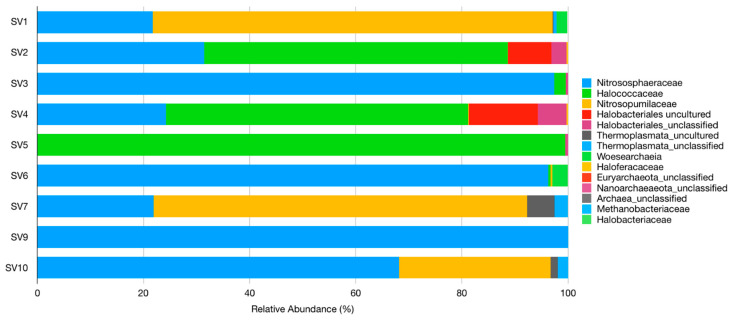
Relative abundance and distribution of archaeal families in each sample.

The major archaeal populations were distributed in two classes, *Haloarchaea* (phylum *Euryarchaeota*) and *Nitrososphaeria* (phylum *Thaumarchaeota*) (Figure 2 and Supplementary Material Figure S3). The dominant families were *Nitrososphaeraceae*, *Halococcaceae,* and *Nitrosopumilaceae*, representing approximately 95% of the total relative abundances in all samples (Supplementary Materials Tables S2, S4, S6, S8, and Figure S3).

*Nitrosophaeraceae* was the most abundant family and was dominant in SV3, SV6, SV9, and SV10, essentially represented by ‘*Candidatus Nitrocosmicus*’. *Nitrosopumilaceae* was the dominant family in SV1 and SV7, and it was almost exclusively represented by ‘*Candidatus Nitrosotenuis*’. *Halococcaceae* was the dominant family in SV2, SV4, and SV5, and it was mostly composed of genera *Hallococcus* and *Halalkalicoccus* (especially in SV4).

Overall, the three most dominant genera of domain *Archaea* were *Halococcus*, ‘Candidatus *Nitrosotenuis*’, and ‘Candidatus *Nitrocosmicus*’, although *Halococcus* was only detected in the chapel of “Santa Maria”, SV5 (99.3% relative abundance), SV2 (55.7%), and SV4 (52.2%) (Supplementary Materials Tables S2, S4, S6, S8, and Figure S3).

Sequences belonging to genus ‘*Candidatus Nitrosotenuis*’ (family *Nitrosopumilaceae*) were dominant in SV1 (64.6%) and SV7 (70.3%), and they were the second most dominant group in SV10 (28.5%). Sequences related to genus ‘*Candidatus Nitrocosmicus*’ (family *Nitrososphaeraceae*) were detected in all samples except SV5; it was dominant in SV3 (92.2%), SV5 (94.6%), SV9 (99.7%), and SV10 (60.8%), and it was second most dominant in SV2 (31.3%), SV4 (24.2%), and SV7 (20.9%).

### 3.3. Principal Component Analysis (PCA)

**Figure 3:**
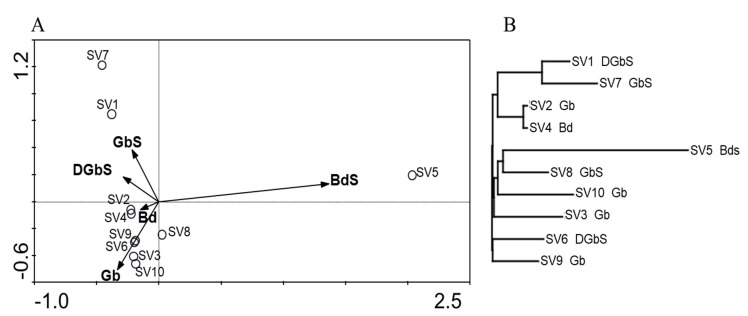
(**A**) Principal component analysis (PCA) and (**B**) neighbor−joining dendrogram based on the Euclidean distance for domain *Bacteria* Operational Taxonomic Units (OTU) profiles. Biofilm classification was used as an environmental variable for PCA and is displayed with each sample designation in the dendrogram (GbS: Green biofilm with visible salt efflorescence; Gb: Green biofilm; DGbS: Dark and green biofilm with visible salt efflorescence; Bd: Black discoloration).

**Figure 4:**
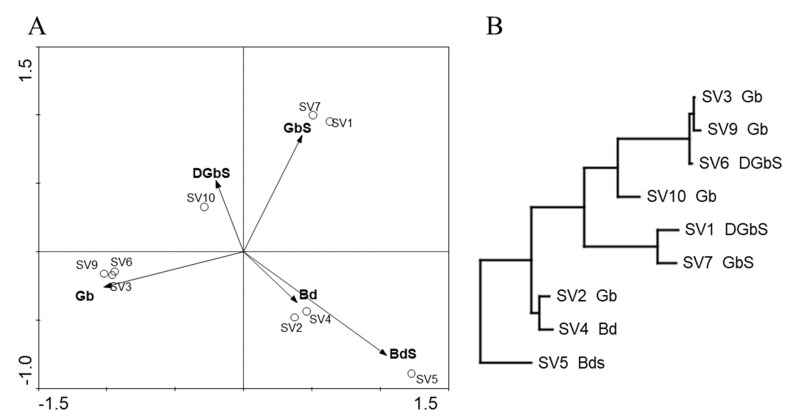
(**A**) Principal component analysis (PCA) and (**B**) neighbor−joining dendrogram based on the Euclidean distance for domain *Archaea* OTU profiles. Biofilm classification was used as an environmental variable for PCA and is displayed with each sample designation in the dendrogram (GbS: Green biofilm with visible salt efflorescence; Gb: Green biofilm; DGbS: Dark and green biofilm with visible salt efflorescence; Bd: Black discoloration).

Figure 3 and Figure 4, for domains *Bacteria* and *Archaea*, respectively, display the results of the principal component analysis (PCA) that was performed with the relative abundance of the OTU obtained at 97% similarity value (genus level) and biodeterioration patterns encountered.

#### 3.3.1. Domain Bacteria

For domain *Bacteria*, a direct correlation between specific population profiles (from each sample) and biodeterioration patterns was not verified consistently. Except for samples SV1, SV7, and SV5 that were distant from the remaining samples, all others were clustered together, regardless of the degradation pattern where they were collected from. Samples SV1 and SV7 were collected in different chapels but clustered together in the PCA and Euclidean similarity dendrogram, indicating a similar composition and distribution. Sample SV5 represented the only sample classified as “black discoloration with salt efflorescence”, and was plotted separately from all others, given its exclusive species composition. Despite the remaining samples (SV2, SV3, SV4, SV6, SV8, SV9, and SV10) being clustered together, it was not possible to establish a core microbiome by comparing their bacterial OTU profiles in mothur (data not shown).

#### 3.3.2. Domain Archaea

For domain *Archaea*, a direct correlation between specific population profiles (in each sample) and the biodeterioration patterns was also unclear. Samples were clustered in a more dispersed pattern than in domain *Bacteria*, and while it could be argued that polarity in the dispersion was present, with samples related to a black discoloration (SV4 and SV5) being in the same quadrant, sample SV2 is plotted close to SV4, and it is not related with the biodeterioration pattern assigned to the latter, depicting a similarity in species composition, which means this composition is not exclusive to either of these biodeterioration types. Samples SV1 and SV7 clustered together, as did samples SV3, SV6, and SV9, but without a clear distribution pattern.

## 4. Discussion

This study aimed at the determination of bacterial and archaeal community structural diversities and their distribution on dissimilar biodeterioration patterns, which were found on the limestone walls of three semi-open chapels from the cloister of the Old Cathedral of Coimbra.

The biodeterioration of limestone, and more specifically of limestone walls of monuments, is a result of the synergetic and complex interactions between abiotic and biotic factors that leads to their structural and aesthetical deformation [9]. Limestone is a carbonate sedimentary porous rock that is primarily composed of calcite and aragonite (two different crystal forms of calcium carbonate), and when water flows within its pores, it sometimes carries solubilized salts to the surface [9]. With time, due to temperature and humidity fluctuations, these salts react with atmospheric polluters i.e., SOx (sulfur oxides), NOx (nitrogen oxides), and COx (carbon oxides) and precipitate over the exposed surfaces (mainly in the form of gypsum), and this crystallization process, eventually, leads to material disruption and loss [27,28,29]. In the studied area, there was visible distinct biofilms development in the different sampled spots (images available in Supplementary Materials Figure S1), which are differences that could be related to sunlight exposure and water availability as well as other biotic and abiotic factors [7,13,30]. Nevertheless, our results support the assumption that water is most likely one of the main factors shaping the microbiome composition and thus the biofilm development, since in wetter spots (where water is available all year), the presence of more developed biofilms was observed, while less humid spots were characterized by the occurrence of shallower and not so developed biofilms. This observation was also thoroughly described in other works characterizing the microbiome of biodeterioration events in limestone and other stone-built monuments [7,15,31,32]. Moreover, due to the ubiquitous presence of salt efflorescence in the sampled deteriorated areas, it was very difficult to classify the deteriorated patterns according to their water availability (moisture) or salt content, since they are in the same semi-open structure, and most of them exhibit chimeric abiotic characteristics [12,13]. For the PCA and Euclidean distancing dendrogram analyses, the biofilm classification by Trovão and colleagues [12] was used as environmental data, and results showed no evidence of a direct correlation between bacterial or archaeal populations and a specific biodeterioration or biofilm classification. Instead, the fact that different kinds of biofilm were found in the different deterioration spots suggests the existence of diverse ecological niches, which is a characteristic that modulates the establishment of the populations and their composition. All these variables help to explain why a core microbial population colonizing limestone in this site could not be determined, but a rather diverse and randomly distributed bacterial and archaeal diversity was observed. Still, the presence of some major bacterial and archaeal populations was observed, which was most likely due to the extremophilic nature of these ecological niches, leading to competition and natural selection, and favoring the settlement of extremophilic organisms. One can relate the number of OTUs and their evenness with the level of maturation of the biofilm, of which the most mature were those with higher diversity (as are examples SV1, SV3, and SV4). The presence of dominant populations also seems to be related to this, since less exuberant and immature biofilms tend to favor the dominance of specific groups. This is more evident in domain *Archaea*, where highly dominant populations were detected (some accounting more than 90% of total abundance in some samples) than in domain *Bacteria* where such high dominance by a specific population was not observed in most of the samples. This observation was also reflected in the physiological diversity observed in both domains, where domain *Archaea* was less diverse than *Bacteria* but with highly physiologically specialized populations, whereas domain *Bacteria* populations were highly diverse, constituting mainly by extremophiles, with lower physiological specificity. Nevertheless, the extremophile nature of most of the populations can be seen as a common feature in both domains. Considering domain *Archaea*, two major groups were observed: one encompassing members belonging to *Euryarchaeota* (class *Halobacteria*), a phylogenetic branch of extreme halophilic archaea, previously found in stone, from limestone surfaces, and areas with salt efflorescence [28,33,34,35,36,37]. Some of these microorganisms produce carotenoid pigments (e.g., β-carotene, α-bacterioruberin and derivatives, and salinixanthin) in their cell membranes [38], and therefore, their proliferation may be responsible for some of the observed rosy stains on some of the wall surfaces [7,39]. The second group encompassed members of *Thaumarchaeota* (class *Nitrososphaeria*), which is a taxonomic group that is considered to have a dominant role in the oxidation of ammonia, being designated as ammonia-oxidizing archaea (AOA). Ammonia-oxidizing archaea are ubiquitously detected in natural environments such as open ocean [39] and soils [40,41,42,43], where they have an active role in the nitrogen cycle. They also have been detected in the surface of stone building where their presence and activity has been related to events of biodeterioration [44,45,46]. Additionally, as previously described [7,47] their specific metabolism (ammonia oxidation) is responsible for the formation of acidic bioproducts that, in our specific case, can promote the acid-induced carbonate weathering of the limestone walls, thus contributing to the biodeterioration events observed in the Old Cathedral of Coimbra. Their presence in sandstone monuments and their relevance in biofilms has been addressed by several authors recently [44,48,49], but to our knowledge, this is the first time this taxonomic group is reported in limestone walls, providing evidence of the uniqueness of this specific ecosystem. In our study, members of the first group (halophilic archaea) were detected in some of the driest samples (SV2, SV4, and SV5), while members of the second group (AOA) were detected in almost all samples, except for SV5 (the driest sample) and SV8 (where no archaeal populations were detected). So, the presence of a high concentration of halophiles in the dryer samples could be related to the presence of less-developed green biofilms and higher salinity, while the higher concentration of AOA was related to more humid samples with lower halophilic conditions (due to water leaching), where the green biofilms were most exuberant. This suggests that AOA have an important role in biofilm formation, allowing the transition of less exuberant to the more exuberant and diverse biofilms as well as acting as a precursor and helper on biofilm formation, which has been previously described by other colleagues [44,50]. Additionally, AOA are more prone to live in open environments between plant canopies (i.e., bare soil) that represent oligotrophic environments [51], which is most likely similar in nutrient conditions existing in our biofilm samples from limestone stone walls. Overall, the detected AOA probably represent oligotrophic, stress-tolerant organisms that verify the extreme environment that the limestone stone walls surface represents for microbial life, contributing to biodeterioration events of the stone walls by supporting biofilm settlement and maintenance, and by promoting carbonate weathering of the limestone walls.

As stated above, the structural diversity determined to domain *Bacteria* did not show an organized distribution of its populations that was likely more influenced by abiotic conditions, as well as the biofilm development itself. This observation is supported by the lack of correspondence that is observed on the PCA analysis (Figure 3). Despite that, some of the major populations detected are known for their extremophilic characteristics such as halophily, showing that salinity also plays a role in bacterial population establishment, and it was therefore not surprising to see those associated with samples that displayed more selective and extreme environments. Genus *Rubrobacter*, almost ubiquitous, was dominant in samples SV2, SV4, and SV5 (and one of the main populations in SV7, SV8, SV9, and SV10), which was probably due to its extremophilic characteristics, namely, its high tolerance to high salt concentrations. *Rubrobacter* has been appointed as a causative agent of stone deterioration and discoloration, forming pink areas where it thrives [52,53,54], and it is likely to play an active role in salt efflorescence phenomena and mineral precipitation [55]. *Rubrobacter* was recently found associated with limestone by Schröer et al. [56], where it was the dominant population in samples from a rather low polluted environment. In addition, a new *Rubrobacter* species was identified in a Portuguese stone monument [55], which reinforces the relevance of these works in assessing the degradation of limestone and other materials used in cultural heritage.

Genus *Crossiella* (family *Pseudonocardiaceae*) was the major population in samples SV1 and SV7, both displaying biofilms with salt efflorescence. Members of this genus are extremophiles and have been associated with CaCO_3_ precipitation and white crust formation (as seen in SV1 and SV7) in stone monuments [16]. 

Photosynthetic microorganisms and algae are considered the first colonizers of stone building, and by providing the substrate for the heterotrophic populations, such as fungi, bacteria, and archaea to settle, contributing to biodeterioration events in the walls [7,15,57]. Our results showed that there was a considerable fraction of phylum *Cyanobacteria* within many of our samples, and that these were even dominant in sample SV3 (one with the most exuberant biofilm). We believe this to be a consequence of the available water in this semi-closed environment, which lowers the salinity by leaching and supports more developed biofilms. Our results are, in that sense, concordant with other recent microbial population studies in stone buildings [15,58,59]. For domain *Bacteria*, genera *Cyanobacteria* and *Chloroflexi* are likely the key photoautotrophic microorganisms colonizing the limestone surface, which was found in every sample except for the driest one, SV5. These populations, along with photosynthetic *Eukarya* described more thoroughly by Soares and colleagues [13], contribute to the establishment of heterotrophic communities such as fungi [12], bacteria, and archaea in this limestone monument, and by that, besides the aesthetic problems, contribute to the biodeterioration events observed (Supplementary Materials Figure S1).

The remaining bacterial populations were oddly distributed, contributing to the phylogenetic richness that characterized most samples. We believe this is related to the semi-open environment and the existing structural damage [12], which enhances direct interaction with water, most likely the main vector for microbial cell transportation [13]. Some of the less ubiquitous populations but still present in relatively high abundance in some samples belonged to families *Balneolaceae*, *Blastocatellaceae*, *Burkholderiaceae*, *Cyclobacteriaceae*, *Intrasporangiaceae*, *Rhodobacteraceae*, *Rhodothermaceae*, and *Sphingomonadaceae*. Members of *Sphingomonadaceae*, a family of the *Alphaproteobacteria*, encompass some phototrophic species, and some species of this family are known for their ability to degrade some aromatic compounds [60]. Genus *Sphingomonas* includes several species that are quite diverse in terms of their phylogenetic, ecological, and physiological properties. These are widely distributed in nature and were described as major players in the deterioration of limestone monuments [58]. Still from within this family, members of genus *Altererythrobacter* have been isolated from sand and deep sediment [61,62], air [63], and seawater [64] as well as other materials, and they range from halophilic to halotolerant [62].

Family *Blastocatellaceae* encompassed populations with relevant abundance in some samples, and its members are known to accommodate oligotrophic, slightly acidophilic to neutrophilic mesophiles previously isolated from arid soils as well as anoxygenic photoheterotrophic bacteria previously isolated from microbial mats [65]. Genus *Blastocatella* has a single classified species, *Bastocatella fastidiosa*, and it was the predominant genus whenever members of family *Blastocatellaceae* were found. Furthermore, it has been identified in other stone monuments [16]. As previously stated, members of the *Intrasporangiaceae* family were mainly detected with very low relative abundance values, except for sample SV8 (10.9%), where it was represented essentially by members belonging to the genus *Ornithinimicrobium*. This genus is composed of seven different species that have been isolated from different materials, such as indoor walls colonized by molds [66], plant leaves [67], or even aquaculture systems [68]. 

Members belonging to genus *Rubrivirga*, comprising two known halophilic species isolated from deep-sea water [69,70], were found essentially in sample SV10. Additionally relevant in this sample was genus *Tunicatimonas* (family *Cyclobacteriaceae*), which has a single known species, *Tunicatimonas pelagia*, previously isolated from sea anemone [71]. *Rubellimicrobium*, a member of the family *Rhodobacteraceae*, was found dominant in sample SV9. This genus has four known species that have been isolated from different sources, namely soil [72] and air [73] samples.

When comparing our results with other studies that also used NGS approaches to determine the microbiome composition [48,49,55,59], we observe that apparently, our study detected a broader microbial structural diversity inhabiting the sampled biofilms. Domain *Bacteria* was dominated by members belonging to phyla *Actinobacteria* and *Proteobacteria*, and with relevant presence of *Cyanobacteria*, *Bacteroidetes*, *Acidobacteria*. The presence of members of domain *Archaea* in almost all samples add to the detected diversity. Some of these studies have detected subsets of these organisms, but in our case, probably because of the rather stable environment, lighting conditions, and water availability, these groups could develop in well-established biofilms. The exception to this was sample SV5, which, given its location on the monument that confers a dry condition and higher salt efflorescence content, did not present a well-developed biofilm and rather lower diversity.

## 5. Conclusions

Thorough identification and characterization of the bacterial and archaeal populations in the limestone walls of the studied monument were attained in this study. That included a broad range of taxonomic groups, retrieved from a series of spots displaying different biodeterioration phenomena. We believe that salinity and water availability were the main abiotic factors influencing the microbial diversity detected, but that sunlight exposure and biotic interactions influenced the colonization by different groups. Thus, this ecosystem constitutes extreme environments where only populations capable of bearing harsh conditions (i.e., oligotrophy and high salinity) are favored to thrive. The fact that a large sum of the determined populations was, to our knowledge, not previously described in limestone walls, depicts the high diversity that such environments can bear. Accordingly, this work reiterates the need for future isolation-based surveys, tackling bacterial and archaeal populations, in order to study the metabolism of living cultures, and ascertain their putative role in the biodeterioration of the limestone rock walls. It is our opinion that the structural problems in this monument (as well as others), that allow an excess of flowing water within the semi-open structures, have to swiftly be dealt with due to its role in the shaping of the limestone walls abiotic and abiotic characteristics. Since the removal of microbial biofilms and colonies from the surface of stone monuments as “an effective conservation and restoration procedure” is still a topic of debate, future restoring interventions need to take into consideration the potential interactions, not only between the different organisms but most importantly with the abiotic factors of the ecosystem. One of the bigger risks of tinkering with an established and rather stable community is potentially triggering the establishment of even more damaging organisms.

## Figures and Tables

**Table 1:** Sample description: chapel of origin and biodeterioration pattern description.

Sample	Chapel	Biodeterioration Pattern
SV1	São Miguel	Dark and green biofilm with salt efflorescence
SV2	Santa Maria	Green biofilm
SV3	Santa Maria	Green biofilm
SV4	Santa Maria	Black discoloration
SV5	Santa Maria	Black discoloration with salt efflorescence
SV6	São Nicolau/Santa Catarina	Dark and green biofilm with salt efflorescence
SV7	São Nicolau/Santa Catarina	Green biofilm with salt efflorescence
SV8	São Nicolau/Santa Catarina	Green biofilm with salt efflorescence
SV9	São Nicolau/Santa Catarina	Green biofilm
SV10	São Nicolau/Santa Catarina	Green biofilm

**Table 2:** Illumina sequencing statistical results and diversity indexes for domain *Bacteria*.

Sample	Number of Sequences	Coverage (%)	Number of OTUs	Inverse Simpson Index	Shannon Index	Shannon Evenness Index	Chao
SV1	52,067	99.9	819	10.7	3.9	0.6	848.5
SV2	19,049	99.4	682	55.7	4.9	0.8	731.8
SV3	42,057	99.8	752	13.0	4.2	0.6	800.1
SV4	17,833	99.5	657	55.7	4.8	0.8	696.8
SV5	34,500	100.0	76	2.2	1.2	0.3	80.7
SV6	16,288	99.7	510	23.8	4.5	0.7	527.1
SV7	18,248	99.6	517	5.9	3.4	0.6	553.3
SV8	22,581	99.9	237	19.2	3.6	0.7	246.4
SV9	27,420	99.8	475	24.8	4.1	0.7	498.0
SV10	27,328	99.8	325	10.2	3.3	0.6	362.1

**Table 3:** Illumina sequencing statistical results and diversity indexes for domain *Archaea*.

Sample	Number of Sequences	Coverage (%)	Number of OTUs	Inverse Simpson Index	Shannon Index	Shannon Evenness Index	Chao
SV1	17,430	100.0	21	2.2	1.2	0.4	21.0
SV2	1016	99.7	17	3.6	1.6	0.6	18.5
SV3	1644	99.9	11	1.1	0.2	0.1	11.3
SV4	1022	99.8	16	4.3	1.8	0.6	16.3
SV5	35,485	100.0	37	1.4	0.6	0.2	37.0
SV6	9408	100.0	9	1.1	0.3	0.1	9.0
SV7	15,060	100.0	14	1.8	0.9	0.3	15.5
SV9	11,968	100.0	5	1.0	0.0	0.0	5.0
SV10	361	99.7	7	2.2	1.0	0.5	7.0

## Data Availability

Not applicable.

## Supplementary Materials

The following are available online at https://www.mdpi.com/article/10.3390/microorganisms9040709/s1, Figure S1: Visible signs of biodeterioration in different chapels. São Miguel chapel (SV1); Santa Maria chapel (SV2; SV3; SV4; SV5), São Nicolau/ Santa Catarina chapel (SV6; SV7; SV8; SV9; SV10). Figure S2. Relative abundances of bacterial 16S rRNA genes at (a)phylum, (b) class, (c) order (d) family, (e) genus taxonomic ranks. Figure S3. Relative abundances of archaeal 16S rRNA genes at genus taxonomic rank. Legend provides taxonomic classification from domain to genus ranks. Table S1. Taxonomic classification till genus, for domain Bacteria, and respective relative abundances are provided for each sample. Table S2. Taxonomic classification till genus, for domain Archaea, and respective relative abundances are provided for each sample. Table S3. Taxonomic classification till genus for each OTU (operational taxonomic unit) determined for domain Bacteria. Respective relative abundances are provided for each OTU in each sample. Table S4. Taxonomic classification till genus for each OTU (operational taxonomic unit) determined for domain Archaea. Respective relative abundances are provided for each OTU in each sample. Table S5. Taxonomic classification till genus, for domain Bacteria, and respective sequence numbers assigned are provided for each sample. Table S6. Taxonomic classification till genus, for domain Archaea, and respective sequence numbers assigned are provided for each sample. Table S7. Taxonomic classification till genus for each OTU (operational taxonomic unit) determined for domain Bacteria. Respective sequence numbers assigned to each OTU are provided for each sample. Table S8. Taxonomic classification till genus for each OTU (operational taxonomic unit) determined for domain Archaea. Respective sequence numbers assigned to each OTU are provided for each sample.

## References

[1] Gaylarde P.M., Gaylarde C.C. (2000). Algae and cyanobacteria on painted buildings in Latin America. Int. Biodeterior. Biodegrad..

[2] Gaylarde C.C., Ortega-Morales B.O., Bartolo-Perez P. (2007). Biogenic black crusts on buildings in unpolluted environments. Curr. Microbiol..

[3] Ortega-Morales O., Montero-Muñoz J.L., Baptista Neto J.A., Beech I.B., Sunner J., Gaylarde C.C. (2019). Deterioration and Microbial Colonization of Cultural Heritage Stone Buildings in Polluted and Unpolluted Tropical and Subtropical Climates: A Meta-Analysis. Int. Biodeterior. Biodegrad..

[4] Gaylarde C.C. (2020). Influence of Environment on Microbial Colonization of Historic Stone Buildings with Emphasis on Cyanobacteria. Heritage.

[5] Guillitte O. (1995). Bioreceptivity: A new concept for building ecology studies. Sci. Total Environ..

[6] Warscheid T., Braams J. (2000). Biodeterioration of stone: A review. Int. Biodeterior. Biodegrad..

[7] Liu X., Koestler R.J., Warscheid T., Katayama Y., Gu J.-D. (2020). Microbial Deterioration and Sustainable Conservation of Stone Monuments and Buildings. Nat. Sustain..

[8] Mitchell R., Gu J.D. (2000). Changes in the biofilm microflora of limestone caused by atmospheric pollutants. Int. Biodeterior. Biodegrad..

[9] Pinheiro A.C., Mesquita N., Trovão J., Soares F., Tiago I., Coelho C., de Carvalho H.P., Gil F., Catarino L., Piñar G. (2019). Limestone biodeterioration: A review on the Portuguese cultural heritage scenario. J. Cult. Herit..

[10] Miller A.Z.A.F. (2010). Primary Bioreceptivity of Limestones from the Mediterranean Basin to Phototrophic Microorganisms. Ph.D. Thesis.

[11] Catarino L., Figueiredo R., Figueiredo F.P., Andrade P., Duarte J. (2019). The Use of Dolostone in Historical Buildings of Coimbra (Central Portugal). Sustainability.

[12] Trovão J., Portugal A., Soares F., Paiva D.S., Mesquita N., Coelho C., Pinheiro A.C., Catarino L., Gil F., Tiago I. (2019). Fungal diversity and distribution across distinct biodeterioration phenomena in limestone walls of the old cathedral of Coimbra, UNESCO World Heritage Site. Int. Biodeterior. Biodegrad..

[13] Soares S., Portugal A., Trovão J., Coelho C., Mesquita N., Pinheiro A.C., Gil F., Catarino L., Cardoso S.M., Tiago I. (2019). Structural diversity of photoautotrophic populations within the UNESCO site ‘Old Cathedral of Coimbra’ (Portugal), using a combined approach. Int. Biodeterior. Biodegrad..

[14] Lan W., Li H., Wang W.D., Katayama Y., Gu J.D. (2010). Microbial Community Analysis of Fresh and Old Microbial Biofilms on Bayon Temple Sandstone of Angkor Thom, Cambodia. Microb. Ecol..

[15] Prieto B., Vázquez-Nion D., Fuentes E., Durán-Román A.G. (2020). Response of subaerial biofilms growing on stone-built cultural heritage to changing water regime and CO2 conditions. Int. Biodeterior. Biodegrad..

[16] Li Q., Zhang B., Yang X., Ge Q. (2018). Deterioration-associated microbiome of stone monuments: Structure, variation, and assembly. Appl. Environ. Microbiol..

[17] May E., Papida S., Abdulla H., Tayler S., Dewedar A., Ciferri O., Tiano P., Mastromei G. (2000). Comparative studies of microbial communities on stone monuments in temperate and semi-arid climates. Of Microbes and Art: The Role of Microbial Communities in the Degradation and Protection of Cultural Heritage.

[18] Dakal T.C., Cameotra S.S. (2012). Microbially Induced Deterioration of Architectural Heritages: Routes and Mechanisms Involved. Environ. Sci. Eur..

[19] Klindworth A., Pruesse E., Schweer T., Peplies J., Quast C., Horn M., Glöckner F.O. (2013). Evaluation of general 16S ribosomal RNA gene PCR primers for classical and next-generation sequencing-based diversity studies. Nucleic Acids Res..

[20] Zhang J., Lv C., Tong J., Liu J., Yu D., Wang Y., Chen M., Wei Y. (2016). Optimization and microbial community analysis of anaerobic co-digestion of food waste and sewage sludge-based on microwave pretreatment. Bioresour. Technol..

[21] Schloss P.D., Westcott S.L., Ryabin T., Hall J.R., Hartmann M., Hollister E.B., Lesniewski R.A., Oakley B.B., Parks D.H., Robinson C.J. (2009). Introducing mothur: Open-Source, Platform-Independent, Community-Supported Software for Describing and Comparing Microbial Communities. Appl. Environ. Microbiol..

[22] Quast C., Pruesse E., Yilmaz P., Gerken J., Schweer T., Yarza P., Peplies J., Glöckner F.O. (2013). The SILVA ribosomal RNA gene database project: Improved data processing and web-based tools. Nucleic Acids Res..

[23] Yilmaz P., Parfrey L.W., Yarza P., Gerken J., Pruesse E., Quast C., Schweer T., Peplies J., Ludwig W., Glöckner F.O. (2014). The SILVA and “All-species Living Tree Project (LTP)” taxonomic frameworks. Nucleic Acids Res..

[24] Ter-Braak C.J.F., Šmilauer P. (2002). CANOCO Reference Manual and User’s Guide to Canoco for Windows. Software for Canonical Community Ordination (Version 4.5).

[25] Ferreira A., Nobre M., Moore E., Rainey F.A., Battista J.R., da Costa M.S. (1999). Characterization and radiation resistance of new isolates of *Rubrobacter radiotolerans* and *Rubrobacter xylanophilus*. Extremophiles.

[26] Feng X.M., Mo Y.X., Han L., Nogi Y., Zhu Y.H., Lv J. (2015). *Qipengyuania sediminis*, gen. nov., sp. nov., a member of the family *Erythrobacteraceae* isolated from subterrestrial sediment. Int. J. Syst. Evol. Microbiol..

[27] Saiz-Jimenez C., Laiz L. (2000). Occurrence of halotolerant/halophilic bacterial communities in deteriorated monuments. Int. Biodeterior. Biodegrad..

[28] Piñar G., Ripka K., Weber J., Sterflinger K. (2009). The microbiota of a sub-surface monument the medieval chapel of St. Virgil (Vienna, Austria). Int. Biodeterior. Biodegrad..

[29] Piñar G., Piombino-Mascali D., Maixner F., Zink A., Sterflinger K. (2013). Microbial survey of the mummies from the Capuchin Catacombs of Palermo. Italy: Biodeterioration risk and contamination of the indoor air. FEMS Microbiol. Ecol..

[30] Liu X., Meng H., Wang Y., Katayama Y., Gu J.-D. (2018). Water is a critical factor in evaluating and assessing microbial colonization and destruction of Angkor sandstone monuments. Int. Biodeterior. Biodegrad..

[31] Ortega-Morales B., Narváez-Zapata J., Schmalenberger A., Sosa-López A., Tebbe C. (2004). Biofilms fouling ancient limestone Mayan monuments in Uxmal, Mexico: A cultivation-independent analysis. Biofilms.

[32] Jing L., Maocheng D., Gao L., Yen S., Katayama Y., Gu J. (2021). The active microbes and biochemical processes contributing to deterioration of Angkor sandstone monuments under the tropical climate in Cambodia—A review. J. Cult. Herit..

[33] Sterflinger K., Piñar G. (2013). Microbial deterioration of cultural heritage and works of art—tilting at windmills?. Appl. Microbiol. Biotechnol..

[34] Piñar G., Dalnodar D., Voitl C., Reschreiter H., Sterflinger K. (2016). Biodeterioration Risk Threatens the 3100-Year-Old Staircase of Hallstatt (Austria): Possible Involvement of Halophilic Microorganisms. PLoS ONE.

[35] Ettenauer J.D., Jurado V., Piñar G., Miller A.Z., Santner M., Saiz-Jimenez C., Sterflinger K. (2014). Halophilic Microorganisms Are Responsible for the Rosy Discolouration of Saline Environments in Three Historical Buildings with Mural Paintings. PLoS ONE.

[36] Piñar G., Ramos C., Rolleke S., Schabereiter-Gurtner C., Vybiral D., Lubitz W., Denner E.B. (2001). Detection of indigenous *Halobacillus* populations in damaged ancient wall paintings and building materials: Molecular monitoring and cultivation. Appl. Environ. Microbiol..

[37] Piñar G., Ettenauer J., Sterflinger K., Saiz-Jimenez C. (2014). La vie en rose: A review of the rosy discoloration of subsurface monuments. The Conservation of Subterranean Cultural Heritage.

[38] Oren A. (2009). Microbial diversity and microbial abundance in salt-saturated brines: Why are the waters of hypersaline lakes red?. Nat. Resour. Environ. Issues.

[39] Wuchter C., Abbas B., Coolen M.J.L., Herfort L., van Bleijswijk J., Timmers P., Strous M., Teira E., Herndl G.J., Middelburg J.J. (2006). Archaeal nitrification in the ocean. Proc. Natl. Acad. Sci. USA.

[40] Leininger S., Urich T., Schloter M., Schwark L., Qi J., Nicol G.W., Prosser J.I., Schuster S.C., Schleper C. (2006). Archaea predominate among ammonia-oxidizing prokaryotes in soils. Nature.

[41] Stopnisek N., Gubry-Rangin C., Höfferle S., Nicol G.W., Mandic-Mulec I., Prosser J.I. (2010). *Thaumarchaeal* ammonia oxidation in an acidic forest peat soil is not influenced by ammonium amendment. Appl. Environ. Microbiol..

[42] Yao H., Gao Y., Nicol G.W., Campbell C.D., Prosser J.I., Zhang L., Han W., Singh B.K. (2011). Links between ammonia oxidizer community structure, abundance, and nitrification potential in acidic soils. Appl. Environ. Microbiol..

[43] Zhang L.-M., Hu H.-W., Shen J.-P., He J.-Z. (2012). Ammonia-oxidizing archaea have more important role than ammonia-oxidizing bacteria in ammonia oxidation of strongly acidic soils. ISME J..

[44] Mansch R., Bock E. (1998). Biodeterioration of natural stone with special reference to nitrifying bacteria. Biodeterioration.

[45] Sand W., Bock E. (1991). Biodeterioration of mineral materials by microorganisms—biogenic sulfuric and nitric acid corrosion of concrete and natural stone. Geomicrobiol. J..

[46] Diercks M., Sand W., Bock E. (1991). Microbial corrosion of concrete. Experientia.

[47] Hu H.-W., Xu Z.-H., He J.-Z. (2014). Chapter Six—Ammonia-Oxidizing Archaea Play a Predominant Role in Acid Soil Nitrification. Adv. Agron..

[48] Bai F.-Y., Chen X.-P., Huang J.-Z., Lu Y.-S., Dong H.-Y., Wu Y.-H., Song S.-L., Yu J., Bai S., Chen Z. (2021). Microbial biofilms on a giant monolithic statue of Buddha: The symbiosis of microorganisms and mosses and implications for bioweathering. Int. Biodeterior. Biodegrad..

[49] Meng H., Zhang X., Katayama Y., Ge Q., Gu J.D. (2020). Microbial diversity and composition of the Preah Vihear temple in Cambodia by high-throughput sequencing based on genomic DNA and RNA. Int. Biodeterior. Biodegrad..

[50] Stauch-White K., Srinivasan V.N., Camilla Kuo-Dahab W.C., Park C., Butler C.S. (2017). The role of inorganic nitrogen in successful formation of granular biofilms for wastewater treatment that support cyanobacteria and bacteria. AMB Express.

[51] Trivedi C., Reich P.B., Maestre F.T., Hu H.-W., Singh B.K., Delgado-Baquerizo M. (2019). Plant-driven niche differentiation of ammonia-oxidizing bacteria and archaea in global drylands. ISME J..

[52] Scheerer S., Ortega-Morales O., Gaylarde C. (2009). Microbial deterioration of stone monuments—An updated overview. Adv. Appl. Microbiol..

[53] Laiz L., Miller A.Z., Jurado V., Akatova E., Sanchez-Moral S., Gonzalez J.M., Dionísio A., Macedo M.F., Saiz-Jimenez C. (2009). Isolation of five *Rubrobacter* strains from biodeteriorated monuments. Naturwissenschaften.

[54] Jurado V., Miller A.Z., Alias-Villegas C., Laiz L., Saiz-Jimenez C. (2012). *Rubrobacter bracarensis* sp. nov., a novel member of the genus *Rubrobacter* isolated from a biodeteriorated monument. Syst. Appl. Microbiol..

[55] Jroundi F., Elert K., Ruiz-Agudo E., Gonzalez-Muñoz M.T., Rodriguez-Navarro C. (2020). Bacterial Diversity Evolution in Maya Plaster and Stone Following a Bio-Conservation Treatment. Front. Microbiol..

[56] Schröer L., De Kock T., Cnudde V., Boon N. (2020). Differential colonization of microbial communities inhabiting Lede stone in the urban and rural environment. Sci. Total Environ..

[57] Ortega-Calvo J.J., Hernandez-Marine M., Saiz-Jimenez C. (1991). Biodeterioration of building materials by cyanobacteria and algae. Int. Biodeterior..

[58] Li Q., Zhang B., He Z., Yang X. (2016). Distribution and Diversity of Bacteria and Fungi Colonization in Stone Monuments Analyzed by High-Throughput Sequencing. PLoS ONE.

[59] Zhang X., Ge Q., Zhu Z., Deng Y., Gu J.-D. (2018). Microbiological community of the Royal Palace in Angkor Thom and Beng Mealea of Cambodia by Illumina sequencing based on 16S rRNA gene. Int. Biodeterior. Biodegrad..

[60] Kertesz M.A., Kawasaki A., Timmis K.N. (2010). Hydrocarbon-Degrading *Sphingomonads*: *Sphingomonas*, *Sphingobium*, *Novosphingobium*, and *Sphingopyxis*. Handbook of Hydrocarbon and Lipid Microbiology.

[61] Kwon K.K., Woo J.H., Yang S.H., Kang J.H., Kang S.G., Kim S.G., Sato T., Kato C. (2007). *Altererythrobacter epoxidivorans* gen. nov., sp. nov., an epoxide hydrolase-active, mesophilic marine bacterium isolated from cold-seep sediment, and reclassification of *Erythrobacter luteolus* Yoon et al. 2005 as *Altererythrobacter luteolus* comb. nov. Int. J. Syst. Evol. Microbiol..

[62] Xue X., Zhang K., Cai F., Dai J., Wang Y., Rahman E., Peng F., Fang C. (2012). *Altererythrobacter xinjiangensis* sp. nov., isolated from desert sand, and emended description of the genus *Altererythrobacter*. Int. J. Syst. Evol. Microbiol..

[63] Xue H., Piao C.G., Guo M.W., Wang L.F., Fang W., Li Y. (2016). Description of *Altererythrobacter aerius* sp. nov., isolated from air, and emended description of the genus *Altererythrobacter*. Int. J. Syst. Evol. Microbiol..

[64] Park S.C., Baik K.S., Choe H.N., Lim C.H., Kim H.J., Ka J.O., Seong C.N. (2011). *Altererythrobacter namhicola* sp. nov. and *Altererythrobacter aestuarii* sp. nov., isolated from seawater. Int. J. Syst. Evol. Microbiol..

[65] Meisinger D.B., Zimmermann J., Ludwig W., Schleifer K.-H., Wanner G., Schmid M., Bennett P.C., Engel A.S., Lee N.M. (2007). In situ detection of novel *Acidobacteria* in microbial mats from a chemolithoautotrophically based cave ecosystem (Lower Kane Cave, WY, USA). Environ. Microbiol..

[66] Kämpfer P., Glaeser S.P., Schäfer J., Lodders N., Martin K., Schumann P. (2013). *Ornithinimicrobium murale* sp. nov., isolated from an indoor wall colonized by moulds. Int. J. Syst. Evol. Microbiol..

[67] Feng X.M., Yan D., Bai J.L., Su J., Liu H.Y., Ma B.P., Zhang Y.Q., Yu L.Y. (2017). *Ornithinimicrobium flavum* sp. nov., isolated from the leaf of *Paris polyphylla*. Int. J. Syst. Evol. Microbiol..

[68] Liu L.Z., Liu Y., Chen Z., Liu H.C., Zhou Y.G., Liu Z.P. (2013). *Ornithinimicrobium tianjinense* sp. nov., isolated from a recirculating aquaculture system. Int. J. Syst. Evol. Microbiol..

[69] Park S., Song J., Yoshizawa S., Choi A., Cho J.C., Kogure K. (2013). *Rubrivirga marina* gen. nov., sp. nov., a member of the family *Rhodothermaceae* isolated from deep seawater. Int. J. Syst. Evol. Microbiol..

[70] Song J., Joung Y., Park S., Cho J.C., Kogure K. (2016). *Rubrivirga profundi* sp. nov., isolated from deep-sea water, and emended description of the genus *Rubrivirga*. Int. J. Syst. Evol. Microbiol..

[71] Yoon J., Oku N., Park S., Katsuta A., Kasai H. (2012). *Tunicatimonas pelagia* gen. nov., sp. nov., a novel representative of the family *Flammeovirgaceae* isolated from a sea anemone by the differential growth screening method. Antonie Van Leeuwenhoek.

[72] Dastager S.G., Lee J.C., Ju Y.J., Park D.J., Kim C.J. (2008). *Rubellimicrobium mesophilum* sp. nov., a mesophilic, pigmented bacterium isolated from soil. Int. J. Syst. Evol. Microbiol..

[73] Weon H.Y., Son J.A., Yoo S.H., Hong S.B., Jeon Y.A., Kwon S.W., Koo B.S. (2009). *Rubellimicrobium aerolatum* sp. nov., isolated from an air sample in Korea. Int. J. Syst. Evol. Microbiol..

