# Supplementary material for: Bacterial and Archaeal Structural Diversity in Several Biodeterioration Patterns on the Limestone Walls of the Old Cathedral of Coimbra
Source: Microorganisms. 2021 Mar 30;9(4):709. doi: 10.3390/microorganisms9040709 (PMC8065406; doi:10.3390/microorganisms9040709)
Supplement: Supplementary file 1 [file microorganisms-09-00709-s001.zip › SuplementaryFigure_Sfig1.pptx]

## Slide 1
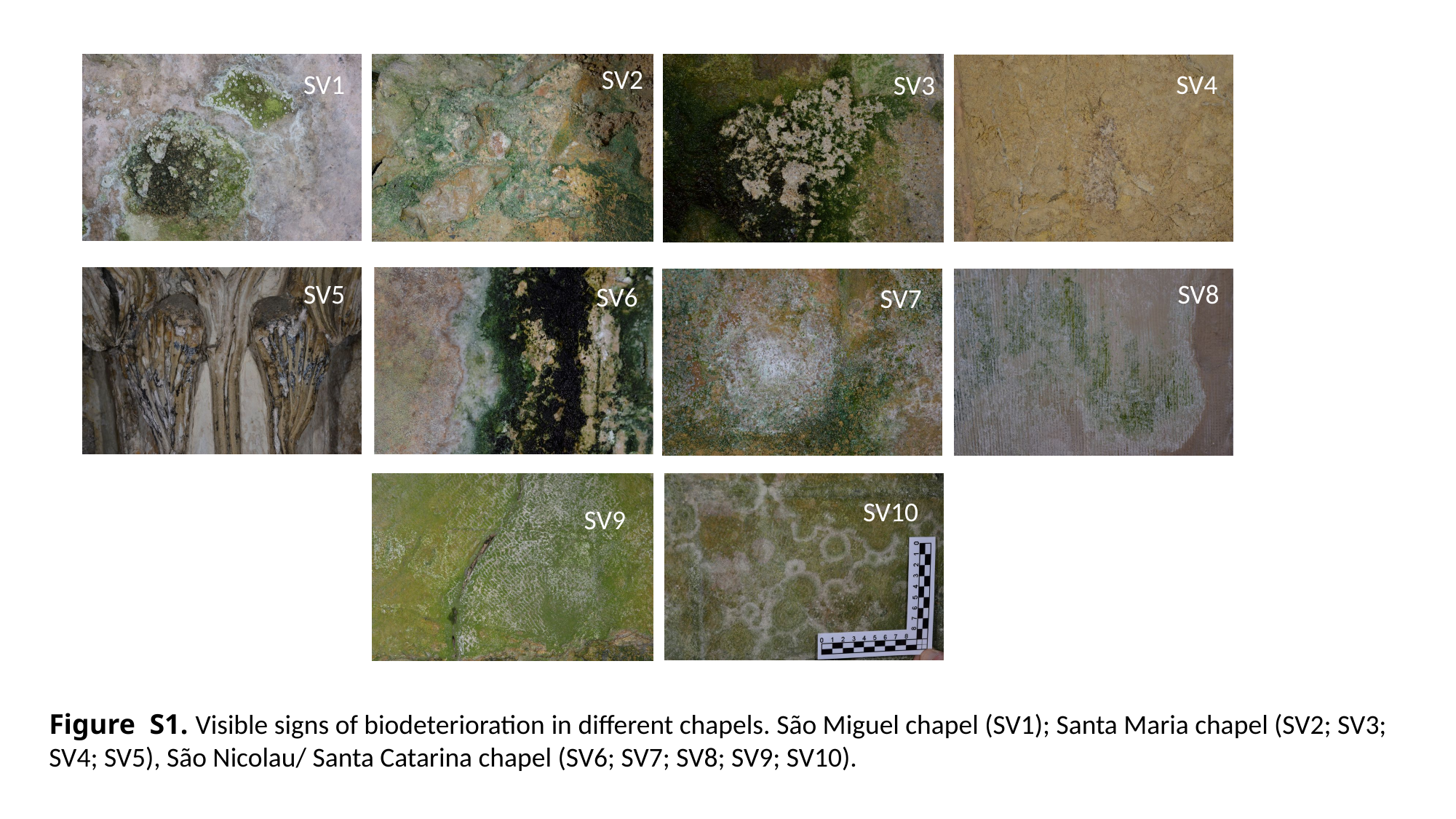

SV2
SV1
SV4
SV3
SV5
SV8
SV6
SV7
SV10
SV9
Figure S1. Visible signs of biodeterioration in different chapels. São Miguel chapel (SV1); Santa Maria chapel (SV2; SV3; SV4; SV5), São Nicolau/ Santa Catarina chapel (SV6; SV7; SV8; SV9; SV10).
