# Supplementary material for: Bacterial and Archaeal Structural Diversity in Several Biodeterioration Patterns on the Limestone Walls of the Old Cathedral of Coimbra
Source: Microorganisms. 2021 Mar 30;9(4):709. doi: 10.3390/microorganisms9040709 (PMC8065406; doi:10.3390/microorganisms9040709)
Supplement: Supplementary file 1 [file microorganisms-09-00709-s001.zip › SupplementaryFiguresArchaea_Sfig3.pptx]

## Slide 1
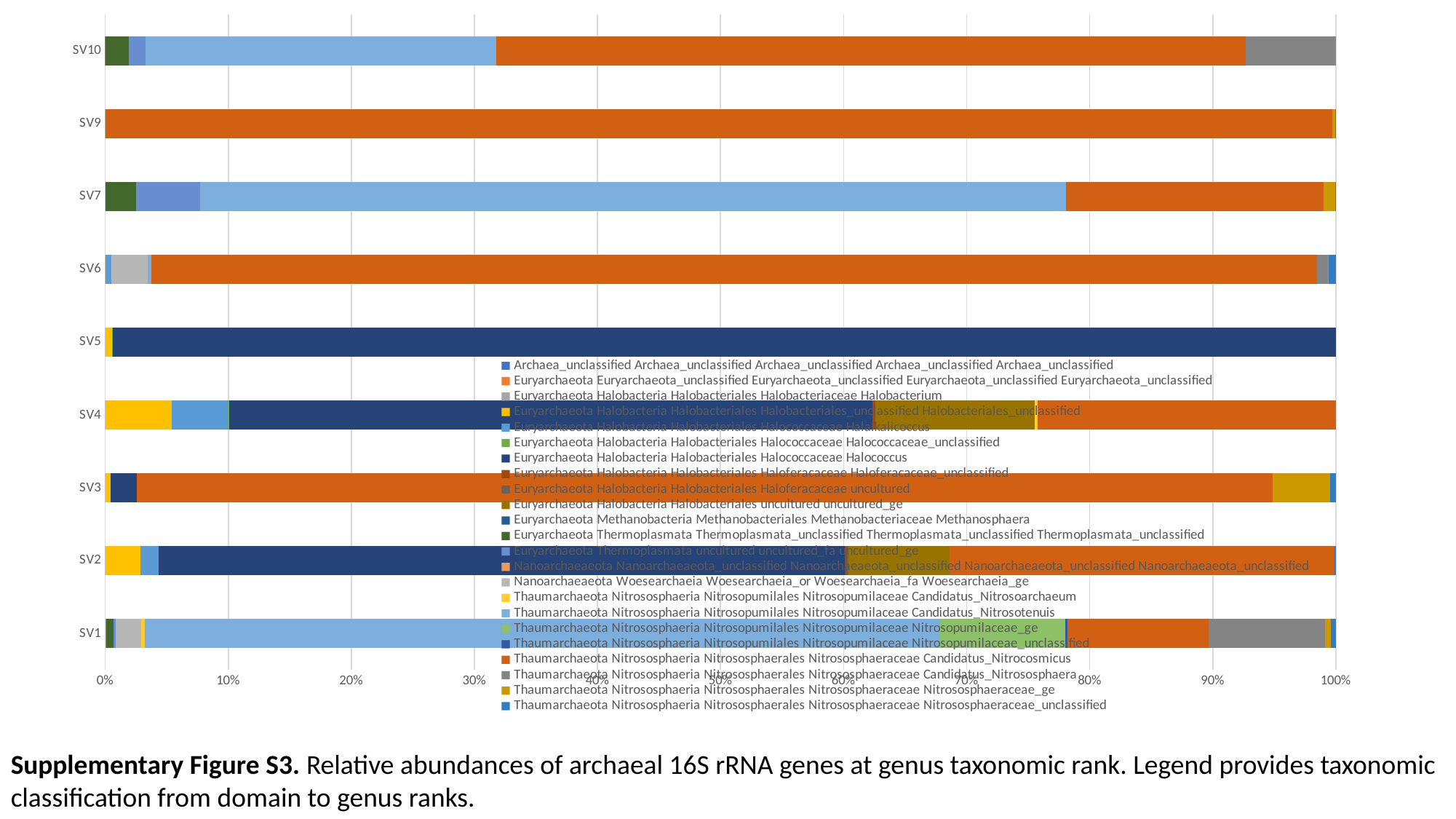

### Chart
| Category | Archaea_unclassified | Euryarchaeota | Euryarchaeota | Euryarchaeota | Euryarchaeota | Euryarchaeota | Euryarchaeota | Euryarchaeota | Euryarchaeota | Euryarchaeota | Euryarchaeota | Euryarchaeota | Euryarchaeota | Nanoarchaeaeota | Nanoarchaeaeota | Thaumarchaeota | Thaumarchaeota | Thaumarchaeota | Thaumarchaeota | Thaumarchaeota | Thaumarchaeota | Thaumarchaeota | Thaumarchaeota |
|---|---|---|---|---|---|---|---|---|---|---|---|---|---|---|---|---|---|---|---|---|---|---|---|
| SV1 | 0.03390405153415833 | 0.04520540204554444 | 0.0 | 0.0 | 0.0 | 0.0 | 0.0 | 0.0 | 0.0 | 0.0 | 0.0 | 0.61027292761485 | 0.15821890715940554 | 0.02260270102277222 | 2.0342430920495 | 0.305136463807425 | 64.64937560038425 | 10.137311408713341 | 0.22037633497202916 | 11.459569418545517 | 9.425326326496016 | 0.5142114482680681 | 0.38424591738712777 |
| SV2 | 0.0 | 0.0 | 0.0 | 2.830188679245283 | 1.509433962264151 | 0.0 | 55.75471698113208 | 0.18867924528301888 | 0.09433962264150944 | 8.20754716981132 | 0.0 | 0.0 | 0.0 | 0.0 | 0.0 | 0.0 | 0.0 | 0.0 | 0.0 | 31.32075471698113 | 0.0 | 0.0 | 0.09433962264150944 |
| SV3 | 0.0 | 0.0 | 0.0 | 0.4189108318372232 | 0.0 | 0.0 | 2.154398563734291 | 0.0 | 0.0 | 0.0 | 0.0 | 0.0 | 0.0 | 0.0 | 0.0 | 0.0 | 0.0 | 0.0 | 0.0 | 92.28007181328546 | 0.0 | 4.667863554757631 | 0.47875523638539796 |
| SV4 | 0.0 | 0.0 | 0.0 | 5.423406279733587 | 4.567078972407232 | 0.09514747859181731 | 52.23596574690771 | 0.285442435775452 | 0.0 | 12.940057088487155 | 0.0 | 0.0 | 0.0 | 0.0 | 0.0 | 0.19029495718363462 | 0.0 | 0.0 | 0.0 | 24.262607040913416 | 0.0 | 0.0 | 0.0 |
| SV5 | 0.0 | 0.0 | 0.01382093595378279 | 0.5307239406252592 | 0.03593443347983526 | 0.0331702462890787 | 99.38635044365205 | 0.0 | 0.0 | 0.0 | 0.0 | 0.0 | 0.0 | 0.0 | 0.0 | 0.0 | 0.0 | 0.0 | 0.0 | 0.0 | 0.0 | 0.0 | 0.0 |
| SV6 | 0.0 | 0.0 | 0.0 | 0.0 | 0.47189597315436244 | 0.0 | 0.0 | 0.0 | 0.0 | 0.0 | 0.0 | 0.0 | 0.0 | 0.02097315436241611 | 2.936241610738255 | 0.0 | 0.33557046979865773 | 0.0 | 0.0 | 94.68330536912751 | 0.9123322147651006 | 0.04194630872483222 | 0.597734899328859 |
| SV7 | 0.0 | 0.01307787876806382 | 0.0 | 0.0 | 0.01307787876806382 | 0.0 | 0.0 | 0.0 | 0.0 | 0.0 | 0.01961681815209573 | 2.478258026548094 | 5.198456810305369 | 0.0 | 0.0 | 0.0 | 70.36552671156738 | 0.0 | 0.0 | 20.904989210750017 | 0.00653893938403191 | 0.9481462106846269 | 0.05231151507225528 |
| SV9 | 0.0 | 0.0 | 0.0 | 0.0 | 0.0 | 0.0 | 0.008241984669908513 | 0.0 | 0.0 | 0.0 | 0.0 | 0.0 | 0.0 | 0.0 | 0.0 | 0.0 | 0.0 | 0.0 | 0.0 | 99.72801450589301 | 0.0 | 0.18956564740789583 | 0.07417786202917663 |
| SV10 | 0.0 | 0.0 | 0.0 | 0.0 | 0.0 | 0.0 | 0.0 | 0.0 | 0.0 | 0.0 | 0.0 | 1.9021739130434783 | 1.358695652173913 | 0.0 | 0.0 | 0.0 | 28.532608695652176 | 0.0 | 0.0 | 60.869565217391305 | 7.336956521739131 | 0.0 | 0.0 |Supplementary Figure S3. Relative abundances of archaeal 16S rRNA genes at genus taxonomic rank. Legend provides taxonomic classification from domain to genus ranks.
