# Supplementary material for: Bacterial and Archaeal Structural Diversity in Several Biodeterioration Patterns on the Limestone Walls of the Old Cathedral of Coimbra
Source: Microorganisms. 2021 Mar 30;9(4):709. doi: 10.3390/microorganisms9040709 (PMC8065406; doi:10.3390/microorganisms9040709)
Supplement: Supplementary file 1 [file microorganisms-09-00709-s001.zip › SupplementaryFiguresBacteria_SFig2.pptx]

## Slide 1
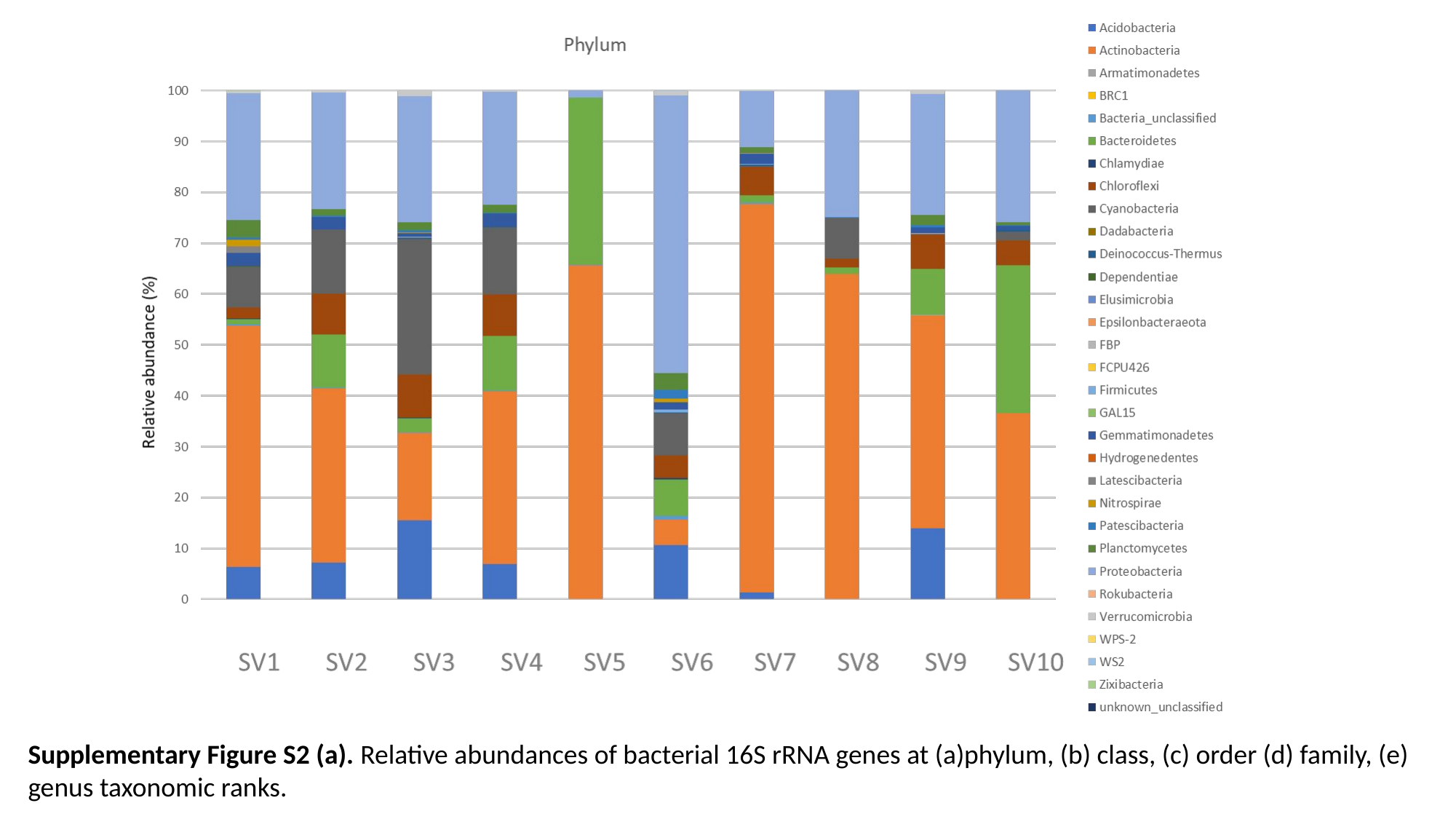

Supplementary Figure S2 (a). Relative abundances of bacterial 16S rRNA genes at (a)phylum, (b) class, (c) order (d) family, (e) genus taxonomic ranks.

## Slide 2
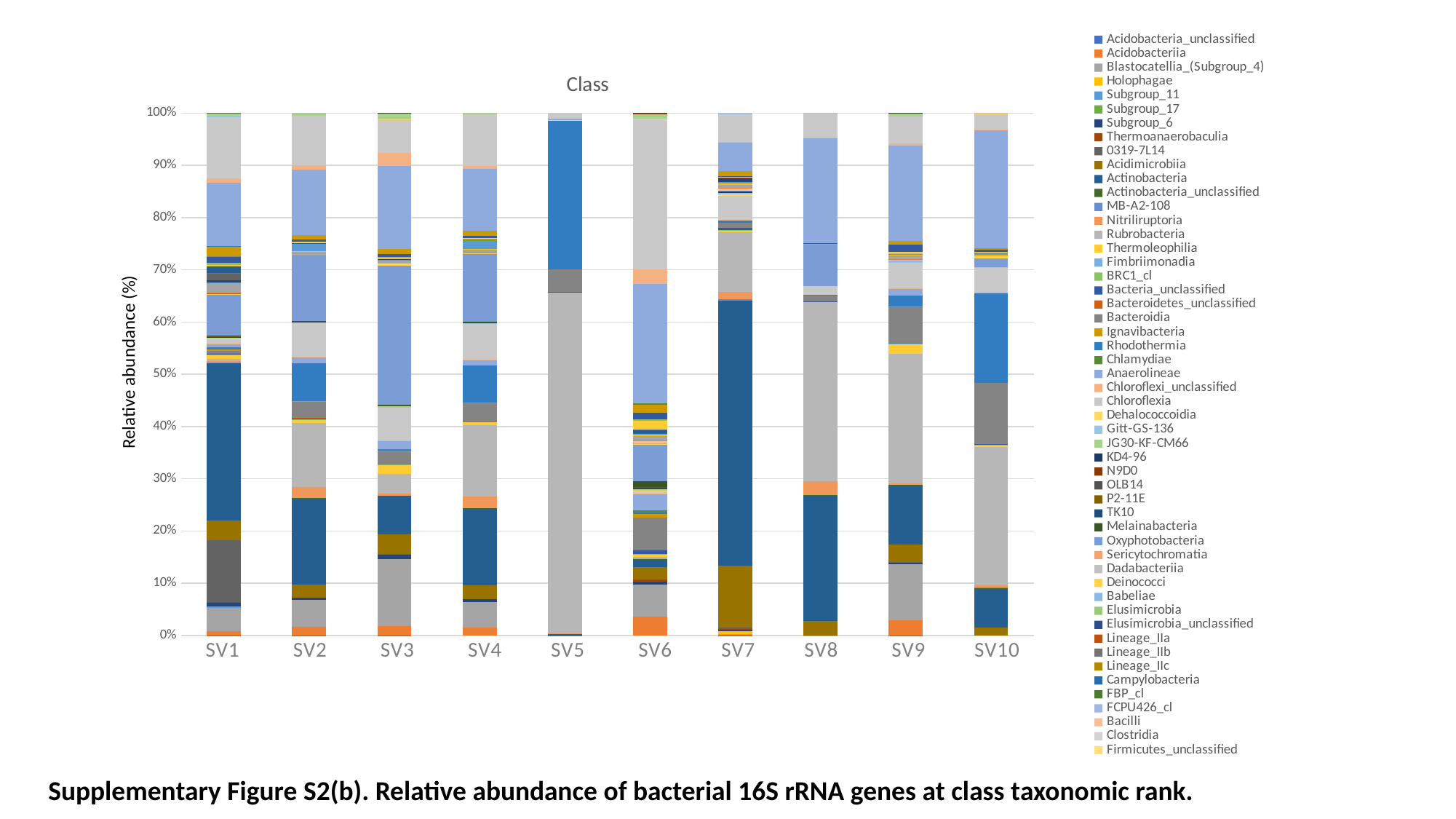

### Chart: Class
| Category | Acidobacteria_unclassified | Acidobacteriia | Blastocatellia_(Subgroup_4) | Holophagae | Subgroup_11 | Subgroup_17 | Subgroup_6 | Thermoanaerobaculia | 0319-7L14 | Acidimicrobiia | Actinobacteria | Actinobacteria_unclassified | MB-A2-108 | Nitriliruptoria | Rubrobacteria | Thermoleophilia | Fimbriimonadia | BRC1_cl | Bacteria_unclassified | Bacteroidetes_unclassified | Bacteroidia | Ignavibacteria | Rhodothermia | Chlamydiae | Anaerolineae | Chloroflexi_unclassified | Chloroflexia | Dehalococcoidia | Gitt-GS-136 | JG30-KF-CM66 | KD4-96 | N9D0 | OLB14 | P2-11E | TK10 | Melainabacteria | Oxyphotobacteria | Sericytochromatia | Dadabacteriia | Deinococci | Babeliae | Elusimicrobia | Elusimicrobia_unclassified | Lineage_IIa | Lineage_IIb | Lineage_IIc | Campylobacteria | FBP_cl | FCPU426_cl | Bacilli | Clostridia | Firmicutes_unclassified | Limnochordia | GAL15_cl | AKAU4049 | BD2-11_terrestrial_group | Gemmatimonadetes | Gemmatimonadetes_unclassified | Longimicrobia | PAUC43f_marine_benthic_group | S0134_terrestrial_group | Hydrogenedentia | Latescibacteria | Latescibacteria_cl | Nitrospira | ABY1 | Gracilibacteria | Microgenomatia | Parcubacteria | Saccharimonadia | BD7-11 | OM190 | Phycisphaerae | Pla3_lineage | Pla4_lineage | Planctomycetacia | Planctomycetes_unclassified | vadinHA49 | Alphaproteobacteria | Deltaproteobacteria | Gammaproteobacteria | Proteobacteria_unclassified | NC10 | Verrucomicrobiae | WPS-2_cl | WS2_cl | Zixibacteria_cl | unknown_unclassified |
|---|---|---|---|---|---|---|---|---|---|---|---|---|---|---|---|---|---|---|---|---|---|---|---|---|---|---|---|---|---|---|---|---|---|---|---|---|---|---|---|---|---|---|---|---|---|---|---|---|---|---|---|---|---|---|---|---|---|---|---|---|---|---|---|---|---|---|---|---|---|---|---|---|---|---|---|---|---|---|---|---|---|---|---|---|---|---|---|---|
| Sample_2.1 | 0.0196183342250758 | 0.80613518815766 | 4.535402175851614 | 0.0142678794364188 | 0.00535045478865704 | 0.148029249152845 | 0.654538969145711 | 0.14446227929374 | 11.88336008560728 | 3.77207062600321 | 30.18013197788479 | 0.112359550561798 | 0.0160513643659711 | 0.201533797039415 | 0.422685928303906 | 0.823970037453184 | 0.00356696985910469 | 0.00535045478865704 | 0.283574103798823 | 0.0107009095773141 | 0.492241840556447 | 0.278223649010166 | 0.297841983235242 | 0.0713393971820938 | 0.613518815766007 | 0.0214018191546281 | 0.995184590690209 | 0.0659889423934368 | 0.051721062957018 | 0.098091671125379 | 0.169431068307473 | 0.0 | 0.128410914927769 | 0.00535045478865704 | 0.00891742464776173 | 0.0 | 7.970394150169431 | 0.0 | 0.00535045478865704 | 0.0802568218298555 | 0.0535045478865704 | 0.00713393971820938 | 0.00356696985910469 | 0.00891742464776173 | 0.00535045478865704 | 0.00891742464776173 | 0.0 | 0.0 | 0.0 | 0.0196183342250758 | 0.00535045478865704 | 0.0 | 0.0 | 0.0124843945068664 | 0.00891742464776173 | 0.051721062957018 | 1.970750847155341 | 0.0160513643659711 | 0.0428036383092563 | 0.0 | 0.422685928303906 | 0.0 | 1.153914749420367 | 0.171214553237025 | 1.342964151952916 | 0.00535045478865704 | 0.0 | 0.00356696985910469 | 0.0856072766185126 | 0.287141073657928 | 0.00713393971820938 | 0.224719101123596 | 1.282325664348136 | 0.0267522739432852 | 0.0303192438023899 | 1.812020688425183 | 0.057071517745675 | 0.0 | 12.21508828250401 | 0.831103977171393 | 11.80488674870698 | 0.00713393971820938 | 0.0428036383092563 | 0.501159265204209 | 0.0 | 0.0 | 0.0731228821116462 | 0.00713393971820938 |
| Sample_3.1 | 0.0094509025611946 | 1.620829789244873 | 5.150741895851054 | 0.0 | 0.0 | 0.0 | 0.392212456289576 | 0.0 | 0.0 | 2.603723655609111 | 16.4303941026368 | 0.0614308666477649 | 0.0 | 2.117002173707589 | 12.27199697571118 | 0.732444948492581 | 0.0 | 0.0141763538417919 | 0.189018051223892 | 0.0047254512805973 | 3.184954163122579 | 0.0330781589641811 | 7.29137132596163 | 0.0 | 1.181362820149324 | 0.0047254512805973 | 6.60618089027502 | 0.0 | 0.0094509025611946 | 0.103959928173141 | 0.0 | 0.0 | 0.0330781589641811 | 0.0 | 0.0047254512805973 | 0.108685379453738 | 12.47519138077686 | 0.0 | 0.0 | 0.0378036102447784 | 0.0094509025611946 | 0.0 | 0.0 | 0.0189018051223892 | 0.0 | 0.0 | 0.0 | 0.0 | 0.0 | 0.0189018051223892 | 0.0094509025611946 | 0.0 | 0.0 | 0.0 | 0.0 | 0.0661563179283622 | 0.600132312635857 | 0.207919856346281 | 1.403459030337397 | 0.0 | 0.0661563179283622 | 0.0 | 0.0 | 0.0 | 0.0330781589641811 | 0.0 | 0.0 | 0.0 | 0.0 | 0.283527076835838 | 0.0 | 0.0047254512805973 | 0.425290615253757 | 0.0 | 0.0 | 0.845855779226916 | 0.0047254512805973 | 0.0 | 12.47991683205746 | 0.841130327946319 | 9.540686135525945 | 0.0 | 0.0 | 0.467819676779132 | 0.0 | 0.0 | 0.0 | 0.0 |
| Sample_3.2 | 0.0130639261452709 | 1.770161992684202 | 12.74603727573594 | 0.0 | 0.0 | 0.0 | 1.016808918306915 | 0.0 | 0.0239505312663299 | 3.816843755443302 | 7.376763630029612 | 0.0174185681936945 | 0.0 | 0.302647622365442 | 3.866922139000173 | 1.778871276781049 | 0.0 | 0.0152412471694827 | 0.124107298380073 | 0.00217732102421181 | 2.386343842536144 | 0.0522557045810834 | 0.335307437728619 | 0.00870928409684724 | 1.567671137432503 | 0.0870928409684724 | 6.39914649015851 | 0.0 | 0.0239505312663299 | 0.261278522905417 | 0.00435464204842362 | 0.0 | 0.00870928409684724 | 0.0 | 0.108866051210591 | 0.010886605121059 | 26.6221041630378 | 0.00435464204842362 | 0.0 | 0.304824943389654 | 0.0 | 0.0 | 0.0 | 0.0892701619926842 | 0.0 | 0.0 | 0.0 | 0.0239505312663299 | 0.0 | 0.0 | 0.0 | 0.00217732102421181 | 0.0 | 0.0 | 0.0 | 0.0 | 0.581344713464553 | 0.0 | 0.158944434767462 | 0.0 | 0.080560877895837 | 0.0 | 0.0 | 0.0 | 0.137171224525344 | 0.0 | 0.0 | 0.0 | 0.00653196307263543 | 0.363612611043372 | 0.0 | 0.00870928409684724 | 0.52908900888347 | 0.0 | 0.0 | 0.99939035011322 | 0.0 | 0.0 | 15.8552516983104 | 2.445131510189863 | 6.490593973175405 | 0.034837136387389 | 0.0 | 1.125674969517506 | 0.010886605121059 | 0.0 | 0.0 | 0.0 |
| Sample_3.3 | 0.0 | 1.556381162767346 | 4.854905110954914 | 0.0 | 0.0 | 0.0 | 0.497037855206346 | 0.0 | 0.0 | 2.640827392308465 | 14.81072396826991 | 0.0652675971483081 | 0.0 | 2.219098303042474 | 13.56059845366001 | 0.617531880710915 | 0.0 | 0.0050205843960237 | 0.155638116276735 | 0.0050205843960237 | 3.529470830404659 | 0.0301235063761422 | 7.174415101917862 | 0.0 | 1.00411687920474 | 0.0251029219801185 | 6.99367406366101 | 0.0 | 0.050205843960237 | 0.0803293503363792 | 0.0100411687920474 | 0.0050205843960237 | 0.0150617531880711 | 0.0 | 0.0150617531880711 | 0.105432272316498 | 13.1137664424139 | 0.0 | 0.0 | 0.0803293503363792 | 0.0301235063761422 | 0.0 | 0.0 | 0.0251029219801185 | 0.0 | 0.0 | 0.0 | 0.0 | 0.0 | 0.0150617531880711 | 0.0150617531880711 | 0.0 | 0.0 | 0.0 | 0.0 | 0.0301235063761422 | 0.587408374334773 | 0.125514609900592 | 1.611607591123607 | 0.0150617531880711 | 0.0953911035244502 | 0.0 | 0.0 | 0.0 | 0.0100411687920474 | 0.0 | 0.0 | 0.0 | 0.0 | 0.256049804197209 | 0.0 | 0.0 | 0.517120192790441 | 0.0 | 0.0 | 0.938849282056432 | 0.0 | 0.0 | 11.77327040867557 | 0.657696555879104 | 9.810221909830302 | 0.0 | 0.0 | 0.266090972989256 | 0.0 | 0.0 | 0.0 | 0.0 |
| Sample_3.6 | 0.0 | 0.0 | 0.0 | 0.0 | 0.0 | 0.0 | 0.0 | 0.0 | 0.0 | 0.00542829225925524 | 0.105851699055477 | 0.127564868092498 | 0.0 | 0.119422429703615 | 65.32678319400715 | 0.00271414612962762 | 0.0 | 0.0 | 0.0814243838888286 | 0.00271414612962762 | 4.304635761589403 | 0.0 | 28.53110411464554 | 0.0 | 0.0 | 0.0 | 0.0 | 0.0 | 0.0 | 0.0 | 0.0 | 0.0 | 0.0 | 0.0 | 0.0 | 0.0 | 0.0108565845185105 | 0.0 | 0.0 | 0.0 | 0.0 | 0.0 | 0.0 | 0.0 | 0.0 | 0.0 | 0.00542829225925524 | 0.0 | 0.0 | 0.05699706872218 | 0.021713169037021 | 0.0 | 0.0 | 0.0 | 0.0 | 0.0 | 0.0 | 0.0 | 0.0 | 0.0 | 0.0 | 0.0 | 0.0 | 0.0 | 0.0 | 0.0 | 0.0 | 0.0 | 0.0 | 0.0 | 0.0 | 0.0 | 0.0 | 0.0 | 0.0 | 0.0 | 0.0 | 0.0 | 0.17099120616654 | 0.0 | 1.126370643795462 | 0.0 | 0.0 | 0.0 | 0.0 | 0.0 | 0.0 | 0.0 |
| Sample_4.2 | 0.0 | 3.554237098399194 | 6.190529497369305 | 0.0 | 0.0 | 0.0 | 0.503750139930595 | 0.391805664390462 | 0.0 | 2.496361804544945 | 1.511250419791784 | 0.0111944475540132 | 0.0 | 0.0615694615470727 | 0.291055636404343 | 0.598902944139707 | 0.0 | 0.00559722377700661 | 0.828389118996977 | 0.00559722377700661 | 6.039404455390128 | 0.822791895219971 | 0.190305608418225 | 0.380611216836449 | 3.218403671778798 | 0.00559722377700661 | 0.5933057203627 | 0.027986118885033 | 0.106347251763125 | 0.212694503526251 | 0.123138923094145 | 0.0 | 0.173513937087205 | 0.0 | 0.0 | 1.1866114407254 | 7.10287697302138 | 0.0 | 0.0 | 0.027986118885033 | 0.0447777902160528 | 0.0 | 0.0 | 0.0 | 0.0 | 0.0 | 0.0 | 0.0 | 0.0111944475540132 | 0.55972237770066 | 0.0167916713310198 | 0.0 | 0.0 | 0.0 | 0.0 | 0.0223888951080264 | 0.867569685436024 | 0.190305608418225 | 0.31344453151237 | 0.0 | 0.0503750139930594 | 0.0167916713310198 | 0.0 | 0.0 | 0.64368073435576 | 0.0223888951080264 | 0.0111944475540132 | 0.0 | 0.0895555804321057 | 1.600806000223889 | 0.0 | 0.184708384641218 | 1.332139258927572 | 0.0 | 0.0 | 1.53363931489981 | 0.00559722377700661 | 0.229486174857271 | 22.94861748572707 | 2.664278517855144 | 28.92645247957013 | 0.0223888951080264 | 0.0 | 0.867569685436024 | 0.0 | 0.162319489533192 | 0.0 | 0.0 |
| Sample_4.5 | 0.0147790531553278 | 0.187201339967486 | 0.093600669983743 | 0.507414158332923 | 0.0 | 0.0 | 0.443371594659836 | 0.0443371594659835 | 0.241391201537022 | 11.80353712005518 | 50.6773732696192 | 0.137937829449727 | 0.147790531553278 | 1.517316123946993 | 11.31582836592936 | 0.527119562540027 | 0.0 | 0.0147790531553278 | 0.295581063106557 | 0.0 | 1.073944529287157 | 0.0 | 0.315286467313661 | 0.0 | 0.251243903640573 | 0.0246317552588798 | 4.527316616582095 | 0.221685797329918 | 0.408887137297404 | 0.113306074190847 | 0.0197054042071038 | 0.0 | 0.0394108084142076 | 0.0 | 0.0147790531553278 | 0.0 | 0.187201339967486 | 0.0 | 0.0 | 0.246317552588797 | 0.0344844573624316 | 0.0 | 0.0 | 0.0 | 0.0 | 0.0 | 0.0 | 0.0 | 0.0 | 0.0197054042071038 | 0.0344844573624316 | 0.00492635105177595 | 0.0197054042071038 | 0.0 | 0.0 | 0.226612148381694 | 0.448297945711611 | 0.300507414158333 | 0.251243903640573 | 0.0 | 0.857185083009015 | 0.00492635105177595 | 0.0 | 0.0 | 0.162569584708606 | 0.0 | 0.0 | 0.0 | 0.0 | 0.0098527021035519 | 0.0 | 0.0738952657766392 | 0.093600669983743 | 0.0 | 0.0 | 0.911374944578551 | 0.0 | 0.0 | 5.532292231144392 | 0.0738952657766392 | 5.379575348539337 | 0.0147790531553278 | 0.0246317552588798 | 0.108379723139071 | 0.0 | 0.0 | 0.0 | 0.0 |
| Sample_4.6 | 0.0 | 0.0 | 0.0117633219621221 | 0.0 | 0.0 | 0.0 | 0.0 | 0.0 | 0.0 | 2.678116300043133 | 24.17754773948163 | 0.0627377171313179 | 0.0 | 2.536956436497666 | 34.4312433831314 | 0.0 | 0.0 | 0.0313688585656589 | 0.0313688585656589 | 0.0 | 1.278280986550602 | 0.0 | 0.0352899658863663 | 0.0 | 0.0 | 0.0 | 1.670391718621339 | 0.0 | 0.0 | 0.0 | 0.0 | 0.0 | 0.0 | 0.0 | 0.0 | 0.0 | 7.983374504960201 | 0.0 | 0.0 | 0.0117633219621221 | 0.0 | 0.0 | 0.0 | 0.0 | 0.0 | 0.0 | 0.0 | 0.0 | 0.0 | 0.0274477512449516 | 0.00784221464141474 | 0.0 | 0.0 | 0.0 | 0.0 | 0.0 | 0.0117633219621221 | 0.0 | 0.0 | 0.0 | 0.0 | 0.0 | 0.0 | 0.0 | 0.0 | 0.0 | 0.0 | 0.0 | 0.0 | 0.0588166098106105 | 0.0 | 0.0 | 0.0235266439242442 | 0.0 | 0.0 | 0.0 | 0.0 | 0.0 | 20.26820374073638 | 0.0196055366035368 | 4.630827745755401 | 0.0 | 0.0 | 0.0117633219621221 | 0.0 | 0.0 | 0.0 | 0.0 |
| Sample_4.7 | 0.00336496399488525 | 2.856854431657581 | 10.74096507167373 | 0.0 | 0.0 | 0.0 | 0.407160643381116 | 0.0 | 0.0807591358772462 | 3.311124570967091 | 11.42068779864055 | 0.0302846759539673 | 0.0 | 0.235547479641968 | 24.81660946227876 | 1.907934585099939 | 0.0504744599232788 | 0.0 | 0.121138703815869 | 0.00336496399488525 | 6.989030217376674 | 0.00672992798977051 | 2.025708324920923 | 0.0 | 1.500773941718824 | 0.0201897839693115 | 4.926307288512013 | 0.0100948919846558 | 0.0773941718823609 | 0.164883235749378 | 0.0 | 0.0 | 0.0 | 0.0 | 0.013459855979541 | 0.0 | 0.0639343159028198 | 0.00336496399488525 | 0.0 | 0.0908540278619019 | 0.0 | 0.0 | 0.0 | 0.0 | 0.0 | 0.0 | 0.0 | 0.0605693519079346 | 0.0 | 0.0168248199744263 | 0.013459855979541 | 0.0 | 0.0 | 0.0 | 0.0 | 0.00672992798977051 | 0.864795746685511 | 0.0336496399488525 | 0.215357695672656 | 0.0 | 0.0235547479641968 | 0.0 | 0.0 | 0.0 | 0.0302846759539673 | 0.0 | 0.0 | 0.0 | 0.026919711959082 | 0.296116831549902 | 0.0 | 0.0 | 1.295511138030823 | 0.0 | 0.0 | 0.790766538798035 | 0.0 | 0.0 | 18.20109024833434 | 0.403795679386231 | 5.155124840164208 | 0.0 | 0.0 | 0.679722726966822 | 0.00672992798977051 | 0.0 | 0.0 | 0.0 |
| Sample_4.8 | 0.0 | 0.0 | 0.0 | 0.0 | 0.0 | 0.0 | 0.0 | 0.0 | 0.0 | 1.459457624817568 | 7.589179649051353 | 0.0441231374944846 | 0.00678817499915148 | 0.495536774938058 | 26.53158198418355 | 0.41407867494824 | 0.0 | 0.013576349998303 | 0.0610935749923633 | 0.0 | 11.68923734853885 | 0.0 | 17.34039303533245 | 0.0 | 0.0101822624987272 | 0.0 | 4.78905746190137 | 0.00678817499915148 | 0.00678817499915148 | 0.00678817499915148 | 0.0 | 0.0 | 0.0 | 0.0 | 0.0 | 0.0 | 1.771713674778536 | 0.0 | 0.0 | 0.458201812442725 | 0.0101822624987272 | 0.0 | 0.0 | 0.0 | 0.0 | 0.0 | 0.0 | 0.0 | 0.0 | 0.0 | 0.0169704374978787 | 0.0 | 0.0 | 0.0 | 0.0 | 0.00678817499915148 | 0.0373349624953331 | 0.13576349998303 | 0.556630349930421 | 0.0 | 0.0 | 0.0 | 0.0 | 0.0 | 0.0 | 0.0 | 0.0 | 0.0 | 0.013576349998303 | 0.013576349998303 | 0.0 | 0.0 | 0.298679699962665 | 0.0 | 0.0 | 0.278315174965211 | 0.00339408749957574 | 0.0 | 22.75056850965618 | 0.0237586124970302 | 3.132742762108407 | 0.00339408749957574 | 0.0 | 0.0237586124970302 | 0.0 | 0.0 | 0.0 | 0.0 |Relative abundance (%)
Supplementary Figure S2(b). Relative abundance of bacterial 16S rRNA genes at class taxonomic rank.

## Slide 3
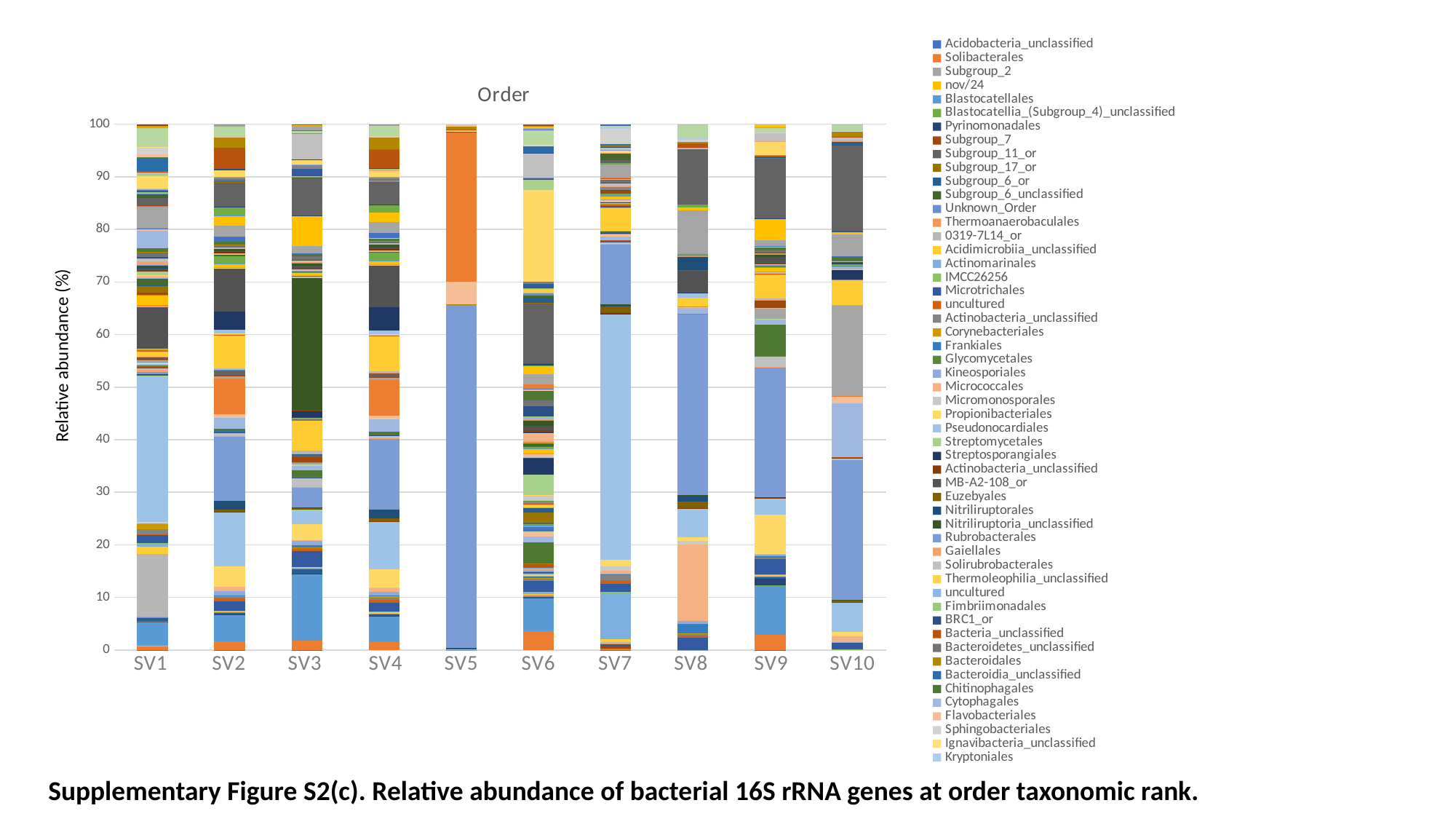

### Chart: Order
| Category | Acidobacteria_unclassified | Solibacterales | Subgroup_2 | nov/24 | Blastocatellales | Blastocatellia_(Subgroup_4)_unclassified | Pyrinomonadales | Subgroup_7 | Subgroup_11_or | Subgroup_17_or | Subgroup_6_or | Subgroup_6_unclassified | Unknown_Order | Thermoanaerobaculales | 0319-7L14_or | Acidimicrobiia_unclassified | Actinomarinales | IMCC26256 | Microtrichales | uncultured | Actinobacteria_unclassified | Corynebacteriales | Frankiales | Glycomycetales | Kineosporiales | Micrococcales | Micromonosporales | Propionibacteriales | Pseudonocardiales | Streptomycetales | Streptosporangiales | Actinobacteria_unclassified | MB-A2-108_or | Euzebyales | Nitriliruptorales | Nitriliruptoria_unclassified | Rubrobacterales | Gaiellales | Solirubrobacterales | Thermoleophilia_unclassified | uncultured | Fimbriimonadales | BRC1_or | Bacteria_unclassified | Bacteroidetes_unclassified | Bacteroidales | Bacteroidia_unclassified | Chitinophagales | Cytophagales | Flavobacteriales | Sphingobacteriales | Ignavibacteria_unclassified | Kryptoniales | OPB56 | SJA-28 | Balneolales | Rhodothermales | Rhodothermia_unclassified | Chlamydiales | Anaerolineae_unclassified | Anaerolineales | Ardenticatenales | Caldilineales | RBG-13-54-9 | SBR1031 | Chloroflexi_unclassified | Chloroflexales | Chloroflexia_unclassified | Kallotenuales | Thermomicrobiales | Dehalococcoidia_unclassified | S085 | SAR202_clade | Gitt-GS-136_or | JG30-KF-CM66_or | KD4-96_or | N9D0_or | OLB14_or | P2-11E_or | TK10_or | Obscuribacterales | Vampirovibrionales | Chloroplast | Leptolyngbyales | Nostocales | Oxyphotobacteria_Incertae_Sedis | Oxyphotobacteria_unclassified | Phormidesmiales | Thermosynechococcales | uncultured | Sericytochromatia_or | Dadabacteriales | Deinococcales | Deinococci_unclassified | Babeliales | Lineage_IV | Elusimicrobia_unclassified | Lineage_IIa_or | Lineage_IIb_or | Lineage_IIc_or | Campylobacterales | FBP_or | FCPU426_or | Bacillales | Lactobacillales | Clostridiales | Firmicutes_unclassified | Limnochordales | GAL15_or | AKAU4049_or | BD2-11_terrestrial_group_or | Gemmatimonadales | Gemmatimonadetes_unclassified | Longimicrobiales | PAUC43f_marine_benthic_group_or | S0134_terrestrial_group_or | Hydrogenedentiales | Latescibacterales | Latescibacteria_or | Nitrospirales | Candidatus_Magasanikbacteria | Gracilibacteria_or | Candidatus_Pacebacteria | Candidatus_Campbellbacteria | Candidatus_Jorgensenbacteria | Candidatus_Kaiserbacteria | Candidatus_Liptonbacteria | Candidatus_Nomurabacteria | Candidatus_Yanofskybacteria | Candidatus_Zambryskibacteria | GWA2-38-13b | Parcubacteria_or | Parcubacteria_unclassified | Saccharimonadales | BD7-11_or | OM190_or | C86 | CCM11a | Phycisphaerae_unclassified | Phycisphaerales | Tepidisphaerales | Pla3_lineage_or | Pla4_lineage_or | Gemmatales | Isosphaerales | Pirellulales | Planctomycetales | uncultured | Planctomycetes_unclassified | vadinHA49_or | Acetobacterales | Alphaproteobacteria_Incertae_Sedis | Alphaproteobacteria_unclassified | Azospirillales | Caedibacterales | Caulobacterales | Dongiales | Elsterales | Holosporales | Micavibrionales | Paracaedibacterales | Parvibaculales | Puniceispirillales | Reyranellales | Rhizobiales | Rhodobacterales | Rhodospirillales | Rhodovibrionales | Rickettsiales | Sneathiellales | Sphingomonadales | Thalassobaculales | Tistrellales | uncultured | Bdellovibrionales | Bradymonadales | Deltaproteobacteria_unclassified | Desulfarculales | Desulfovibrionales | MBNT15 | Myxococcales | NB1-j | Oligoflexales | PB19 | RCP2-54 | SAR324_clade(Marine_group_B) | Acidiferrobacterales | Acidithiobacillales | Alteromonadales | Betaproteobacteriales | CCD24 | CCM19a | Cellvibrionales | Competibacterales | Coxiellales | Diplorickettsiales | EV818SWSAP88 | Ectothiorhodospirales | Ga0077536 | Gammaproteobacteria_Incertae_Sedis | Gammaproteobacteria_unclassified | JG36-TzT-191 | KF-JG30-C25 | KI89A_clade | Legionellales | Nitrococcales | Nitrosococcales | Oceanospirillales | PLTA13 | Pseudomonadales | R7C24 | Salinisphaerales | Steroidobacterales | Tenderiales | Thiohalorhabdales | Xanthomonadales | Proteobacteria_unclassified | Rokubacteriales | Chthoniobacterales | Opitutales | Pedosphaerales | S-BQ2-57_soil_group | Verrucomicrobiae_unclassified | Verrucomicrobiales | WPS-2_or | WS2_or | Zixibacteria_or | unknown_unclassified |
|---|---|---|---|---|---|---|---|---|---|---|---|---|---|---|---|---|---|---|---|---|---|---|---|---|---|---|---|---|---|---|---|---|---|---|---|---|---|---|---|---|---|---|---|---|---|---|---|---|---|---|---|---|---|---|---|---|---|---|---|---|---|---|---|---|---|---|---|---|---|---|---|---|---|---|---|---|---|---|---|---|---|---|---|---|---|---|---|---|---|---|---|---|---|---|---|---|---|---|---|---|---|---|---|---|---|---|---|---|---|---|---|---|---|---|---|---|---|---|---|---|---|---|---|---|---|---|---|---|---|---|---|---|---|---|---|---|---|---|---|---|---|---|---|---|---|---|---|---|---|---|---|---|---|---|---|---|---|---|---|---|---|---|---|---|---|---|---|---|---|---|---|---|---|---|---|---|---|---|---|---|---|---|---|---|---|---|---|---|---|---|---|---|---|---|---|---|---|---|---|---|---|---|---|---|---|---|---|---|---|---|---|---|---|---|---|---|---|---|---|---|---|---|---|---|---|---|---|---|
| Sample_2.1 | 0.0196183342250758 | 0.740146245764223 | 0.0659889423934368 | 0.00356696985910469 | 4.522917781344748 | 0.0 | 0.00891742464776173 | 0.0142678794364188 | 0.00535045478865704 | 0.148029249152845 | 0.629570180131978 | 0.0178348492955235 | 0.00713393971820938 | 0.14446227929374 | 11.88336008560728 | 1.403602639557695 | 0.385232744783307 | 0.262172284644195 | 1.674692348849652 | 0.046370608168361 | 1.03442125914036 | 0.872124130551097 | 0.139111824505083 | 0.0 | 0.0927412163367219 | 0.0356696985910469 | 0.0231853040841805 | 0.103442125914036 | 27.85625111467808 | 0.0231853040841805 | 0.0 | 0.112359550561798 | 0.0160513643659711 | 0.137328339575531 | 0.0642054574638844 | 0.0 | 0.422685928303906 | 0.315676832530765 | 0.506509719992866 | 0.00178348492955235 | 0.0 | 0.00356696985910469 | 0.00535045478865704 | 0.283574103798823 | 0.0107009095773141 | 0.0 | 0.0178348492955235 | 0.00356696985910469 | 0.461922596754058 | 0.0 | 0.00891742464776173 | 0.0338862136614946 | 0.238986980560014 | 0.00535045478865704 | 0.0 | 0.0178348492955235 | 0.274656679151061 | 0.00535045478865704 | 0.0713393971820938 | 0.00356696985910469 | 0.0481540930979133 | 0.0624219725343321 | 0.176565008025682 | 0.0 | 0.322810772248975 | 0.0214018191546281 | 0.0 | 0.0178348492955235 | 0.0 | 0.977349741394685 | 0.00178348492955235 | 0.0410201533797039 | 0.0231853040841805 | 0.051721062957018 | 0.098091671125379 | 0.169431068307473 | 0.0 | 0.128410914927769 | 0.00535045478865704 | 0.00891742464776173 | 0.0 | 0.0 | 0.00178348492955235 | 0.0 | 0.00535045478865704 | 0.0 | 7.963260210451222 | 0.0 | 0.0 | 0.0 | 0.0 | 0.00535045478865704 | 0.0784733369003032 | 0.00178348492955235 | 0.0535045478865704 | 0.00713393971820938 | 0.00356696985910469 | 0.00891742464776173 | 0.00535045478865704 | 0.00891742464776173 | 0.0 | 0.0 | 0.0 | 0.0196183342250758 | 0.0 | 0.00535045478865704 | 0.0 | 0.0 | 0.0124843945068664 | 0.00891742464776173 | 0.051721062957018 | 1.970750847155341 | 0.0160513643659711 | 0.0428036383092563 | 0.0 | 0.422685928303906 | 0.0 | 1.153914749420367 | 0.171214553237025 | 1.342964151952916 | 0.00535045478865704 | 0.0 | 0.00356696985910469 | 0.0 | 0.00713393971820938 | 0.00356696985910469 | 0.00178348492955235 | 0.0178348492955235 | 0.0 | 0.00178348492955235 | 0.00356696985910469 | 0.0107009095773141 | 0.0392366684501516 | 0.287141073657928 | 0.00713393971820938 | 0.224719101123596 | 0.0 | 0.642054574638844 | 0.0 | 0.324594257178527 | 0.315676832530765 | 0.0267522739432852 | 0.0303192438023899 | 0.501159265204209 | 0.0606384876047797 | 0.756197610130195 | 0.447654717317639 | 0.046370608168361 | 0.057071517745675 | 0.0 | 0.201533797039415 | 0.0 | 0.998751560549313 | 0.0231853040841805 | 0.0 | 0.667023363652577 | 3.468878187979311 | 0.0445871232388086 | 0.0 | 0.0 | 0.0 | 0.0267522739432852 | 0.133761369716426 | 0.187265917602996 | 3.964686998394863 | 0.135544854645978 | 0.0909577314071696 | 0.0820403067594079 | 0.00356696985910469 | 0.0107009095773141 | 1.350098091671126 | 0.240770465489567 | 0.00535045478865704 | 0.579632602104512 | 0.0214018191546281 | 0.0 | 0.172998038166578 | 0.0820403067594079 | 0.0 | 0.0535045478865704 | 0.322810772248975 | 0.108792580702693 | 0.0 | 0.0 | 0.0249687890137328 | 0.0445871232388086 | 0.0998751560549313 | 0.0285357588728375 | 0.0 | 2.536115569823435 | 0.00356696985910469 | 0.219368646334938 | 0.0 | 0.0 | 0.0214018191546281 | 0.0196183342250758 | 0.0 | 0.0267522739432852 | 0.0392366684501516 | 0.00713393971820938 | 0.260388799714643 | 0.00178348492955235 | 0.00356696985910469 | 0.00713393971820938 | 0.0552880328161227 | 0.0356696985910469 | 0.171214553237025 | 0.0 | 2.573568753344034 | 0.0285357588728375 | 0.00178348492955235 | 0.576065632245408 | 1.416087034064562 | 0.0124843945068664 | 0.00178348492955235 | 3.65792759051186 | 0.00713393971820938 | 0.0428036383092563 | 0.0909577314071696 | 0.0838237916889602 | 0.0677724273229891 | 0.00535045478865704 | 0.00535045478865704 | 0.247904405207776 | 0.0 | 0.0 | 0.0731228821116462 | 0.00713393971820938 |
| Sample_3.1 | 0.0094509025611946 | 1.620829789244873 | 0.0 | 0.0 | 5.146016444570455 | 0.0 | 0.0047254512805973 | 0.0 | 0.0 | 0.0 | 0.363859748605992 | 0.0141763538417919 | 0.0141763538417919 | 0.0 | 0.0 | 0.155939892259711 | 0.0850581230507513 | 0.0 | 1.805122389188167 | 0.557603251110481 | 0.378036102447784 | 0.132312635856724 | 0.0661563179283622 | 0.0 | 0.779699461298554 | 0.8742084869105 | 0.0 | 3.8465173424062 | 10.35346375578868 | 0.0 | 0.0 | 0.0614308666477649 | 0.0 | 0.430016066534354 | 1.686986107173235 | 0.0 | 12.27199697571118 | 0.0283527076835838 | 0.694641338247803 | 0.0094509025611946 | 0.0 | 0.0 | 0.0141763538417919 | 0.189018051223892 | 0.0047254512805973 | 0.0 | 0.0094509025611946 | 0.496172384462716 | 2.060296758340422 | 0.619034117758246 | 0.0 | 0.0 | 0.0 | 0.0330781589641811 | 0.0 | 6.87080616198847 | 0.420565163973159 | 0.0 | 0.0 | 0.0 | 0.0 | 0.146488989698516 | 0.916737548435876 | 0.0 | 0.118136282014932 | 0.0047254512805973 | 0.0141763538417919 | 0.0 | 0.255174369152254 | 6.336830167280976 | 0.0 | 0.0 | 0.0 | 0.0094509025611946 | 0.103959928173141 | 0.0 | 0.0 | 0.0330781589641811 | 0.0 | 0.0047254512805973 | 0.0 | 0.108685379453738 | 0.760797656176165 | 0.0 | 3.444853983555429 | 0.0 | 8.19865797183631 | 0.0 | 0.0 | 0.0708817692089595 | 0.0 | 0.0 | 0.0378036102447784 | 0.0 | 0.0094509025611946 | 0.0 | 0.0 | 0.0189018051223892 | 0.0 | 0.0 | 0.0 | 0.0 | 0.0 | 0.0189018051223892 | 0.0 | 0.0094509025611946 | 0.0 | 0.0 | 0.0 | 0.0 | 0.0661563179283622 | 0.600132312635857 | 0.207919856346281 | 1.403459030337397 | 0.0 | 0.0661563179283622 | 0.0 | 0.0 | 0.0 | 0.0330781589641811 | 0.0 | 0.0 | 0.0 | 0.0 | 0.0 | 0.0 | 0.0 | 0.0 | 0.0 | 0.0 | 0.0 | 0.0 | 0.0 | 0.283527076835838 | 0.0 | 0.0047254512805973 | 0.0 | 0.0 | 0.132312635856724 | 0.151214440979114 | 0.141763538417919 | 0.0 | 0.0 | 0.500897835743314 | 0.0519799640865703 | 0.0519799640865703 | 0.240998015310462 | 0.0 | 0.0047254512805973 | 0.0 | 0.0330781589641811 | 0.0378036102447784 | 0.189018051223892 | 0.127587184576127 | 0.0 | 0.543426897268689 | 0.0 | 0.0 | 0.0 | 0.0 | 0.0 | 0.0 | 1.039599281731405 | 0.0 | 2.036669501937435 | 2.008316794253851 | 0.0283527076835838 | 1.606653435403081 | 0.0519799640865703 | 0.0 | 4.560060485776392 | 0.0897835743313486 | 0.127587184576127 | 0.0 | 0.080332671770154 | 0.0 | 0.0 | 0.0 | 0.0 | 0.0 | 0.222096210188073 | 0.0 | 0.538701445988092 | 0.0 | 0.0 | 0.0 | 0.0189018051223892 | 0.0 | 0.0 | 1.209715527832908 | 0.0 | 0.0 | 0.0 | 0.080332671770154 | 0.0 | 0.0 | 0.0 | 0.0708817692089595 | 0.0 | 0.0 | 0.160665343540308 | 0.0 | 0.0 | 0.0236272564029865 | 0.0141763538417919 | 3.89849730649277 | 0.0 | 2.083924014743408 | 0.0 | 0.0 | 0.0 | 0.0425290615253757 | 0.0756072204895568 | 0.0 | 0.0189018051223892 | 1.842925999432946 | 0.0 | 0.0 | 0.439466969095549 | 0.0141763538417919 | 0.0141763538417919 | 0.0 | 0.0 | 0.0 | 0.0 | 0.0 | 0.0 | 0.0 |
| Sample_3.2 | 0.0130639261452709 | 1.770161992684202 | 0.0 | 0.00435464204842362 | 12.61539801428323 | 0.00217732102421181 | 0.124107298380073 | 0.0 | 0.0 | 0.0 | 0.981971781919526 | 0.0217732102421181 | 0.0130639261452709 | 0.0 | 0.0239505312663299 | 0.13281658247692 | 0.0 | 0.00217732102421181 | 3.050426754920745 | 0.631423097021425 | 0.0718515937989897 | 0.0609649886779307 | 0.381031179237067 | 0.0 | 0.934070719386866 | 0.0217732102421181 | 0.0 | 3.113569064622888 | 2.763020379724787 | 0.0304824943389653 | 0.0 | 0.0174185681936945 | 0.0 | 0.291761017244383 | 0.010886605121059 | 0.0 | 3.866922139000173 | 0.0326598153631772 | 1.728792893224177 | 0.0174185681936945 | 0.0 | 0.0 | 0.0152412471694827 | 0.124107298380073 | 0.00217732102421181 | 0.0 | 0.0 | 1.317279219648145 | 0.966730534750044 | 0.00653196307263543 | 0.0958021250653196 | 0.0 | 0.0 | 0.0522557045810834 | 0.0 | 0.0 | 0.335307437728619 | 0.0 | 0.00870928409684724 | 0.0 | 0.0 | 0.990681066016373 | 0.52908900888347 | 0.0 | 0.0479010625326598 | 0.0870928409684724 | 0.0 | 0.0239505312663299 | 0.616181849851942 | 5.759014109040237 | 0.0 | 0.0 | 0.0 | 0.0239505312663299 | 0.261278522905417 | 0.00435464204842362 | 0.0 | 0.00870928409684724 | 0.0 | 0.108866051210591 | 0.0 | 0.010886605121059 | 0.00217732102421181 | 0.0195958892179063 | 1.336875108866051 | 0.00653196307263543 | 0.217732102421181 | 0.0 | 0.00217732102421181 | 25.0370144574116 | 0.00435464204842362 | 0.0 | 0.304824943389654 | 0.0 | 0.0 | 0.0 | 0.0 | 0.0892701619926842 | 0.0 | 0.0 | 0.0 | 0.0239505312663299 | 0.0 | 0.0 | 0.0 | 0.0 | 0.00217732102421181 | 0.0 | 0.0 | 0.0 | 0.0 | 0.581344713464553 | 0.0 | 0.158944434767462 | 0.0 | 0.080560877895837 | 0.0 | 0.0 | 0.0 | 0.137171224525344 | 0.0 | 0.0 | 0.0 | 0.0 | 0.0 | 0.0 | 0.0 | 0.00217732102421181 | 0.0 | 0.0 | 0.0 | 0.0 | 0.00435464204842362 | 0.363612611043372 | 0.0 | 0.00870928409684724 | 0.0 | 0.0 | 0.0 | 0.0413690994600244 | 0.487719909423445 | 0.0 | 0.0 | 0.618359170876154 | 0.0435464204842362 | 0.100156767113743 | 0.232973349590664 | 0.00435464204842362 | 0.0 | 0.0 | 0.178540323985368 | 0.0 | 0.696742727747779 | 0.0653196307263543 | 0.00217732102421181 | 0.339662079777042 | 0.0 | 0.0 | 0.0 | 0.0 | 0.0 | 0.0 | 0.139348545549556 | 0.0 | 1.317279219648145 | 5.876589444347675 | 0.0 | 0.0 | 0.0304824943389653 | 0.0 | 7.108953144051559 | 0.00435464204842362 | 0.0870928409684724 | 0.00870928409684724 | 0.0653196307263543 | 0.0 | 0.126284619404285 | 0.0 | 0.0 | 0.0 | 1.474046333391395 | 0.0 | 0.779480926667828 | 0.0 | 0.0 | 0.0 | 0.0239505312663299 | 0.0 | 0.0 | 0.879637693781571 | 0.0 | 0.0 | 0.0 | 0.0 | 0.0 | 0.00435464204842362 | 0.00870928409684724 | 0.0 | 0.0 | 0.0 | 4.964291935202926 | 0.0 | 0.0 | 0.0 | 0.00870928409684724 | 0.0 | 0.00217732102421181 | 0.00435464204842362 | 0.0 | 0.0 | 0.0 | 0.0 | 0.00217732102421181 | 0.0 | 0.0 | 0.592231318585612 | 0.034837136387389 | 0.0 | 0.907942867096325 | 0.0566103466295071 | 0.161121755791674 | 0.0 | 0.0 | 0.0 | 0.010886605121059 | 0.0 | 0.0 | 0.0 |
| Sample_3.3 | 0.0 | 1.556381162767346 | 0.0 | 0.0 | 4.844863942162868 | 0.0 | 0.0100411687920474 | 0.0 | 0.0 | 0.0 | 0.481976102018275 | 0.0050205843960237 | 0.0100411687920474 | 0.0 | 0.0 | 0.225926297821066 | 0.115473441108545 | 0.0 | 1.747163369816247 | 0.552264283562607 | 0.346420323325635 | 0.266090972989256 | 0.140576363088664 | 0.0 | 0.783211165779696 | 0.712922984235365 | 0.0251029219801185 | 3.534491414800683 | 9.001907822070487 | 0.0 | 0.0 | 0.0652675971483081 | 0.0 | 0.572346621146701 | 1.646751681895773 | 0.0 | 13.56059845366001 | 0.0251029219801185 | 0.587408374334773 | 0.0050205843960237 | 0.0 | 0.0 | 0.0050205843960237 | 0.155638116276735 | 0.0050205843960237 | 0.0 | 0.0150617531880711 | 0.486996686414299 | 2.319509990962948 | 0.707902399839341 | 0.0 | 0.0 | 0.0 | 0.0301235063761422 | 0.0 | 6.838035947384276 | 0.336379154533588 | 0.0 | 0.0 | 0.0 | 0.0 | 0.160658700672758 | 0.818355256551863 | 0.0 | 0.0251029219801185 | 0.0251029219801185 | 0.0 | 0.0050205843960237 | 0.246008635405161 | 6.742644843859825 | 0.0 | 0.0 | 0.0 | 0.050205843960237 | 0.0803293503363792 | 0.0100411687920474 | 0.0050205843960237 | 0.0150617531880711 | 0.0 | 0.0150617531880711 | 0.0 | 0.105432272316498 | 0.778190581383673 | 0.0 | 4.418114268500852 | 0.0 | 7.887338086153228 | 0.0 | 0.0 | 0.0301235063761422 | 0.0 | 0.0 | 0.0803293503363792 | 0.0 | 0.0301235063761422 | 0.0 | 0.0 | 0.0251029219801185 | 0.0 | 0.0 | 0.0 | 0.0 | 0.0 | 0.0150617531880711 | 0.0 | 0.0150617531880711 | 0.0 | 0.0 | 0.0 | 0.0 | 0.0301235063761422 | 0.587408374334773 | 0.125514609900592 | 1.611607591123607 | 0.0150617531880711 | 0.0953911035244502 | 0.0 | 0.0 | 0.0 | 0.0100411687920474 | 0.0 | 0.0 | 0.0 | 0.0 | 0.0 | 0.0 | 0.0 | 0.0 | 0.0 | 0.0 | 0.0 | 0.0 | 0.0 | 0.256049804197209 | 0.0 | 0.0 | 0.0 | 0.0 | 0.125514609900592 | 0.256049804197209 | 0.13555577869264 | 0.0 | 0.0 | 0.592428958730796 | 0.0401646751681896 | 0.0953911035244502 | 0.210864544632995 | 0.0 | 0.0 | 0.0 | 0.0200823375840948 | 0.0451852595642133 | 0.130535194296616 | 0.145596947484687 | 0.0 | 0.476955517622251 | 0.0 | 0.0 | 0.0 | 0.0 | 0.0100411687920474 | 0.0 | 1.024199216788834 | 0.0 | 1.98313083642936 | 1.932924992469123 | 0.0200823375840948 | 1.455969474846872 | 0.050205843960237 | 0.0 | 4.222311477055928 | 0.115473441108545 | 0.125514609900592 | 0.0150617531880711 | 0.0451852595642133 | 0.0 | 0.0050205843960237 | 0.0 | 0.0 | 0.0 | 0.155638116276735 | 0.0 | 0.451852595642133 | 0.0 | 0.0 | 0.0 | 0.050205843960237 | 0.0 | 0.0 | 1.15473441108545 | 0.0 | 0.0 | 0.0 | 0.0803293503363792 | 0.0 | 0.0 | 0.0 | 0.0150617531880711 | 0.0 | 0.0050205843960237 | 0.281152726177327 | 0.0 | 0.0 | 0.0552264283562607 | 0.0100411687920474 | 3.69515011547344 | 0.0150617531880711 | 2.229139471834522 | 0.0 | 0.0 | 0.0 | 0.0602470127522844 | 0.125514609900592 | 0.0 | 0.0 | 2.033336680389598 | 0.0 | 0.0 | 0.246008635405161 | 0.0 | 0.0200823375840948 | 0.0 | 0.0 | 0.0 | 0.0 | 0.0 | 0.0 | 0.0 |
| Sample_3.6 | 0.0 | 0.0 | 0.0 | 0.0 | 0.0 | 0.0 | 0.0 | 0.0 | 0.0 | 0.0 | 0.0 | 0.0 | 0.0 | 0.0 | 0.0 | 0.0 | 0.0 | 0.0 | 0.00542829225925524 | 0.0 | 0.0 | 0.0271414612962762 | 0.0 | 0.0 | 0.0 | 0.0597112148518076 | 0.0 | 0.0135707306481381 | 0.00542829225925524 | 0.0 | 0.0 | 0.127564868092498 | 0.0 | 0.0 | 0.119422429703615 | 0.0 | 65.32678319400715 | 0.00271414612962762 | 0.0 | 0.0 | 0.0 | 0.0 | 0.0 | 0.0814243838888286 | 0.00271414612962762 | 0.0244273151666486 | 0.0 | 0.0 | 0.0 | 4.280208446422756 | 0.0 | 0.0 | 0.0 | 0.0 | 0.0 | 28.53110411464554 | 0.0 | 0.0 | 0.0 | 0.0 | 0.0 | 0.0 | 0.0 | 0.0 | 0.0 | 0.0 | 0.0 | 0.0 | 0.0 | 0.0 | 0.0 | 0.0 | 0.0 | 0.0 | 0.0 | 0.0 | 0.0 | 0.0 | 0.0 | 0.0 | 0.0 | 0.0 | 0.0 | 0.0 | 0.0 | 0.0 | 0.0108565845185105 | 0.0 | 0.0 | 0.0 | 0.0 | 0.0 | 0.0 | 0.0 | 0.0 | 0.0 | 0.0 | 0.0 | 0.0 | 0.0 | 0.00542829225925524 | 0.0 | 0.0 | 0.0434263380740419 | 0.0135707306481381 | 0.021713169037021 | 0.0 | 0.0 | 0.0 | 0.0 | 0.0 | 0.0 | 0.0 | 0.0 | 0.0 | 0.0 | 0.0 | 0.0 | 0.0 | 0.0 | 0.0 | 0.0 | 0.0 | 0.0 | 0.0 | 0.0 | 0.0 | 0.0 | 0.0 | 0.0 | 0.0 | 0.0 | 0.0 | 0.0 | 0.0 | 0.0 | 0.0 | 0.0 | 0.0 | 0.0 | 0.0 | 0.0 | 0.0 | 0.0 | 0.0 | 0.0 | 0.0 | 0.0 | 0.0 | 0.0 | 0.0 | 0.0 | 0.00542829225925524 | 0.0 | 0.0 | 0.0 | 0.0 | 0.0 | 0.0 | 0.0 | 0.0 | 0.0 | 0.0 | 0.0 | 0.0 | 0.165562913907285 | 0.0 | 0.0 | 0.0 | 0.0 | 0.0 | 0.0 | 0.0 | 0.0 | 0.0 | 0.0 | 0.0 | 0.0 | 0.0 | 0.0 | 0.0 | 0.0 | 0.0 | 0.0 | 0.0 | 0.0 | 0.0 | 0.0 | 0.0 | 0.0 | 0.0 | 0.0 | 0.0 | 0.0 | 0.0 | 0.0678536532406905 | 0.0 | 0.0 | 0.0 | 0.0 | 0.0 | 0.0 | 0.0 | 0.0 | 0.0 | 0.108565845185105 | 0.0 | 0.488546303332972 | 0.0 | 0.0 | 0.0 | 0.461404842036695 | 0.0 | 0.0 | 0.0 | 0.0 | 0.0 | 0.0 | 0.0 | 0.0 | 0.0 | 0.0 | 0.0 | 0.0 | 0.0 | 0.0 | 0.0 | 0.0 |
| Sample_4.2 | 0.0 | 3.554237098399194 | 0.0 | 0.0 | 6.190529497369305 | 0.0 | 0.0 | 0.0 | 0.0 | 0.0 | 0.470166797268555 | 0.0167916713310198 | 0.0167916713310198 | 0.391805664390462 | 0.0 | 0.0223888951080264 | 0.29665286018135 | 0.0951528042091123 | 2.082167245046457 | 0.0 | 0.537333482592634 | 0.0783611328780925 | 0.29665286018135 | 0.0 | 0.027986118885033 | 0.145527818202172 | 0.0 | 0.0223888951080264 | 0.403000111944476 | 0.0 | 0.0 | 0.0111944475540132 | 0.0 | 0.0391805664390462 | 0.0223888951080264 | 0.0 | 0.291055636404343 | 0.414194559498489 | 0.162319489533192 | 0.0111944475540132 | 0.0111944475540132 | 0.0 | 0.00559722377700661 | 0.828389118996977 | 0.00559722377700661 | 0.00559722377700661 | 0.0839583566550991 | 3.89007052501959 | 1.074666965185268 | 0.839583566550991 | 0.145527818202172 | 0.0 | 0.0 | 0.123138923094145 | 0.699652972125826 | 0.0 | 0.190305608418225 | 0.0 | 0.380611216836449 | 0.0111944475540132 | 0.0 | 0.156722265756185 | 0.212694503526251 | 1.919847755513265 | 0.917944699429083 | 0.00559722377700661 | 0.0 | 0.0111944475540132 | 0.0 | 0.582111272808687 | 0.0 | 0.027986118885033 | 0.0 | 0.106347251763125 | 0.212694503526251 | 0.123138923094145 | 0.0 | 0.173513937087205 | 0.0 | 0.0 | 1.136236426732341 | 0.0503750139930594 | 0.0 | 3.87327885368857 | 3.078473077353633 | 0.0111944475540132 | 0.128736146871152 | 0.0111944475540132 | 0.0 | 0.0 | 0.0 | 0.0 | 0.027986118885033 | 0.0 | 0.0447777902160528 | 0.0 | 0.0 | 0.0 | 0.0 | 0.0 | 0.0 | 0.0 | 0.0111944475540132 | 0.55972237770066 | 0.0 | 0.0167916713310198 | 0.0 | 0.0 | 0.0 | 0.0 | 0.0223888951080264 | 0.867569685436024 | 0.190305608418225 | 0.31344453151237 | 0.0 | 0.0503750139930594 | 0.0167916713310198 | 0.0 | 0.0 | 0.64368073435576 | 0.0223888951080264 | 0.0111944475540132 | 0.0 | 0.0727639091010859 | 0.0 | 0.00559722377700661 | 0.0 | 0.0 | 0.0111944475540132 | 0.0 | 0.0 | 0.0 | 0.0 | 1.600806000223889 | 0.0 | 0.184708384641218 | 0.0335833426620396 | 0.0111944475540132 | 0.00559722377700661 | 0.481361244822568 | 0.800403000111944 | 0.0 | 0.0 | 1.052278070077242 | 0.0391805664390462 | 0.363819545505429 | 0.0615694615470727 | 0.0167916713310198 | 0.00559722377700661 | 0.229486174857271 | 1.998208888391358 | 0.0 | 1.113847531624315 | 0.0 | 0.0 | 1.835889398858166 | 0.0 | 0.027986118885033 | 0.100750027986119 | 0.0111944475540132 | 0.0 | 0.0503750139930594 | 0.167916713310198 | 0.750027986118885 | 2.009403335945371 | 1.50005597223777 | 0.111944475540132 | 0.0559722377700661 | 0.307847307735363 | 0.128736146871152 | 11.42953095264749 | 0.00559722377700661 | 0.996305832307176 | 0.34702787417441 | 0.537333482592634 | 0.0 | 0.0223888951080264 | 0.733236314787865 | 0.0 | 0.167916713310198 | 0.850778014105004 | 0.0839583566550991 | 0.218291727303258 | 0.0 | 0.0 | 0.0503750139930594 | 0.0 | 0.0 | 0.0 | 17.53050486958469 | 0.0 | 1.975819993283332 | 0.0 | 0.0 | 0.027986118885033 | 0.179111160864211 | 0.0 | 0.0 | 0.0727639091010859 | 0.0 | 4.701667972685548 | 0.0 | 0.0 | 0.0727639091010859 | 0.00559722377700661 | 0.0 | 0.0 | 0.0 | 1.1866114407254 | 0.0335833426620396 | 0.0 | 0.0 | 0.285458412627337 | 0.0111944475540132 | 0.0 | 2.843389678719355 | 0.0223888951080264 | 0.0 | 0.246277846188291 | 0.425389007052502 | 0.0391805664390462 | 0.0 | 0.0 | 0.156722265756185 | 0.0 | 0.162319489533192 | 0.0 | 0.0 |
| Sample_4.5 | 0.0147790531553278 | 0.187201339967486 | 0.0 | 0.0 | 0.093600669983743 | 0.0 | 0.0 | 0.507414158332923 | 0.0 | 0.0 | 0.403960786245628 | 0.0098527021035519 | 0.0295581063106557 | 0.0443371594659835 | 0.241391201537022 | 0.561604019902458 | 8.611261638504358 | 0.290654712054781 | 1.58135868762008 | 0.758658061973496 | 0.950785752992758 | 0.0246317552588798 | 0.0738952657766392 | 0.0098527021035519 | 0.118232425242623 | 0.54682496674713 | 0.832553327750135 | 1.221735060840436 | 46.72643972609488 | 0.128085127346175 | 0.0443371594659835 | 0.137937829449727 | 0.147790531553278 | 1.078870880338933 | 0.433518892556284 | 0.00492635105177595 | 11.31582836592936 | 0.0689689147248633 | 0.448297945711611 | 0.0098527021035519 | 0.0 | 0.0 | 0.0147790531553278 | 0.295581063106557 | 0.0 | 0.0 | 0.0 | 0.0 | 0.605941179368442 | 0.468003349918715 | 0.0 | 0.0 | 0.0 | 0.0 | 0.0 | 0.315286467313661 | 0.0 | 0.0 | 0.0 | 0.00492635105177595 | 0.0197054042071038 | 0.0344844573624316 | 0.0738952657766392 | 0.0 | 0.118232425242623 | 0.0246317552588798 | 0.0 | 0.0541898615695354 | 0.0 | 4.473126755012563 | 0.00492635105177595 | 0.0147790531553278 | 0.201980393122814 | 0.408887137297404 | 0.113306074190847 | 0.0197054042071038 | 0.0 | 0.0394108084142076 | 0.0 | 0.0147790531553278 | 0.0 | 0.0 | 0.0 | 0.0 | 0.187201339967486 | 0.0 | 0.0 | 0.0 | 0.0 | 0.0 | 0.0 | 0.0 | 0.246317552588797 | 0.0 | 0.0344844573624316 | 0.0 | 0.0 | 0.0 | 0.0 | 0.0 | 0.0 | 0.0 | 0.0 | 0.0197054042071038 | 0.0 | 0.0344844573624316 | 0.00492635105177595 | 0.0197054042071038 | 0.0 | 0.0 | 0.226612148381694 | 0.448297945711611 | 0.300507414158333 | 0.251243903640573 | 0.0 | 0.857185083009015 | 0.00492635105177595 | 0.0 | 0.0 | 0.162569584708606 | 0.0 | 0.0 | 0.0 | 0.0 | 0.0 | 0.0 | 0.0 | 0.0 | 0.0 | 0.0 | 0.0 | 0.0 | 0.0 | 0.0098527021035519 | 0.0 | 0.0738952657766392 | 0.0 | 0.0 | 0.0098527021035519 | 0.0394108084142076 | 0.0443371594659835 | 0.0 | 0.0 | 0.0197054042071038 | 0.093600669983743 | 0.305433765210109 | 0.492635105177595 | 0.0 | 0.0 | 0.0 | 0.0197054042071038 | 0.0 | 0.408887137297404 | 0.0 | 0.0 | 0.18227498891571 | 0.0 | 0.0394108084142076 | 0.0 | 0.0 | 0.0 | 0.0 | 0.280802009951229 | 0.0147790531553278 | 2.596187004285925 | 0.108379723139071 | 0.0098527021035519 | 0.118232425242623 | 0.0 | 0.0 | 0.59608847726489 | 0.0 | 0.0738952657766392 | 1.083797231390709 | 0.0 | 0.0 | 0.00492635105177595 | 0.0 | 0.0 | 0.0197054042071038 | 0.0492635105177595 | 0.0 | 0.0 | 0.0 | 0.0 | 0.0 | 0.0098527021035519 | 0.0886743189319671 | 0.0 | 0.463076998866939 | 0.0 | 0.0 | 0.0 | 0.0 | 0.0197054042071038 | 0.0197054042071038 | 0.0 | 0.0246317552588798 | 0.0147790531553278 | 0.0 | 0.517266860436475 | 0.0 | 0.0 | 0.0344844573624316 | 0.0147790531553278 | 0.15764323365683 | 0.0 | 0.0492635105177595 | 0.502487807281147 | 0.0147790531553278 | 0.0 | 0.0147790531553278 | 2.83265185477117 | 0.0 | 0.0443371594659835 | 0.556677668850682 | 0.0147790531553278 | 0.0246317552588798 | 0.0 | 0.0197054042071038 | 0.0591162126213114 | 0.0 | 0.0 | 0.0295581063106557 | 0.0 | 0.0 | 0.0 | 0.0 |
| Sample_4.6 | 0.0 | 0.0 | 0.0 | 0.0 | 0.0117633219621221 | 0.0 | 0.0 | 0.0 | 0.0 | 0.0 | 0.0 | 0.0 | 0.0 | 0.0 | 0.0 | 0.0235266439242442 | 0.0117633219621221 | 0.0 | 2.258557816727444 | 0.384268517429322 | 0.431321805277811 | 0.0196055366035368 | 1.78802493824256 | 0.0666588244520253 | 0.529349488295495 | 14.4767282280516 | 0.80382700074501 | 0.59600831274752 | 5.466023605066071 | 0.0 | 0.0 | 0.0627377171313179 | 0.0 | 1.235148806022821 | 1.278280986550602 | 0.0235266439242442 | 34.4312433831314 | 0.0 | 0.0 | 0.0 | 0.0 | 0.0 | 0.0313688585656589 | 0.0313688585656589 | 0.0 | 0.00784221464141474 | 0.0 | 0.0 | 1.254754342626358 | 0.0156844292828295 | 0.0 | 0.0 | 0.0 | 0.0 | 0.0 | 0.0352899658863663 | 0.0 | 0.0 | 0.0 | 0.0 | 0.0 | 0.0 | 0.0 | 0.0 | 0.0 | 0.0 | 0.0 | 0.0 | 0.0 | 1.670391718621339 | 0.0 | 0.0 | 0.0 | 0.0 | 0.0 | 0.0 | 0.0 | 0.0 | 0.0 | 0.0 | 0.0 | 0.0 | 0.948907971611183 | 0.0 | 0.00784221464141474 | 0.0 | 4.211269262439713 | 0.203897580676783 | 2.611457475591107 | 0.0 | 0.0 | 0.0 | 0.0117633219621221 | 0.0 | 0.0 | 0.0 | 0.0 | 0.0 | 0.0 | 0.0 | 0.0 | 0.0 | 0.0 | 0.0117633219621221 | 0.0156844292828295 | 0.00784221464141474 | 0.0 | 0.0 | 0.0 | 0.0 | 0.0 | 0.0117633219621221 | 0.0 | 0.0 | 0.0 | 0.0 | 0.0 | 0.0 | 0.0 | 0.0 | 0.0 | 0.0 | 0.0 | 0.0 | 0.0 | 0.0 | 0.0 | 0.0 | 0.0 | 0.0 | 0.0 | 0.0 | 0.0 | 0.0588166098106105 | 0.0 | 0.0 | 0.0 | 0.0 | 0.0 | 0.0 | 0.0235266439242442 | 0.0 | 0.0 | 0.0 | 0.0 | 0.0 | 0.0 | 0.0 | 0.0 | 0.0 | 0.0 | 0.0 | 0.0862643610555621 | 0.0 | 0.0 | 0.0 | 0.0 | 0.0 | 0.0 | 0.0 | 0.0 | 0.0352899658863663 | 0.101948790338392 | 0.0 | 8.25393091008901 | 0.576402776143983 | 0.0 | 0.650903815237423 | 0.0 | 0.0 | 10.56346312198565 | 0.0 | 0.0 | 0.0 | 0.0 | 0.0 | 0.0 | 0.0 | 0.0 | 0.0 | 0.0196055366035368 | 0.0 | 0.0 | 0.0 | 0.0 | 0.0 | 0.0 | 0.0 | 0.0235266439242442 | 0.00784221464141474 | 0.0 | 0.0 | 0.0 | 0.0 | 0.0 | 0.0 | 0.0 | 0.0 | 0.0 | 0.0 | 0.0313688585656589 | 0.0 | 0.0 | 0.0470532878484884 | 0.0 | 0.874406932517743 | 0.0 | 0.337215229580834 | 0.0 | 0.0 | 0.0 | 0.0 | 0.348978551542956 | 0.0 | 0.278398619770223 | 2.682037407363839 | 0.0 | 0.0 | 0.0 | 0.0117633219621221 | 0.0 | 0.0 | 0.0 | 0.0 | 0.0 | 0.0 | 0.0 | 0.0 |
| Sample_4.7 | 0.00336496399488525 | 2.856854431657581 | 0.0 | 0.0 | 9.334410121811695 | 0.0437445319335083 | 1.362810417928528 | 0.0 | 0.0 | 0.0 | 0.370146039437378 | 0.0201897839693115 | 0.0168248199744263 | 0.0 | 0.0807591358772462 | 0.275927047580591 | 0.0437445319335083 | 0.00672992798977051 | 2.880409179621778 | 0.104313883841443 | 0.131233595800525 | 0.00672992798977051 | 0.235547479641968 | 0.0 | 0.437445319335083 | 0.0302846759539673 | 0.0 | 7.419745608721986 | 3.159701191197254 | 0.0 | 0.0 | 0.0302846759539673 | 0.0 | 0.218722659667542 | 0.0168248199744263 | 0.0 | 24.81660946227876 | 0.00672992798977051 | 1.897839693115283 | 0.00336496399488525 | 0.0 | 0.0504744599232788 | 0.0 | 0.121138703815869 | 0.00336496399488525 | 0.0 | 0.00336496399488525 | 5.865132243084997 | 1.110438118312134 | 0.0 | 0.0100948919846558 | 0.0 | 0.0 | 0.00672992798977051 | 0.0 | 0.0 | 2.018978396931153 | 0.00672992798977051 | 0.0 | 0.0 | 0.0 | 1.376270273908069 | 0.114408775826099 | 0.0 | 0.0100948919846558 | 0.0201897839693115 | 0.00672992798977051 | 0.0100948919846558 | 0.306211723534558 | 4.60327074500303 | 0.0 | 0.0100948919846558 | 0.0 | 0.0773941718823609 | 0.164883235749378 | 0.0 | 0.0 | 0.0 | 0.0 | 0.013459855979541 | 0.0 | 0.0 | 0.0168248199744263 | 0.0 | 0.0437445319335083 | 0.0 | 0.00336496399488525 | 0.0 | 0.0 | 0.0 | 0.00336496399488525 | 0.0 | 0.0908540278619019 | 0.0 | 0.0 | 0.0 | 0.0 | 0.0 | 0.0 | 0.0 | 0.0 | 0.0605693519079346 | 0.0 | 0.0168248199744263 | 0.0 | 0.013459855979541 | 0.0 | 0.0 | 0.0 | 0.0 | 0.00672992798977051 | 0.864795746685511 | 0.0336496399488525 | 0.215357695672656 | 0.0 | 0.0235547479641968 | 0.0 | 0.0 | 0.0 | 0.0302846759539673 | 0.0 | 0.0 | 0.0 | 0.013459855979541 | 0.0 | 0.0 | 0.0 | 0.0 | 0.0 | 0.0 | 0.0 | 0.0 | 0.013459855979541 | 0.296116831549902 | 0.0 | 0.0 | 0.0 | 0.0 | 0.0370146039437378 | 0.0168248199744263 | 1.241671714112659 | 0.0 | 0.0 | 0.5249343832021 | 0.0706642438925904 | 0.164883235749378 | 0.0302846759539673 | 0.0 | 0.0 | 0.0 | 0.0100948919846558 | 0.0 | 0.373511003432263 | 0.00672992798977051 | 0.0 | 0.720102294905444 | 0.0 | 0.0 | 0.0 | 0.0 | 0.0168248199744263 | 0.0 | 0.188437983713574 | 0.0 | 1.090248334342823 | 4.128810821724206 | 0.0 | 0.0100948919846558 | 0.013459855979541 | 0.0 | 11.38030823070193 | 0.0201897839693115 | 0.242277407631738 | 0.0 | 0.0 | 0.0 | 0.0 | 0.0 | 0.0 | 0.0 | 0.171613163739148 | 0.00672992798977051 | 0.205262803688001 | 0.0201897839693115 | 0.0 | 0.0 | 0.0 | 0.0 | 0.0 | 2.570832492092335 | 0.0 | 0.0 | 0.0 | 0.0 | 0.0 | 0.0 | 0.0 | 0.0 | 0.0 | 0.00672992798977051 | 1.655562285483545 | 0.0 | 0.0 | 0.0 | 0.0 | 0.0100948919846558 | 0.0 | 0.0 | 0.0 | 0.0 | 0.0 | 0.0 | 0.00672992798977051 | 0.0 | 0.0 | 0.905175314624134 | 0.0 | 0.0 | 0.107678847836328 | 0.572043879130493 | 0.0 | 0.0 | 0.0 | 0.0 | 0.00672992798977051 | 0.0 | 0.0 | 0.0 |
| Sample_4.8 | 0.0 | 0.0 | 0.0 | 0.0 | 0.0 | 0.0 | 0.0 | 0.0 | 0.0 | 0.0 | 0.0 | 0.0 | 0.0 | 0.0 | 0.0 | 0.0509113124936361 | 0.078064012490242 | 0.00339408749957574 | 1.221871499847266 | 0.105216712486848 | 0.0610935749923633 | 0.00678817499915148 | 0.00678817499915148 | 0.0 | 0.0203645249974544 | 1.014832162373146 | 0.0 | 0.851915962393511 | 5.627397074296574 | 0.0 | 0.0 | 0.0441231374944846 | 0.00678817499915148 | 0.224009774971999 | 0.264738824966908 | 0.00678817499915148 | 26.53158198418355 | 0.0 | 0.410684587448665 | 0.00339408749957574 | 0.0 | 0.0 | 0.013576349998303 | 0.0610935749923633 | 0.0 | 0.0 | 0.0 | 0.0 | 10.31463191121067 | 1.320300037334962 | 0.0543053999932118 | 0.0 | 0.0 | 0.0 | 0.0 | 0.00678817499915148 | 17.3336048603333 | 0.0 | 0.0 | 0.0 | 0.0 | 0.0101822624987272 | 0.0 | 0.0 | 0.0 | 0.0 | 0.0 | 0.0169704374978787 | 0.0 | 4.772087024403489 | 0.0 | 0.0 | 0.00678817499915148 | 0.00678817499915148 | 0.00678817499915148 | 0.0 | 0.0 | 0.0 | 0.0 | 0.0 | 0.0 | 0.0 | 0.0 | 0.0 | 1.771713674778536 | 0.0 | 0.0 | 0.0 | 0.0 | 0.0 | 0.0 | 0.0 | 0.458201812442725 | 0.0 | 0.0101822624987272 | 0.0 | 0.0 | 0.0 | 0.0 | 0.0 | 0.0 | 0.0 | 0.0 | 0.0 | 0.0 | 0.0169704374978787 | 0.0 | 0.0 | 0.0 | 0.0 | 0.00678817499915148 | 0.0373349624953331 | 0.13576349998303 | 0.556630349930421 | 0.0 | 0.0 | 0.0 | 0.0 | 0.0 | 0.0 | 0.0 | 0.0 | 0.0 | 0.0 | 0.0 | 0.0 | 0.0 | 0.00678817499915148 | 0.0 | 0.0 | 0.0 | 0.0 | 0.00678817499915148 | 0.013576349998303 | 0.0 | 0.0 | 0.0 | 0.0 | 0.00678817499915148 | 0.091640362488545 | 0.200251162474969 | 0.0 | 0.0 | 0.00339408749957574 | 0.078064012490242 | 0.00678817499915148 | 0.190068899976241 | 0.0 | 0.00339408749957574 | 0.0 | 0.0 | 0.0 | 0.0475172249940604 | 0.0 | 0.0 | 0.702576112412178 | 0.0 | 0.0 | 0.0 | 0.0 | 0.0237586124970302 | 0.0 | 0.0814580999898177 | 0.0 | 4.154363099480705 | 0.481960424939755 | 0.0 | 0.013576349998303 | 0.0101822624987272 | 0.0 | 16.28822591046397 | 0.0 | 0.946950412381631 | 0.0 | 0.0 | 0.013576349998303 | 0.0 | 0.0 | 0.00339408749957574 | 0.0 | 0.0 | 0.0 | 0.00678817499915148 | 0.0 | 0.0 | 0.0 | 0.0 | 0.0 | 0.0 | 0.0169704374978787 | 0.0 | 0.0 | 0.00678817499915148 | 0.0 | 0.0 | 0.0 | 0.0 | 0.0 | 0.0 | 0.0101822624987272 | 0.648270712418966 | 0.0 | 0.0 | 0.0 | 0.0 | 0.105216712486848 | 0.0 | 1.079319824865085 | 0.0 | 0.0 | 0.0 | 0.0 | 0.0339408749957574 | 0.0 | 0.0 | 1.232053762345993 | 0.00339408749957574 | 0.0 | 0.0 | 0.0237586124970302 | 0.0 | 0.0 | 0.0 | 0.0 | 0.0 | 0.0 | 0.0 | 0.0 |Relative abundance (%)
Supplementary Figure S2(c). Relative abundance of bacterial 16S rRNA genes at order taxonomic rank.

## Slide 4
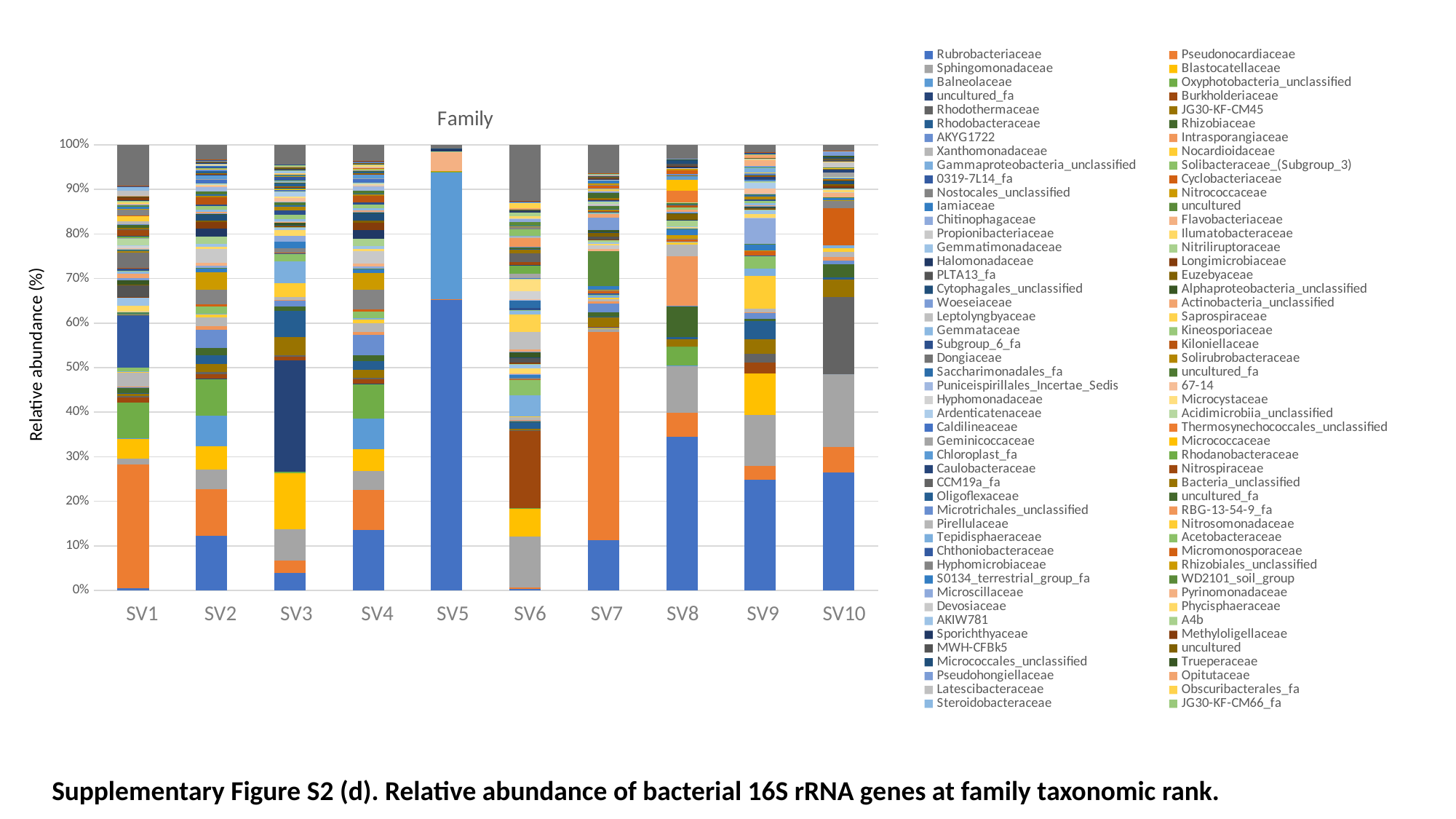

### Chart: Family
| Category | Rubrobacteriaceae | Pseudonocardiaceae | Sphingomonadaceae | Blastocatellaceae | Balneolaceae | Oxyphotobacteria_unclassified | uncultured_fa | Burkholderiaceae | Rhodothermaceae | JG30-KF-CM45 | Rhodobacteraceae | Rhizobiaceae | AKYG1722 | Intrasporangiaceae | Xanthomonadaceae | Nocardioidaceae | Gammaproteobacteria_unclassified | Solibacteraceae_(Subgroup_3) | 0319-7L14_fa | Cyclobacteriaceae | Nostocales_unclassified | Nitrococcaceae | Iamiaceae | uncultured | Chitinophagaceae | Flavobacteriaceae | Propionibacteriaceae | Ilumatobacteraceae | Gemmatimonadaceae | Nitriliruptoraceae | Halomonadaceae | Longimicrobiaceae | PLTA13_fa | Euzebyaceae | Cytophagales_unclassified | Alphaproteobacteria_unclassified | Woeseiaceae | Actinobacteria_unclassified | Leptolyngbyaceae | Saprospiraceae | Gemmataceae | Kineosporiaceae | Subgroup_6_fa | Kiloniellaceae | Dongiaceae | Solirubrobacteraceae | Saccharimonadales_fa | uncultured_fa | Puniceispirillales_Incertae_Sedis | 67-14 | Hyphomonadaceae | Microcystaceae | Ardenticatenaceae | Acidimicrobiia_unclassified | Caldilineaceae | Thermosynechococcales_unclassified | Geminicoccaceae | Micrococcaceae | Chloroplast_fa | Rhodanobacteraceae | Caulobacteraceae | Nitrospiraceae | CCM19a_fa | Bacteria_unclassified | Oligoflexaceae | uncultured_fa | Microtrichales_unclassified | RBG-13-54-9_fa | Pirellulaceae | Nitrosomonadaceae | Tepidisphaeraceae | Acetobacteraceae | Chthoniobacteraceae | Micromonosporaceae | Hyphomicrobiaceae | Rhizobiales_unclassified | S0134_terrestrial_group_fa | WD2101_soil_group | Microscillaceae | Pyrinomonadaceae | Devosiaceae | Phycisphaeraceae | AKIW781 | A4b | Sporichthyaceae | Methyloligellaceae | MWH-CFBk5 | uncultured | Micrococcales_unclassified | Trueperaceae | Pseudohongiellaceae | Opitutaceae | Latescibacteraceae | Obscuribacterales_fa | Steroidobacteraceae | JG30-KF-CM66_fa | Beijerinckiaceae | Gemmatimonadetes_unclassified | Remaining families |
|---|---|---|---|---|---|---|---|---|---|---|---|---|---|---|---|---|---|---|---|---|---|---|---|---|---|---|---|---|---|---|---|---|---|---|---|---|---|---|---|---|---|---|---|---|---|---|---|---|---|---|---|---|---|---|---|---|---|---|---|---|---|---|---|---|---|---|---|---|---|---|---|---|---|---|---|---|---|---|---|---|---|---|---|---|---|---|---|---|---|---|---|---|---|---|---|---|---|---|---|
| Sample_2.1 | 0.422685928303906 | 27.85625111467808 | 1.350098091671126 | 4.522917781344748 | 0.0178348492955235 | 7.963260210451222 | 0.0 | 1.1788835384341 | 0.274656679151061 | 0.574282147315855 | 0.135544854645978 | 1.079008382379169 | 0.37988228999465 | 0.0285357588728375 | 3.167469234884965 | 0.00535045478865704 | 0.260388799714643 | 0.740146245764223 | 11.88336008560728 | 0.00891742464776173 | 0.00535045478865704 | 0.0356696985910469 | 0.242553950419119 | 0.360263955769574 | 0.00356696985910469 | 0.0 | 0.0945247012662743 | 1.219903691813804 | 1.970750847155341 | 0.0642054574638844 | 0.0 | 0.0428036383092563 | 2.573568753344034 | 0.137328339575531 | 0.0 | 0.998751560549313 | 0.460139111824505 | 1.03442125914036 | 0.0 | 0.0 | 0.501159265204209 | 0.0927412163367219 | 0.629570180131978 | 0.0231853040841805 | 3.468878187979311 | 0.354913500980917 | 0.287141073657928 | 0.046370608168361 | 0.128410914927769 | 0.149812734082397 | 0.60103442125914 | 0.0 | 0.0624219725343321 | 1.403602639557695 | 0.176565008025682 | 0.0 | 0.00535045478865704 | 0.0 | 0.00178348492955235 | 0.490458355626895 | 0.0267522739432852 | 1.342964151952916 | 0.219368646334938 | 0.283574103798823 | 0.0 | 0.579632602104512 | 0.176565008025682 | 0.0 | 0.756197610130195 | 1.259140360263956 | 0.00535045478865704 | 0.0142678794364188 | 0.0178348492955235 | 0.0231853040841805 | 1.143213839843054 | 0.251471375066881 | 0.422685928303906 | 0.310326377742108 | 0.453005172106296 | 0.00891742464776173 | 0.00535045478865704 | 0.324594257178527 | 0.0 | 0.31210986267166 | 0.131977884786874 | 0.629570180131978 | 0.0 | 0.131977884786874 | 0.00178348492955235 | 0.0784733369003032 | 0.0 | 0.0838237916889602 | 1.153914749420367 | 0.0 | 0.955947922240057 | 0.098091671125379 | 0.0231853040841805 | 0.0160513643659711 | 9.249152844658445 |
| Sample_3.1 | 12.27199697571118 | 10.35346375578868 | 4.560060485776392 | 5.146016444570455 | 6.87080616198847 | 8.19865797183631 | 0.0708817692089595 | 1.096304697098573 | 0.420565163973159 | 1.847651450713543 | 2.008316794253851 | 1.526320763632927 | 4.158397126925621 | 0.855306681788111 | 1.842925999432946 | 0.642661374161232 | 0.160665343540308 | 1.620829789244873 | 0.0 | 0.515074189585105 | 3.388148568188261 | 3.89849730649277 | 0.888384840752292 | 0.0756072204895568 | 0.477270579340327 | 0.600132312635857 | 3.132974199036008 | 0.477270579340327 | 0.600132312635857 | 1.686986107173235 | 1.819298743029959 | 1.403459030337397 | 0.0 | 0.430016066534354 | 1.32785180984784 | 0.189018051223892 | 0.0756072204895568 | 0.378036102447784 | 0.0 | 0.0 | 0.500897835743314 | 0.779699461298554 | 0.363859748605992 | 1.597202532841887 | 0.0 | 0.363859748605992 | 0.283527076835838 | 0.557603251110481 | 1.025422927889613 | 0.264625271713449 | 0.259899820432851 | 0.0189018051223892 | 0.146488989698516 | 0.155939892259711 | 0.916737548435876 | 0.0 | 0.127587184576127 | 0.0 | 0.760797656176165 | 0.0 | 0.278801625555241 | 0.0330781589641811 | 0.0 | 0.189018051223892 | 0.538701445988092 | 0.0 | 0.316605235800019 | 0.0 | 0.0519799640865703 | 0.0189018051223892 | 0.108685379453738 | 0.0330781589641811 | 0.430016066534354 | 0.0 | 0.0 | 0.0897835743313486 | 0.0661563179283622 | 0.0283527076835838 | 0.0 | 0.0047254512805973 | 0.0756072204895568 | 0.151214440979114 | 0.255174369152254 | 0.118136282014932 | 0.047254512805973 | 0.047254512805973 | 0.0425290615253757 | 0.236272564029865 | 0.0094509025611946 | 0.0283527076835838 | 0.0378036102447784 | 0.0 | 0.0 | 0.0 | 0.0 | 0.103959928173141 | 0.0992344768925432 | 0.207919856346281 | 3.213306870806161 |
| Sample_3.2 | 3.866922139000173 | 2.763020379724787 | 7.108953144051559 | 12.61539801428323 | 0.0 | 0.217732102421181 | 25.0370144574116 | 0.792544852813099 | 0.335307437728619 | 4.17392440341404 | 5.876589444347675 | 0.879637693781571 | 1.524124716948267 | 0.0174185681936945 | 0.574812750391918 | 3.113569064622888 | 4.964291935202926 | 1.770161992684202 | 0.0239505312663299 | 0.115398014283226 | 1.001567671137432 | 0.0 | 1.519770074899843 | 0.0 | 1.315101898623933 | 0.0 | 0.0 | 1.291151367357603 | 0.581344713464553 | 0.010886605121059 | 0.00435464204842362 | 0.158944434767462 | 0.0 | 0.291761017244383 | 0.00653196307263543 | 0.696742727747779 | 0.00217732102421181 | 0.0718515937989897 | 0.0195958892179063 | 0.0 | 0.618359170876154 | 0.934070719386866 | 0.981971781919526 | 0.0 | 0.0 | 0.846977878418394 | 0.363612611043372 | 0.631423097021425 | 0.13281658247692 | 0.838268594321547 | 0.0 | 0.250391917784358 | 0.990681066016373 | 0.13281658247692 | 0.52908900888347 | 0.0 | 0.0870928409684724 | 0.00435464204842362 | 0.00217732102421181 | 0.0174185681936945 | 0.339662079777042 | 0.137171224525344 | 0.0 | 0.124107298380073 | 0.768594321546769 | 0.00870928409684724 | 0.121929977355861 | 0.0 | 0.100156767113743 | 0.00435464204842362 | 0.163299076815886 | 0.178540323985368 | 0.901410904023689 | 0.0 | 0.00435464204842362 | 0.0283051733147535 | 0.080560877895837 | 0.293938338268594 | 0.0370144574116008 | 0.124107298380073 | 0.00435464204842362 | 0.0413690994600244 | 0.594408639609824 | 0.0479010625326598 | 0.2765197700749 | 0.0 | 0.0522557045810834 | 0.219909423445393 | 0.0 | 0.30047030134123 | 0.0 | 0.0566103466295071 | 0.0 | 0.0 | 0.0 | 0.261278522905417 | 0.246037275735935 | 0.0 | 4.378592579689951 |
| Sample_3.3 | 13.56059845366001 | 9.001907822070487 | 4.222311477055928 | 4.844863942162868 | 6.838035947384276 | 7.887338086153228 | 0.0301235063761422 | 1.059343307561 | 0.336379154533588 | 1.706998694648057 | 1.932924992469123 | 1.350537202530375 | 4.56371121598554 | 0.672758309067176 | 2.033336680389598 | 0.707902399839341 | 0.281152726177327 | 1.556381162767346 | 0.0 | 0.507079023998394 | 4.367908424540617 | 3.685108946681394 | 1.014158047996787 | 0.0903705191284265 | 0.466914348830204 | 0.677778893463199 | 2.786424339793152 | 0.451852595642133 | 0.587408374334773 | 1.646751681895773 | 1.973089667637313 | 1.611607591123607 | 0.0 | 0.572346621146701 | 1.601566422331559 | 0.130535194296616 | 0.125514609900592 | 0.346420323325635 | 0.0 | 0.0 | 0.592428958730796 | 0.783211165779696 | 0.481976102018275 | 1.435887137262777 | 0.0 | 0.291193894969374 | 0.256049804197209 | 0.552264283562607 | 1.014158047996787 | 0.240988051009137 | 0.205843960236972 | 0.0100411687920474 | 0.160658700672758 | 0.225926297821066 | 0.818355256551863 | 0.0 | 0.125514609900592 | 0.0 | 0.778190581383673 | 0.0 | 0.27111155738528 | 0.0100411687920474 | 0.0 | 0.155638116276735 | 0.451852595642133 | 0.0150617531880711 | 0.170699869464806 | 0.0 | 0.0953911035244502 | 0.0451852595642133 | 0.110452856712521 | 0.0100411687920474 | 0.23094688221709 | 0.0251029219801185 | 0.0 | 0.0602470127522844 | 0.0953911035244502 | 0.0100411687920474 | 0.0 | 0.0100411687920474 | 0.105432272316498 | 0.256049804197209 | 0.240988051009137 | 0.0251029219801185 | 0.0903705191284265 | 0.0451852595642133 | 0.0803293503363792 | 0.210864544632995 | 0.0050205843960237 | 0.0451852595642133 | 0.0150617531880711 | 0.0 | 0.0 | 0.0 | 0.0 | 0.0803293503363792 | 0.120494025504569 | 0.125514609900592 | 3.58469725876092 |
| Sample_3.6 | 65.32678319400715 | 0.00542829225925524 | 0.0 | 0.0 | 28.53110411464554 | 0.0108565845185105 | 0.0 | 0.0 | 0.0 | 0.0 | 0.165562913907285 | 0.0 | 0.0 | 0.0 | 0.0 | 0.00542829225925524 | 0.0 | 0.0 | 0.0 | 0.0 | 0.0 | 0.108565845185105 | 0.0 | 0.0 | 0.0 | 4.280208446422756 | 0.00814243838888286 | 0.00542829225925524 | 0.0 | 0.119422429703615 | 0.488546303332972 | 0.0 | 0.0 | 0.0 | 0.0 | 0.00542829225925524 | 0.0 | 0.0 | 0.0 | 0.0 | 0.0 | 0.0 | 0.0 | 0.0 | 0.0 | 0.0 | 0.0 | 0.0 | 0.0 | 0.0 | 0.0 | 0.0 | 0.0 | 0.0 | 0.0 | 0.0 | 0.0 | 0.0515687764629248 | 0.0 | 0.0 | 0.0 | 0.0 | 0.0 | 0.0814243838888286 | 0.0 | 0.0 | 0.0 | 0.0 | 0.0 | 0.0 | 0.0 | 0.0 | 0.0 | 0.0 | 0.0 | 0.0 | 0.0 | 0.0 | 0.0 | 0.0 | 0.0 | 0.0 | 0.0 | 0.0 | 0.0 | 0.0 | 0.0 | 0.0 | 0.00814243838888286 | 0.0 | 0.0 | 0.0 | 0.0 | 0.0 | 0.0 | 0.0 | 0.0 | 0.0 | 0.79795896211052 |
| Sample_4.2 | 0.291055636404343 | 0.403000111944476 | 11.42953095264749 | 6.190529497369305 | 0.0 | 0.128736146871152 | 0.0 | 17.31221314228143 | 0.190305608418225 | 0.302250083958357 | 1.50005597223777 | 0.212694503526251 | 0.229486174857271 | 0.0447777902160528 | 0.744430762341878 | 0.0167916713310198 | 4.701667972685548 | 3.554237098399194 | 0.0 | 0.139930594425165 | 0.31344453151237 | 0.0 | 0.5933057203627 | 0.268666741296317 | 0.0111944475540132 | 0.0615694615470727 | 0.00559722377700661 | 1.20340311205642 | 0.867569685436024 | 0.0223888951080264 | 0.0 | 0.31344453151237 | 1.1866114407254 | 0.0391805664390462 | 0.0111944475540132 | 1.113847531624315 | 0.195902832195231 | 0.537333482592634 | 3.87327885368857 | 3.862084406134557 | 1.052278070077242 | 0.027986118885033 | 0.470166797268555 | 0.0 | 0.0 | 0.0 | 1.584014328892869 | 0.0 | 0.123138923094145 | 0.162319489533192 | 1.785514384865107 | 2.675472965409157 | 0.134333370648158 | 0.0223888951080264 | 0.212694503526251 | 0.0 | 0.996305832307176 | 0.0 | 0.0 | 1.863875517743199 | 0.0503750139930594 | 0.64368073435576 | 1.975819993283332 | 0.828389118996977 | 0.184708384641218 | 0.34702787417441 | 0.162319489533192 | 1.919847755513265 | 0.363819545505429 | 0.0335833426620396 | 0.0335833426620396 | 1.511250419791784 | 0.0 | 0.0 | 0.436583454606515 | 0.302250083958357 | 0.0503750139930594 | 0.755625209895892 | 0.901153028098063 | 0.0 | 0.0335833426620396 | 0.481361244822568 | 0.0 | 0.794805776334938 | 0.251875069965297 | 0.285458412627337 | 0.0111944475540132 | 0.0 | 0.0 | 0.027986118885033 | 0.0 | 0.425389007052502 | 0.0 | 1.136236426732341 | 0.0895555804321057 | 0.212694503526251 | 0.0167916713310198 | 0.190305608418225 | 12.56017015560283 |
| Sample_4.5 | 11.31582836592936 | 46.72643972609488 | 0.59608847726489 | 0.093600669983743 | 0.315286467313661 | 0.0 | 0.0 | 0.0443371594659835 | 0.0 | 2.290753239075816 | 0.108379723139071 | 0.881816838267895 | 2.005024878072812 | 0.487708754125819 | 0.428592541504508 | 0.374402679934972 | 0.517266860436475 | 0.187201339967486 | 0.241391201537022 | 0.497561456229371 | 0.18227498891571 | 0.15764323365683 | 0.911374944578551 | 7.813192768116655 | 0.0 | 0.468003349918715 | 0.837479678801911 | 0.20690674417459 | 0.448297945711611 | 0.433518892556284 | 0.0 | 0.251243903640573 | 0.502487807281147 | 1.078870880338933 | 0.0492635105177595 | 0.408887137297404 | 2.753830237942756 | 0.950785752992758 | 0.0 | 0.0 | 0.0197054042071038 | 0.118232425242623 | 0.403960786245628 | 0.0738952657766392 | 0.0 | 0.36454997783142 | 0.0098527021035519 | 0.758658061973496 | 0.23153849943347 | 0.0788216168284152 | 0.133011478397951 | 0.0 | 0.0344844573624316 | 0.561604019902458 | 0.0738952657766392 | 0.0 | 0.0738952657766392 | 0.0 | 0.0 | 0.113306074190847 | 0.0197054042071038 | 0.162569584708606 | 0.0 | 0.295581063106557 | 0.0 | 1.083797231390709 | 0.216759446278142 | 0.0 | 0.305433765210109 | 0.3891817330903 | 0.0197054042071038 | 0.0 | 0.0 | 0.832553327750135 | 0.0344844573624316 | 0.285728361003005 | 0.857185083009015 | 0.0147790531553278 | 0.0492635105177595 | 0.0 | 0.0098527021035519 | 0.0394108084142076 | 0.0 | 0.0640425636730873 | 0.0492635105177595 | 0.280802009951229 | 0.0098527021035519 | 0.201980393122814 | 0.0541898615695354 | 0.246317552588797 | 0.0344844573624316 | 0.0197054042071038 | 0.0 | 0.0 | 0.0788216168284152 | 0.113306074190847 | 0.0 | 0.300507414158333 | 6.315582048376769 |
| Sample_4.6 | 34.4312433831314 | 5.466023605066071 | 10.56346312198565 | 0.0117633219621221 | 0.0352899658863663 | 4.211269262439713 | 0.0 | 0.00784221464141474 | 0.0 | 1.615496216131435 | 0.576402776143983 | 6.95604438693487 | 0.0548955024899031 | 10.97517939065992 | 2.682037407363839 | 0.568560561502568 | 0.0313688585656589 | 0.0 | 0.0 | 0.631298278633886 | 0.00784221464141474 | 0.874406932517743 | 1.552758499000118 | 0.00784221464141474 | 0.0 | 0.0156844292828295 | 0.0274477512449516 | 0.396031839391444 | 0.0117633219621221 | 1.278280986550602 | 0.192134258714661 | 0.0 | 0.0 | 1.235148806022821 | 0.13331764890405 | 0.0862643610555621 | 0.348978551542956 | 0.431321805277811 | 0.0 | 0.0 | 0.0 | 0.529349488295495 | 0.0 | 0.537191702936909 | 0.0 | 0.0 | 0.0588166098106105 | 0.384268517429322 | 0.101948790338392 | 0.0 | 0.0 | 0.0 | 0.0 | 0.0235266439242442 | 0.0 | 2.611457475591107 | 0.0 | 2.454613182762812 | 0.948907971611183 | 0.0 | 0.0 | 0.0 | 0.0 | 0.0313688585656589 | 0.0 | 0.0 | 0.305846371015175 | 0.0 | 0.0 | 0.0 | 0.0 | 0.0 | 0.0 | 0.80382700074501 | 0.0 | 0.572481668823276 | 0.0 | 0.0196055366035368 | 0.00784221464141474 | 0.0 | 0.0156844292828295 | 0.0 | 0.0 | 0.0 | 0.388189624750029 | 0.0313688585656589 | 0.482296200447006 | 0.0 | 0.921460220366231 | 0.0117633219621221 | 0.145080970866173 | 0.0 | 0.0 | 0.0 | 0.0 | 0.0 | 0.0 | 0.0 | 3.195702466376505 |
| Sample_4.7 | 24.81660946227876 | 3.159701191197254 | 11.38030823070193 | 9.334410121811695 | 0.0 | 0.00336496399488525 | 0.0 | 2.419409112322498 | 2.018978396931153 | 3.16306615519214 | 4.128810821724206 | 0.504744599232788 | 1.413284877851807 | 0.00672992798977051 | 0.898445386634363 | 7.325526616865198 | 1.655562285483545 | 2.856854431657581 | 0.0807591358772462 | 1.002759270475806 | 0.0437445319335083 | 0.0100948919846558 | 1.564708257621643 | 0.0437445319335083 | 5.861767279090114 | 0.0 | 0.00672992798977051 | 0.844605962716199 | 0.864795746685511 | 0.0168248199744263 | 0.0 | 0.215357695672656 | 0.0 | 0.218722659667542 | 0.0370146039437378 | 0.373511003432263 | 0.00672992798977051 | 0.131233595800525 | 0.0 | 0.0 | 0.5249343832021 | 0.437445319335083 | 0.370146039437378 | 0.0 | 0.0 | 0.679722726966822 | 0.286021939565247 | 0.104313883841443 | 0.188437983713574 | 1.171007470220069 | 0.0 | 0.0 | 1.376270273908069 | 0.275927047580591 | 0.114408775826099 | 0.0 | 0.242277407631738 | 0.0 | 0.0168248199744263 | 0.00672992798977051 | 0.720102294905444 | 0.0302846759539673 | 0.0 | 0.121138703815869 | 0.205262803688001 | 0.0 | 0.32640150750387 | 0.0 | 0.164883235749378 | 0.0235547479641968 | 1.164277542230298 | 0.0100948919846558 | 0.107678847836328 | 0.0 | 0.0100948919846558 | 0.00672992798977051 | 0.0235547479641968 | 0.0672992798977051 | 0.0706642438925904 | 1.362810417928528 | 0.0168248199744263 | 0.0168248199744263 | 0.306211723534558 | 0.0100948919846558 | 0.100948919846558 | 0.00336496399488525 | 0.0 | 0.0302846759539673 | 0.0 | 0.0 | 0.0 | 0.572043879130493 | 0.0 | 0.0 | 0.0 | 0.164883235749378 | 0.508109563227673 | 0.0336496399488525 | 1.618547681539806 |
| Sample_4.8 | 26.53158198418355 | 5.627397074296574 | 16.28822591046397 | 0.0 | 0.00678817499915148 | 0.0 | 0.0 | 0.0 | 17.3336048603333 | 3.899806537012524 | 0.481960424939755 | 3.000373349624953 | 0.855310049893086 | 0.75688151240539 | 1.232053762345993 | 0.787428299901572 | 0.648270712418966 | 0.0 | 0.0 | 8.342667073957166 | 1.7208023622849 | 0.105216712486848 | 0.543053999932118 | 0.0644876624919391 | 0.0 | 1.160777924854903 | 0.0509113124936361 | 0.519295387435088 | 0.0373349624953331 | 0.264738824966908 | 0.0814580999898177 | 0.556630349930421 | 0.0 | 0.224009774971999 | 0.889250924888844 | 0.0475172249940604 | 0.0237586124970302 | 0.0610935749923633 | 0.0 | 0.0 | 0.00339408749957574 | 0.0203645249974544 | 0.0 | 0.013576349998303 | 0.0 | 0.308861962461392 | 0.013576349998303 | 0.105216712486848 | 0.0712758374910905 | 0.091640362488545 | 0.0 | 0.0 | 0.0101822624987272 | 0.0509113124936361 | 0.0 | 0.0 | 0.946950412381631 | 0.0 | 0.0 | 0.0 | 0.702576112412178 | 0.0 | 0.0 | 0.0610935749923633 | 0.00678817499915148 | 0.0 | 0.152733937480908 | 0.0 | 0.00678817499915148 | 0.0169704374978787 | 0.156128024980484 | 0.0 | 0.0 | 0.0 | 0.0 | 0.013576349998303 | 0.0 | 0.0339408749957574 | 0.0 | 0.0 | 1.140413399857448 | 0.091640362488545 | 0.0 | 0.0 | 0.0 | 0.0 | 0.590571224926179 | 0.18328072497709 | 0.20703933747412 | 0.458201812442725 | 0.96392084987951 | 0.0 | 0.0 | 0.0 | 0.0101822624987272 | 0.00678817499915148 | 0.0 | 0.13576349998303 | 1.282965074839628 |Relative abundance (%)
SV1
SV2
SV3
SV4
SV5
SV6
SV7
SV8
SV9
SV10
Supplementary Figure S2 (d). Relative abundance of bacterial 16S rRNA genes at family taxonomic rank.

## Slide 5
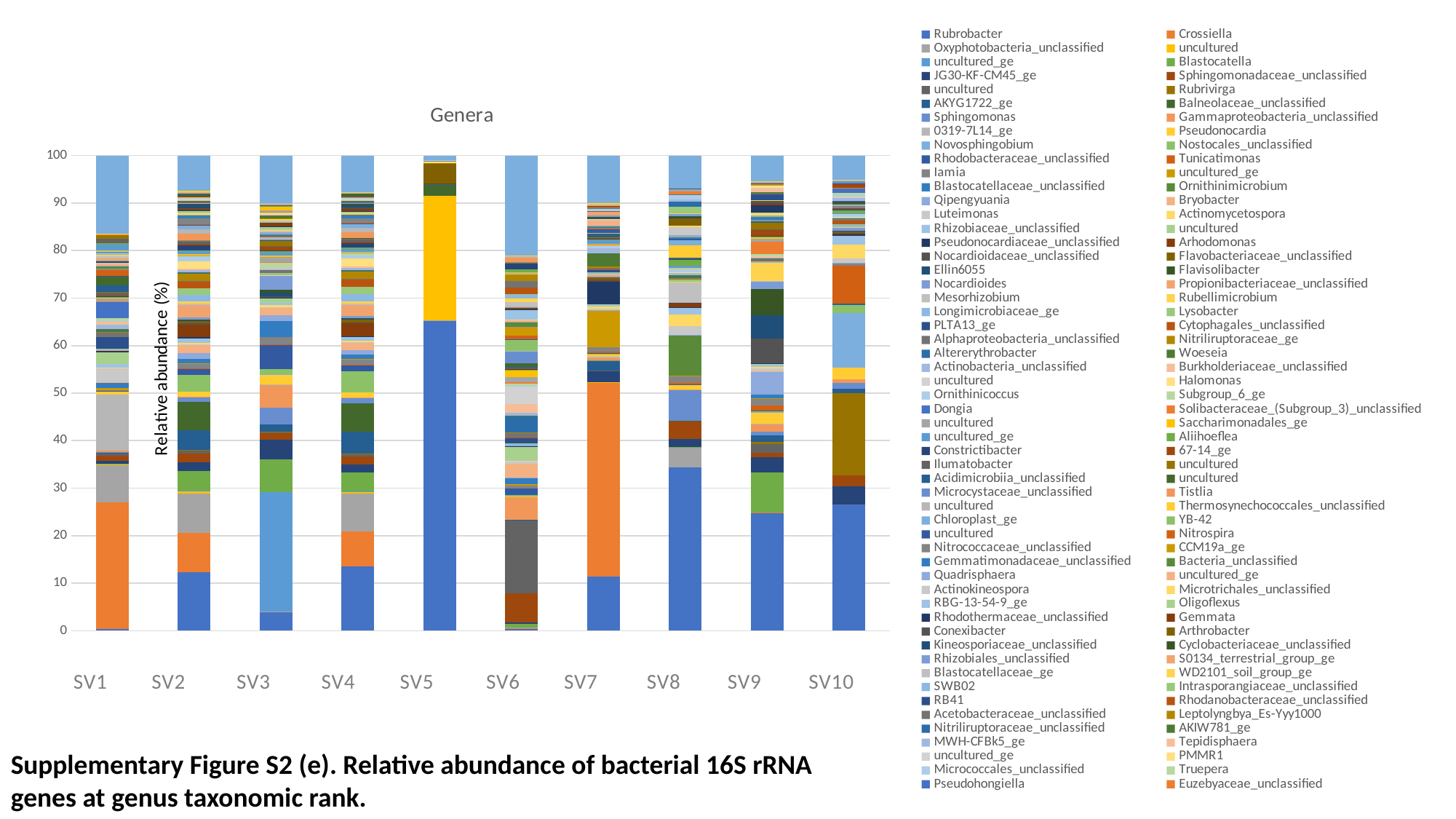

### Chart: Genera
| Category | Rubrobacter | Crossiella | Oxyphotobacteria_unclassified | uncultured | uncultured_ge | Blastocatella | JG30-KF-CM45_ge | Sphingomonadaceae_unclassified | uncultured | Rubrivirga | AKYG1722_ge | Balneolaceae_unclassified | Sphingomonas | Gammaproteobacteria_unclassified | 0319-7L14_ge | Pseudonocardia | Novosphingobium | Nostocales_unclassified | Rhodobacteraceae_unclassified | Tunicatimonas | Iamia | uncultured_ge | Blastocatellaceae_unclassified | Ornithinimicrobium | Qipengyuania | Bryobacter | Luteimonas | Actinomycetospora | Rhizobiaceae_unclassified | uncultured | Pseudonocardiaceae_unclassified | Arhodomonas | Nocardioidaceae_unclassified | Flavobacteriaceae_unclassified | Ellin6055 | Flavisolibacter | Nocardioides | Propionibacteriaceae_unclassified | Mesorhizobium | Rubellimicrobium | Longimicrobiaceae_ge | Lysobacter | PLTA13_ge | Cytophagales_unclassified | Alphaproteobacteria_unclassified | Nitriliruptoraceae_ge | Altererythrobacter | Woeseia | Actinobacteria_unclassified | Burkholderiaceae_unclassified | uncultured | Halomonas | Ornithinicoccus | Subgroup_6_ge | Dongia | Solibacteraceae_(Subgroup_3)_unclassified | uncultured | Saccharimonadales_ge | uncultured_ge | Aliihoeflea | Constrictibacter | 67-14_ge | Ilumatobacter | uncultured | Acidimicrobiia_unclassified | uncultured | Microcystaceae_unclassified | Tistlia | uncultured | Thermosynechococcales_unclassified | Chloroplast_ge | YB-42 | uncultured | Nitrospira | Nitrococcaceae_unclassified | CCM19a_ge | Gemmatimonadaceae_unclassified | Bacteria_unclassified | Quadrisphaera | uncultured_ge | Actinokineospora | Microtrichales_unclassified | RBG-13-54-9_ge | Oligoflexus | Rhodothermaceae_unclassified | Gemmata | Conexibacter | Arthrobacter | Kineosporiaceae_unclassified | Cyclobacteriaceae_unclassified | Rhizobiales_unclassified | S0134_terrestrial_group_ge | Blastocatellaceae_ge | WD2101_soil_group_ge | SWB02 | Intrasporangiaceae_unclassified | RB41 | Rhodanobacteraceae_unclassified | Acetobacteraceae_unclassified | Leptolyngbya_Es-Yyy1000 | Nitriliruptoraceae_unclassified | AKIW781_ge | MWH-CFBk5_ge | Tepidisphaera | uncultured_ge | PMMR1 | Micrococcales_unclassified | Truepera | Pseudohongiella | Euzebyaceae_unclassified | uncultured | JGI_0001001-H03 | Latescibacteraceae_ge | A4b_ge | Obscuribacterales_ge | Geminicoccaceae_unclassified | MND1 | Hyphomicrobium | Xanthomonadaceae_unclassified | Haloactinopolyspora | Brevundimonas | Candidatus_Alysiosphaera | JG30-KF-CM66_ge | Gemmatimonadetes_unclassified | Remaining genera |
|---|---|---|---|---|---|---|---|---|---|---|---|---|---|---|---|---|---|---|---|---|---|---|---|---|---|---|---|---|---|---|---|---|---|---|---|---|---|---|---|---|---|---|---|---|---|---|---|---|---|---|---|---|---|---|---|---|---|---|---|---|---|---|---|---|---|---|---|---|---|---|---|---|---|---|---|---|---|---|---|---|---|---|---|---|---|---|---|---|---|---|---|---|---|---|---|---|---|---|---|---|---|---|---|---|---|---|---|---|---|---|---|---|---|---|---|---|---|---|---|---|---|---|---|---|---|
| Sample_2.1 | 0.422685928303906 | 26.5382557517389 | 7.963260210451222 | 0.0142678794364188 | 0.0 | 0.244337435348671 | 0.574282147315855 | 1.077224897449616 | 0.376315320135545 | 0.0 | 0.37988228999465 | 0.0 | 0.00891742464776173 | 0.260388799714643 | 11.88336008560728 | 0.583199571963617 | 0.0 | 0.00535045478865704 | 0.108792580702693 | 0.0 | 0.242553950419119 | 0.360263955769574 | 1.144997324772606 | 0.0 | 0.0 | 0.146245764223292 | 2.926698769395398 | 0.0338862136614946 | 0.766898519707508 | 2.577135723203139 | 0.356696985910469 | 0.0303192438023899 | 0.00535045478865704 | 0.0 | 0.00891742464776173 | 0.0 | 0.0 | 0.046370608168361 | 0.130194399857321 | 0.0 | 0.0410201533797039 | 0.0338862136614946 | 2.573568753344034 | 0.0 | 0.998751560549313 | 0.0552880328161227 | 0.0321027287319422 | 0.458355626894953 | 1.03442125914036 | 0.633137149991082 | 0.0 | 0.0 | 0.0285357588728375 | 0.629570180131978 | 3.468878187979311 | 0.137328339575531 | 0.52434456928839 | 0.287141073657928 | 0.046370608168361 | 0.0891742464776173 | 0.128410914927769 | 0.149812734082397 | 0.634920634920635 | 0.0624219725343321 | 1.403602639557695 | 1.844123417157125 | 0.0 | 0.0 | 0.140895309434635 | 0.0 | 0.00178348492955235 | 0.0 | 0.0214018191546281 | 1.342964151952916 | 0.00535045478865704 | 0.219368646334938 | 0.123060460139112 | 0.283574103798823 | 0.0909577314071696 | 0.579632602104512 | 0.0659889423934368 | 0.176565008025682 | 0.0 | 0.0 | 0.0481540930979133 | 0.057071517745675 | 0.0107009095773141 | 0.0 | 0.00178348492955235 | 0.00891742464776173 | 0.251471375066881 | 0.422685928303906 | 0.461922596754058 | 0.310326377742108 | 0.52434456928839 | 0.0 | 0.00891742464776173 | 0.0321027287319422 | 0.0142678794364188 | 0.0 | 0.00891742464776173 | 0.0 | 0.0 | 0.00535045478865704 | 0.131977884786874 | 0.0 | 0.00178348492955235 | 0.0784733369003032 | 0.0 | 0.00178348492955235 | 0.0713393971820938 | 0.0713393971820938 | 1.153914749420367 | 0.294275013376137 | 0.0 | 0.0 | 0.916711253789906 | 0.820403067594079 | 0.155163188871054 | 0.0142678794364188 | 0.0267522739432852 | 0.00535045478865704 | 0.098091671125379 | 0.0160513643659711 | 16.41876226145892 |
| Sample_3.1 | 12.27199697571118 | 8.368774217937816 | 8.19865797183631 | 0.500897835743314 | 0.0708817692089595 | 4.115868065400246 | 1.847651450713543 | 1.965787732728476 | 0.637935922880635 | 0.0850581230507513 | 4.158397126925621 | 5.99659767507797 | 0.82222852282393 | 0.160665343540308 | 0.0 | 1.186088271429922 | 0.0 | 3.388148568188261 | 1.365655420092619 | 0.151214440979114 | 0.888384840752292 | 0.0756072204895568 | 0.997070220206029 | 0.0 | 1.242793686797089 | 1.583026179000095 | 0.160665343540308 | 0.392212456289576 | 0.812777620262735 | 0.0 | 0.311879784519422 | 2.717134486343446 | 0.217370758907476 | 0.500897835743314 | 0.217370758907476 | 0.127587184576127 | 0.387487005008978 | 2.561194594083735 | 0.387487005008978 | 0.500897835743314 | 1.375106322653814 | 1.370380871373217 | 0.0 | 1.32785180984784 | 0.189018051223892 | 1.549948020035913 | 0.250448917871657 | 0.0756072204895568 | 0.378036102447784 | 0.373310651167186 | 0.0 | 1.483791702107551 | 0.841130327946319 | 0.363859748605992 | 0.0 | 0.0378036102447784 | 0.0189018051223892 | 0.283527076835838 | 0.557603251110481 | 0.255174369152254 | 1.025422927889613 | 0.264625271713449 | 0.434741517814951 | 0.146488989698516 | 0.155939892259711 | 0.0094509025611946 | 0.0094509025611946 | 1.384557225215008 | 0.916737548435876 | 0.0 | 0.760797656176165 | 0.0 | 0.302428881958227 | 0.0330781589641811 | 1.153010112465741 | 0.0 | 0.505623287023911 | 0.189018051223892 | 0.127587184576127 | 0.0 | 0.0 | 0.316605235800019 | 0.0 | 0.486721481901522 | 0.0945090256119459 | 0.307154333238824 | 0.259899820432851 | 0.0 | 0.652112276722427 | 0.274076174274643 | 0.0897835743313486 | 0.0661563179283622 | 0.0141763538417919 | 0.0283527076835838 | 0.0 | 0.0141763538417919 | 0.0047254512805973 | 0.0 | 0.0141763538417919 | 0.0 | 0.132312635856724 | 0.255174369152254 | 0.0425290615253757 | 0.047254512805973 | 0.236272564029865 | 0.250448917871657 | 0.0094509025611946 | 0.0283527076835838 | 0.0378036102447784 | 0.047254512805973 | 0.0330781589641811 | 0.0047254512805973 | 0.0 | 0.118136282014932 | 0.0 | 0.0094509025611946 | 0.0 | 0.0 | 0.240998015310462 | 0.5245250921463 | 0.0283527076835838 | 0.0141763538417919 | 0.103959928173141 | 0.207919856346281 | 7.480389377185534 |
| Sample_3.2 | 3.866922139000173 | 0.00435464204842362 | 0.217732102421181 | 0.0 | 25.0370144574116 | 6.934767462114615 | 4.17392440341404 | 1.306392614527086 | 0.185072287058004 | 0.0892701619926842 | 1.524124716948267 | 0.0 | 3.551210590489462 | 4.964291935202926 | 0.0239505312663299 | 2.146838529872844 | 0.00217732102421181 | 1.001567671137432 | 5.286535446786273 | 0.0174185681936945 | 1.519770074899843 | 0.0 | 3.32041456192301 | 0.0 | 1.236718341752308 | 1.589444347674621 | 0.115398014283226 | 0.370144574116008 | 0.579167392440341 | 0.960198571677408 | 0.113220693259014 | 0.0 | 0.248214596760146 | 0.0 | 0.80560877895837 | 0.618359170876154 | 2.810921442257446 | 0.0 | 0.00435464204842362 | 0.209022818324334 | 0.130639261452709 | 0.370144574116008 | 0.0 | 0.00653196307263543 | 0.696742727747779 | 0.00870928409684724 | 0.0 | 0.00217732102421181 | 0.0718515937989897 | 0.285229054171747 | 0.0 | 0.00435464204842362 | 0.00653196307263543 | 0.981971781919526 | 0.0 | 0.176363002961157 | 1.073419264936422 | 0.363612611043372 | 0.631423097021425 | 0.2765197700749 | 0.13281658247692 | 0.838268594321547 | 0.108866051210591 | 0.990681066016373 | 0.13281658247692 | 0.00217732102421181 | 0.172008360912733 | 0.0 | 0.52908900888347 | 0.0 | 0.00217732102421181 | 0.0 | 0.0566103466295071 | 0.137171224525344 | 0.0 | 0.0 | 0.206845497300122 | 0.124107298380073 | 0.718515937989897 | 0.00870928409684724 | 0.128461940428497 | 0.121929977355861 | 0.0 | 0.648841665215119 | 0.100156767113743 | 0.398449747430761 | 0.169831039888521 | 0.0 | 0.209022818324334 | 0.0696742727747779 | 0.0283051733147535 | 0.080560877895837 | 0.265633164953841 | 0.293938338268594 | 0.0 | 0.0 | 0.117575335307438 | 0.0 | 0.0870928409684724 | 0.0195958892179063 | 0.00217732102421181 | 0.594408639609824 | 0.0522557045810834 | 0.0479010625326598 | 0.219909423445393 | 0.128461940428497 | 0.0 | 0.30047030134123 | 0.0 | 0.0370144574116008 | 0.267810485978053 | 0.949311966556349 | 0.0 | 0.034837136387389 | 0.0 | 0.0195958892179063 | 0.0 | 0.0 | 0.0674969517505661 | 0.0 | 0.169831039888521 | 0.0522557045810834 | 0.261278522905417 | 0.0 | 9.974307611914313 |
| Sample_3.3 | 13.56059845366001 | 7.375238477758811 | 7.887338086153228 | 0.507079023998394 | 0.0301235063761422 | 3.865849984938246 | 1.706998694648057 | 1.742142785420223 | 0.632593633898986 | 0.050205843960237 | 4.56371121598554 | 6.009639522040367 | 0.938849282056432 | 0.281152726177327 | 0.0 | 1.009137463600763 | 0.0 | 4.367908424540617 | 1.430866552866754 | 0.0753087659403555 | 1.014158047996787 | 0.0903705191284265 | 0.953911035244503 | 0.0 | 0.973993372828597 | 1.511195903203133 | 0.110452856712521 | 0.316296816949493 | 0.738025906215484 | 0.0 | 0.246008635405161 | 2.675971483080631 | 0.23094688221709 | 0.592428958730796 | 0.220905713425043 | 0.0953911035244502 | 0.446832011246109 | 2.234160056230546 | 0.316296816949493 | 0.456873180038156 | 1.586504669143488 | 1.541319409579275 | 0.0 | 1.601566422331559 | 0.130535194296616 | 1.516216487599156 | 0.195802791444924 | 0.125514609900592 | 0.346420323325635 | 0.331358570137564 | 0.0 | 1.691936941459986 | 0.672758309067176 | 0.481976102018275 | 0.0 | 0.0451852595642133 | 0.0200823375840948 | 0.256049804197209 | 0.552264283562607 | 0.256049804197209 | 1.014158047996787 | 0.240988051009137 | 0.396626167285872 | 0.160658700672758 | 0.225926297821066 | 0.0050205843960237 | 0.0 | 1.285269605382066 | 0.818355256551863 | 0.0 | 0.778190581383673 | 0.0 | 0.351440907721659 | 0.0100411687920474 | 0.933828697660408 | 0.0 | 0.517120192790441 | 0.155638116276735 | 0.175720453860829 | 0.0150617531880711 | 0.0 | 0.170699869464806 | 0.0 | 0.421729089265991 | 0.0903705191284265 | 0.411687920473943 | 0.210864544632995 | 0.0 | 0.607490711918867 | 0.336379154533588 | 0.0602470127522844 | 0.0953911035244502 | 0.0 | 0.0100411687920474 | 0.0 | 0.0 | 0.0100411687920474 | 0.0 | 0.0100411687920474 | 0.0 | 0.115473441108545 | 0.240988051009137 | 0.0803293503363792 | 0.0753087659403555 | 0.210864544632995 | 0.235967466613114 | 0.0050205843960237 | 0.0451852595642133 | 0.0150617531880711 | 0.0853499347324028 | 0.050205843960237 | 0.0 | 0.0 | 0.0251029219801185 | 0.0 | 0.0200823375840948 | 0.0 | 0.0 | 0.246008635405161 | 0.50205843960237 | 0.0251029219801185 | 0.0150617531880711 | 0.0803293503363792 | 0.125514609900592 | 7.671452957124215 |
| Sample_3.6 | 65.32678319400715 | 0.00542829225925524 | 0.0108565845185105 | 26.15079795896211 | 0.0 | 0.0 | 0.0 | 0.0 | 0.0 | 0.0 | 0.0 | 2.347736402127891 | 0.0 | 0.0 | 0.0 | 0.0 | 0.0 | 0.0 | 0.132993160351753 | 0.0 | 0.0 | 0.0 | 0.0 | 0.0 | 0.0 | 0.0 | 0.0 | 0.0 | 0.0 | 0.0 | 0.0 | 0.100423406796222 | 0.00271414612962762 | 4.280208446422756 | 0.0 | 0.0 | 0.00271414612962762 | 0.0 | 0.0 | 0.0 | 0.0 | 0.0 | 0.0 | 0.0 | 0.00542829225925524 | 0.116708283573988 | 0.0 | 0.0 | 0.0 | 0.0 | 0.0 | 0.38812289653675 | 0.0 | 0.0 | 0.0 | 0.0 | 0.00542829225925524 | 0.0 | 0.0 | 0.0 | 0.0 | 0.0 | 0.0 | 0.0 | 0.0 | 0.0 | 0.0 | 0.0 | 0.0 | 0.0 | 0.0 | 0.0 | 0.0 | 0.0 | 0.00814243838888286 | 0.0 | 0.0 | 0.0814243838888286 | 0.0 | 0.0 | 0.0 | 0.0 | 0.0 | 0.0 | 0.0 | 0.0 | 0.0 | 0.0352838996851591 | 0.0 | 0.0 | 0.0 | 0.0 | 0.0 | 0.0 | 0.0 | 0.0 | 0.0 | 0.0 | 0.0 | 0.0 | 0.00271414612962762 | 0.0 | 0.0 | 0.0 | 0.0 | 0.0 | 0.00814243838888286 | 0.0 | 0.0 | 0.0 | 0.0 | 0.0 | 0.0 | 0.0 | 0.0 | 0.0 | 0.0 | 0.0 | 0.0 | 0.0 | 0.0 | 0.0 | 0.0 | 0.0 | 0.987949191184453 |
| Sample_4.2 | 0.291055636404343 | 0.190305608418225 | 0.128736146871152 | 0.0 | 0.0 | 0.755625209895892 | 0.302250083958357 | 6.207321168700325 | 15.28042091122803 | 0.0 | 0.229486174857271 | 0.0 | 0.0 | 4.701667972685548 | 0.0 | 0.128736146871152 | 0.0 | 0.31344453151237 | 1.432889286913691 | 0.0 | 0.5933057203627 | 0.268666741296317 | 1.376917049143625 | 0.0 | 0.0111944475540132 | 2.988917496921527 | 0.453375125937535 | 0.0559722377700661 | 0.123138923094145 | 2.983320273144521 | 0.027986118885033 | 0.0 | 0.0 | 0.0 | 0.0559722377700661 | 0.0 | 0.0167916713310198 | 0.0 | 0.00559722377700661 | 0.0 | 0.31344453151237 | 0.207097279749244 | 1.1866114407254 | 0.0111944475540132 | 1.113847531624315 | 0.0223888951080264 | 3.27997313332587 | 0.195902832195231 | 0.537333482592634 | 1.886264412851226 | 3.834098287249524 | 0.0 | 0.0335833426620396 | 0.470166797268555 | 0.0 | 0.335833426620396 | 0.968319713422143 | 1.584014328892869 | 0.0 | 0.0671666853240793 | 0.123138923094145 | 0.162319489533192 | 0.134333370648158 | 0.134333370648158 | 0.0223888951080264 | 0.722041867233852 | 2.597111832531065 | 0.0 | 0.0839583566550991 | 0.0 | 0.0 | 2.412403447889846 | 0.0391805664390462 | 0.64368073435576 | 0.0 | 1.975819993283332 | 0.106347251763125 | 0.828389118996977 | 0.027986118885033 | 0.34702787417441 | 0.0 | 0.162319489533192 | 1.919847755513265 | 0.156722265756185 | 0.0503750139930594 | 0.223888951080264 | 0.0 | 0.0 | 0.0 | 0.111944475540132 | 0.302250083958357 | 0.0503750139930594 | 0.794805776334938 | 0.755625209895892 | 0.968319713422143 | 0.0111944475540132 | 0.0 | 1.432889286913691 | 1.343333706481585 | 1.438486510690697 | 0.0 | 0.0 | 0.0111944475540132 | 0.00559722377700661 | 0.0 | 0.0 | 0.0 | 0.027986118885033 | 0.0 | 0.0 | 0.246277846188291 | 0.128736146871152 | 0.0 | 0.62688906302474 | 1.136236426732341 | 0.00559722377700661 | 0.0 | 0.285458412627337 | 0.0391805664390462 | 0.0 | 0.0503750139930594 | 0.962722489645136 | 0.212694503526251 | 0.190305608418225 | 21.01757528265986 |
| Sample_4.5 | 11.31582836592936 | 40.76062860239421 | 0.0 | 0.266022956795901 | 0.0 | 0.0 | 2.290753239075816 | 0.0492635105177595 | 0.0295581063106557 | 0.0 | 2.005024878072812 | 0.0443371594659835 | 0.103453372087295 | 0.517266860436475 | 0.241391201537022 | 0.527119562540027 | 0.0 | 0.18227498891571 | 0.103453372087295 | 0.310360116261885 | 0.911374944578551 | 7.813192768116655 | 0.0098527021035519 | 0.0 | 0.0443371594659835 | 0.0 | 0.280802009951229 | 0.369476328883196 | 0.384255382038524 | 0.0640425636730873 | 4.985467264397261 | 0.0689689147248633 | 0.295581063106557 | 0.468003349918715 | 0.00492635105177595 | 0.0 | 0.0591162126213114 | 0.3891817330903 | 0.18227498891571 | 0.0 | 0.251243903640573 | 0.108379723139071 | 0.502487807281147 | 0.0492635105177595 | 0.408887137297404 | 0.320212818365437 | 0.0 | 2.753830237942756 | 0.950785752992758 | 0.00492635105177595 | 0.0 | 0.0 | 0.482782403074043 | 0.403960786245628 | 0.0 | 0.0147790531553278 | 0.0541898615695354 | 0.0098527021035519 | 0.758658061973496 | 0.216759446278142 | 0.23153849943347 | 0.0788216168284152 | 0.142864180501503 | 0.0344844573624316 | 0.561604019902458 | 0.251243903640573 | 0.0 | 0.0 | 0.0344844573624316 | 0.0 | 0.0 | 0.0 | 0.798068870387704 | 0.162569584708606 | 0.0738952657766392 | 0.0 | 0.187201339967486 | 0.295581063106557 | 0.093600669983743 | 1.083797231390709 | 0.0394108084142076 | 0.216759446278142 | 0.0 | 0.0 | 0.0 | 0.0 | 0.241391201537022 | 0.0 | 0.0246317552588798 | 0.147790531553278 | 0.285728361003005 | 0.857185083009015 | 0.0197054042071038 | 0.0147790531553278 | 0.0098527021035519 | 0.00492635105177595 | 0.0 | 0.0147790531553278 | 0.0 | 0.0 | 0.103453372087295 | 0.0 | 0.0098527021035519 | 0.0197054042071038 | 0.201980393122814 | 0.0 | 0.0541898615695354 | 0.246317552588797 | 0.0344844573624316 | 0.236464850485246 | 0.0197054042071038 | 0.0 | 0.0 | 0.0295581063106557 | 0.0 | 0.0443371594659835 | 0.20690674417459 | 0.0 | 0.0147790531553278 | 0.0492635105177595 | 0.0197054042071038 | 0.0147790531553278 | 0.113306074190847 | 0.300507414158333 | 10.00541898615695 |
| Sample_4.6 | 34.4312433831314 | 0.0 | 4.211269262439713 | 0.0117633219621221 | 0.0 | 0.0117633219621221 | 1.615496216131435 | 3.834842959651805 | 0.0 | 0.0 | 0.0548955024899031 | 0.0235266439242442 | 6.477669293808573 | 0.0313688585656589 | 0.0 | 1.105752264439478 | 0.0117633219621221 | 0.00784221464141474 | 0.07450103909344 | 0.247029761204564 | 1.552758499000118 | 0.00784221464141474 | 0.0 | 8.46567070540721 | 0.0156844292828295 | 0.0 | 1.976238089636513 | 2.38403325099008 | 1.301807630474846 | 0.0 | 0.239187546563149 | 0.815590322707132 | 0.0470532878484884 | 0.0 | 0.0705799317727326 | 0.0 | 0.188213151393954 | 0.0196055366035368 | 4.211269262439713 | 0.00784221464141474 | 0.0 | 0.454848449202055 | 0.0 | 0.13331764890405 | 0.0862643610555621 | 0.266635297808101 | 0.0431321805277811 | 0.348978551542956 | 0.431321805277811 | 0.0 | 0.0 | 0.160765400149002 | 0.933223542328354 | 0.0 | 0.0 | 0.0 | 0.00784221464141474 | 0.0588166098106105 | 0.384268517429322 | 1.297886523154139 | 0.101948790338392 | 0.0 | 0.3725051954672 | 0.0 | 0.0235266439242442 | 0.0 | 0.0 | 0.0 | 0.0 | 2.611457475591107 | 0.948907971611183 | 0.0 | 0.525428380974787 | 0.0 | 0.0588166098106105 | 0.0 | 0.0117633219621221 | 0.0313688585656589 | 0.466611771164177 | 0.0 | 1.737050543073364 | 0.305846371015175 | 0.0 | 0.0 | 0.0 | 0.0 | 0.0 | 1.63902286005568 | 0.0627377171313179 | 0.258793083166686 | 0.572481668823276 | 0.0 | 0.0 | 0.0196055366035368 | 0.0 | 1.462573030623848 | 0.0 | 0.0 | 0.0 | 0.0 | 0.984197937497549 | 0.0 | 0.482296200447006 | 0.0 | 0.0 | 0.0 | 0.921460220366231 | 0.0117633219621221 | 0.145080970866173 | 0.662667137199545 | 0.384268517429322 | 0.0 | 0.0 | 0.0 | 0.0 | 0.0 | 0.0 | 0.0 | 0.164686507469709 | 0.0 | 0.0 | 0.0 | 0.0 | 0.0 | 6.971728816217694 |
| Sample_4.7 | 24.81660946227876 | 0.0942189918567871 | 0.00336496399488525 | 0.0 | 0.0 | 8.36193552728986 | 3.16306615519214 | 0.962379702537183 | 1.884379837135743 | 0.393700787401575 | 1.413284877851807 | 0.0 | 0.747022006864527 | 1.655562285483545 | 0.0807591358772462 | 2.466518608250891 | 0.00672992798977051 | 0.0437445319335083 | 0.235547479641968 | 0.996029342486035 | 1.564708257621643 | 0.0437445319335083 | 0.713372366915674 | 0.0 | 4.734504340803553 | 0.168248199744263 | 0.612423447069116 | 0.423985463355542 | 0.32640150750387 | 0.195167911703345 | 0.174978127734033 | 0.0 | 5.279628507974966 | 0.0 | 4.88592772057339 | 5.454606635708997 | 1.756511205330103 | 0.0 | 0.00672992798977051 | 3.832693990174304 | 0.215357695672656 | 0.174978127734033 | 0.0 | 0.0370146039437378 | 0.373511003432263 | 0.0168248199744263 | 0.0 | 0.00672992798977051 | 0.131233595800525 | 0.400430715391345 | 0.0 | 0.0 | 0.00672992798977051 | 0.370146039437378 | 0.0 | 2.678511339928663 | 0.575408843125379 | 0.286021939565247 | 0.104313883841443 | 0.0403795679386231 | 0.188437983713574 | 1.171007470220069 | 0.211992731677771 | 1.376270273908069 | 0.275927047580591 | 0.0100948919846558 | 0.0 | 0.0 | 0.107678847836328 | 0.0 | 0.0168248199744263 | 0.0 | 0.185073019718689 | 0.0302846759539673 | 0.0100948919846558 | 0.0 | 0.501379635237903 | 0.121138703815869 | 0.33313143549364 | 0.0 | 0.0 | 0.32640150750387 | 0.0 | 0.191802947708459 | 1.514233797698364 | 0.376875967427149 | 0.575408843125379 | 0.0 | 0.104313883841443 | 0.0 | 0.00672992798977051 | 0.0235547479641968 | 0.0168248199744263 | 0.0672992798977051 | 0.0 | 0.0 | 1.339255669964332 | 0.0 | 0.00672992798977051 | 0.0 | 0.0 | 0.306211723534558 | 0.0 | 0.891715458644593 | 0.0302846759539673 | 0.598963591089575 | 0.0 | 0.0 | 0.0 | 0.026919711959082 | 0.0975839558516724 | 0.00336496399488525 | 0.0 | 0.0100948919846558 | 0.0 | 0.164883235749378 | 0.0 | 0.0 | 0.0908540278619019 | 0.0 | 0.114408775826099 | 0.0 | 0.164883235749378 | 0.0336496399488525 | 5.461336563698757 |
| Sample_4.8 | 26.53158198418355 | 0.00339408749957574 | 0.0 | 0.0 | 0.0 | 0.0 | 3.899806537012524 | 2.280826799714897 | 0.0 | 17.3336048603333 | 0.855310049893086 | 0.0 | 1.306723687336659 | 0.648270712418966 | 0.0 | 2.477683874690289 | 11.44825713606897 | 1.7208023622849 | 0.373349624953331 | 7.898041611512745 | 0.543053999932118 | 0.0644876624919391 | 0.0 | 0.00678817499915148 | 0.0441231374944846 | 0.0 | 0.970709024878661 | 2.888368462138954 | 1.870142212266232 | 0.0 | 0.257950649967756 | 0.101822624987272 | 0.18328072497709 | 0.485354512439331 | 0.0509113124936361 | 0.0 | 0.580388962427451 | 0.0509113124936361 | 0.0 | 0.0101822624987272 | 0.556630349930421 | 0.149339849981333 | 0.0 | 0.889250924888844 | 0.0475172249940604 | 0.156128024980484 | 0.196857074975393 | 0.0237586124970302 | 0.0610935749923633 | 0.0 | 0.0 | 0.0678817499915148 | 0.743305162407087 | 0.0 | 0.0 | 0.0 | 0.0237586124970302 | 0.013576349998303 | 0.105216712486848 | 0.631300274921087 | 0.0712758374910905 | 0.091640362488545 | 0.488748599938906 | 0.0101822624987272 | 0.0509113124936361 | 0.0 | 0.0 | 0.00678817499915148 | 0.0 | 0.0 | 0.0 | 0.0 | 0.0950344499881207 | 0.0 | 0.00339408749957574 | 0.0 | 0.0339408749957574 | 0.0610935749923633 | 0.0203645249974544 | 0.0 | 0.0 | 0.152733937480908 | 0.0 | 0.00678817499915148 | 0.0 | 0.0 | 0.295285612463089 | 0.0 | 0.0 | 0.420866849947392 | 0.013576349998303 | 0.0 | 0.0 | 0.0339408749957574 | 0.0 | 0.00678817499915148 | 0.0 | 0.0 | 0.0 | 0.0 | 0.0984285374876965 | 0.0 | 0.590571224926179 | 0.152733937480908 | 0.18328072497709 | 0.0 | 0.20703933747412 | 0.458201812442725 | 0.96392084987951 | 0.0984285374876965 | 0.0 | 0.0 | 0.0 | 0.0 | 0.0 | 0.872280487390965 | 0.0 | 0.0 | 0.0746699249906663 | 0.0 | 0.631300274921087 | 0.0 | 0.00678817499915148 | 0.13576349998303 | 5.111495774361055 |Supplementary Figure S2 (e). Relative abundance of bacterial 16S rRNA genes at genus taxonomic rank.
